# One-Step Synthesis of [^18^F]Aromatic Electrophile Prosthetic Groups via Organic Photoredox Catalysis

**DOI:** 10.1021/acscentsci.4c00407

**Published:** 2024-07-18

**Authors:** Manshu Li, Carla Staton, Xinrui Ma, Weiling Zhao, Liqin Pan, Ben Giglio, Haiden S. Berton, Zhanhong Wu, David A. Nicewicz, Zibo Li

**Affiliations:** † Department of Radiology, Biomedical Research Imaging Center and Lineberger Comprehensive Cancer Center, University of North Carolina at Chapel Hill, Chapel Hill, North Carolina 27599 United States; ‡ Department of Chemistry University of North Carolina at Chapel Hill, Chapel Hill, North Carolina 27599 United States

## Abstract

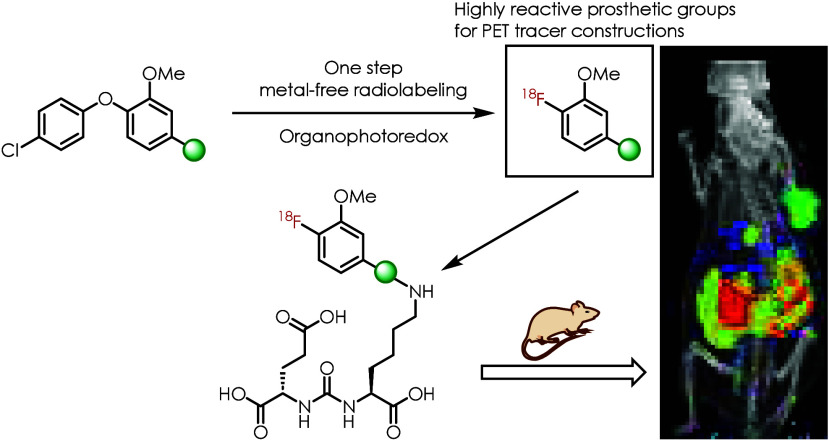

To avoid the harsh conditions that are oftentimes adopted in direct radiofluorination reactions, conjugation of bioactive ligands with ^18^F-labeled prosthetic groups has become an important strategy to construct novel PET agents under mild conditions when the ligands are structurally sensitive. Prosthetic groups with [^18^F]fluoroarene motifs are especially appealing because of their stability in physiological environments. However, their preparation can be intricate, often requiring multistep radiosynthesis with functional group conversions to prevent the decomposition of unprotected reactive prosthetic groups during the harsh radiofluorination. Here, we report a general and simple method to generate a variety of highly reactive ^18^F-labeled electrophiles via one-step organophotoredox-mediated radiofluorination. The method benefits from high step-economy, reaction efficiency, functional group tolerance, and easily accessible precursors. The obtained prosthetic groups have been successfully applied in PET agent construction and subsequent imaging studies, thereby demonstrating the feasibility of this synthetic method in promoting imaging and biomedical research.

## Abstract 2

One-step metal-free organophotoredox approach is presented to prepare robust prosthetic groups with ^18^F-labeled arene motifs. PET tracer constructions and imaging demonstrate the utility of the method.

## Introduction

Positron emission tomography (PET) is a noninvasive molecular imaging technique that provides real-time biodistribution information. Among various available positron-emitting radionuclides, fluorine-18 has received significant attention in PET imaging research because of its appealing chemical and physical properties, including its small size, strong covalent bonds with carbon, nearly 100% positron efficiency, high molar activity, short half-life to reduce postprocedural radiation exposure, and low positron energy resulting in high imaging resolution. However, the development of novel ^18^F-labeled PET agents has been impeded by the harsh conditions that are typically required for the formation of the C–^18^F bond, necessitating protection of labile functional groups. Even though chelated aluminum-[^18^F]fluoride,^1,2^ silicon fluoride acceptors (SiFA),^3^ and [^18^F]trifluoroborates^4,5^ have been developed as alternative direct radiofluorination targets, the conventional [^18^F]fluoroalkyl and [^18^F]fluoroaryl motifs are still the predominant targets in PET radiochemistry because of their small size and high stability. To avoid the harsh C–^18^F bond formation conditions, small molecule PET agents are usually radiofluorinated in a protected form and deprotected thereafter. In particular, conjugation with ^18^F-labeled prosthetic groups is one of the most important strategies in constructing PET agents with structurally complicated and labile ligands.

Prosthetic groups are small organic molecules that contain both fluorine-18 and a highly reactive functional group that can be coupled to bioactive ligands efficiently. The ideal prosthetic group should present both high reactivity on its coupling site and high stability on other parts of the molecule, especially the C–^18^F bond. Prosthetic groups with [^18^F]fluoroaryl^6,7^ and [^18^F]fluoroalkyl motifs^8^ are common substructures in novel PET agents and can greatly affect the biodistribution properties of the bioactive ligands. The utility of ^18^F-labeled alkyl fluoride substructures has been demonstrated in many FDA-approved PET agents,^9^ as well as countless probes under investigation in biomedical studies. However, the stability of alkyl fluorides can be compromised in physiological environments by both substitution reactions with biological nucleophiles, as well as many enzymatic metabolic pathways.^10−14^ These facile *in vivo* defluorination processes are oftentimes responsible for high bone uptake in the subsequent PET imaging studies. Therefore, radiofluorinated arenes with higher *in vivo* stabilities are appealing substructures in the design of new PET agents.

To incorporate such appealing motifs, both electrophilic fluorination and nucleophilic aromatic substitution (S_N_Ar) radiofluorination methods have been developed, thereby offering a variety of choices for radiochemists.^15,16^ Electrophilic radiofluorinations can utilize organotin,^17,18^ organosilicon,^19−21^ organomercury,^22−25^ organoboron,^26^ and organogermanium precursors.^27^ Balz–Schiemann and Wallach reactions with diazonium precursors/intermediates^28−31^ and direct S_N_Ar with nitro,^32^ ammonium,^33^ halogen,^34^ sulfonium,^35−38^ and iodonium^39^ leaving groups have been applied for decades. Transition-metal-catalyzed reactions,^40−49^ oxidative fluorinations,^50,51^ C–H functionalization,^52−54^ and deoxyfluorination of phenols^55,56^ have also been reported, along with light-mediated radical radiofluorination method.^57−59^ These established methods supported the radiosynthesis of ^18^F-labeled aryl fluoride motifs and related PET agents. However, limitations of these methods are widely acknowledged either in the efficiency, feasibility, residue metal toxicity concerns, or functional group tolerance. As a result, multistep ^18^F-radiosynthesis is usually employed where the functional group constructions are carried out after radiofluorination of arenes. The prolonged total synthesis time reduces the efficiency because of rapid decay loss of fluorine-18 and hinders the implementation of radiofluorinated arene motifs in new PET agent development. Even though these conventional methods have demonstrated great value in PET radiochemistry and still are expected to maintain significant impact in promoting future tracer development, new radiofluorination methods with higher efficiency, step economy, and functional group tolerance will always benefit and promote the related biomedical researches.

Developed by the Nicewicz group, the organophotoredox-catalyzed cation radical-accelerated S_N_Ar reaction has demonstrated broad applications in the functionalization of arenes.^60−68^ Featuring mild conditions and highly reactive intermediates, this method allows for the efficient conversion of arenes while offering a high functional group tolerance. Initiated from a collaboration between the Nicewicz and Li groups, this aforementioned method has been applied to radiochemistry to afford direct C–H radiofluorination,^69,70^ (pseudo) halide interconversion radiofluorination,^71^ and deoxyfluorination,^72^ in addition to a series of radiocyanation reactions.^73,74^ Numerous small molecule substrates with ^18^F-labeled aryl fluoride motifs have been synthesized via these methods. Additionally, several PET imaging agents, including [^18^F]fenoprofen^69^ and [^18^F]F-DOPA,^71^ have also been prepared. However, the efficient synthesis of ^18^F-labeled prosthetic groups has not yet benefitted from these methods. Despite the robust reactivity in conjugations, many coupling functionalities in prosthetic groups are electron deficient, thereby making them inert under our photoredox conditions because of their high oxidation potential. Taking advantage of this, we envisioned the application of our organophotoredox-mediated method in a one-step radiosynthesis of prosthetic groups with robust functionalities.

**Figure 1:**
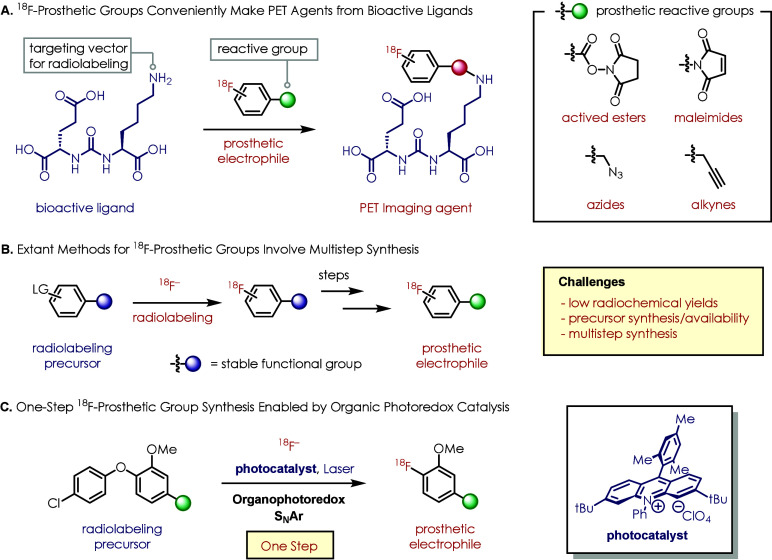
Functional-group-tolerant organophotoredox-catalyzed radiofluorination enables one-step radiosynthesis of prosthetic groups with [^18^F]fluoroaryl motif.

Herein, we report the utilization of organophotoredox-catalyzed deoxyradiofluorination in the efficient and step-economic preparation of prosthetic groups with an [^18^F]fluoroaryl motif, including a one-step metal-free preparation of the [^18^F]fluorobenzyl bromide analogue, the [^18^F]*N*-succinimidylfluorobenzoate analogue, and the first report of an ^18^F-labeled aryl fluoride isocyanate synthon, as well as the first report of a radiofluorinated isocyanide synthon (Figure 1). We also report the examples of PET agent constructions with some of these synthons and provide guidelines on the coupling procedures. The improved efficiency in the preparation of these synthons will promote the development of new PET agents, reduce cost, and facilitate the use of PET imaging for a range of applications. Our method provides an important alternative step-economic radiosynthetic route to structurally labile prosthetic group types that have been reported before, especially when metal-free conditions are desired. More importantly, because of the high functional group compatibility, our method can be advantageous in new prosthetic group development and new radiopharmaceutical development as it promotes late-stage radiofluorination of arenes, as demonstrated by the development of isocyanate and isocyanide synthons.

## Results and Discussion

**Figure 2:**
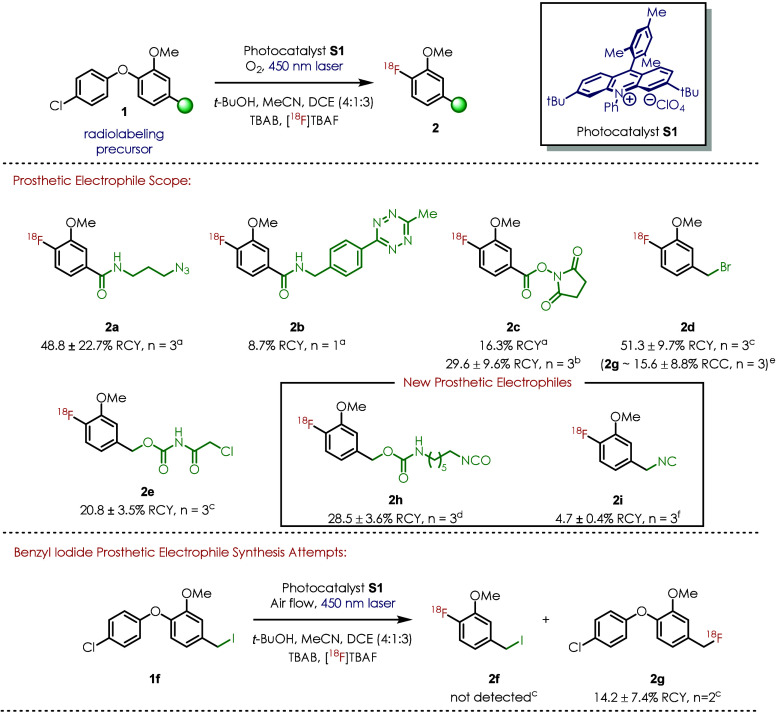
One-step radiosyntheses of prosthetic groups via organophotoredox-catalyzed S_N_Ar. ^a^Condition A: precursor **1** (0.05 mmol), TBAB/MeCN (15 μL, 60 mg/mL), oxygen flow, solvent mixture (800 μL), photocatalyst **S1** (5 mol %), laser irradiation (450 nm, 30 min). ^b^Condition B: precursor **1** (0.05 mmol), no TBAB/MeCN, air flow, solvent mixture (800 μL), photocatalyst **S1** (5 mol %), laser irradiation (450 nm, 20 min). ^c^Condition C: precursor **1** (0.05 mmol), TBAB/MeCN (25 μL, 60 mg/mL), air flow, solvent mixture (400 μL), photocatalyst **S1** (5 mol %), laser irradiation (450 nm, 20 min). ^d^Condition D: precursor **1** (0.05 mmol), no TBAB/MeCN, air flow, solvent mixture (400 μL), photocatalyst **S1** (5 mol %), laser irradiation (450 nm, 20 min). ^e^Estimated RCC calculated from isolated RCY of **2d** and radio-HPLC peak ratio between **2d** and **2g.**^f^Condition F: precursor **1** (0.016 mmol), no air flow, TBAB/MeCN (8.3 μL, 60 mg/mL), solvent mixture (133 μL), photocatalyst **S1** (5 mol %), sealed quartz tube, LED irradiation (450 nm, 20 min). ^g^All radiochemical yields (RCYs) reported in this figure are determined from an aliquot of the reaction crude and the collected activity after HPLC column and decay correction.

The efficiency and regioselectivity of our photoredox-mediated process can be affected by the electron density of the arene precursors. After preliminary studies, we determined that labeling precursors with an electron-donating group (such as a methoxy group) are the most promising precursors for our radiofluorination process, and we incorporated this motif into our labeling precursors. Azide alkyne cyclization and tetrazine *trans*-cyclooctene (TCO) ligation are two types of click chemistry that have been widely utilized in radiosynthesis. By applying the optimized conditions from our prior work,^72^ radiofluorinated azide **2a** was synthesized in excellent radiochemical yield (RCY) (48.8%) (Figure 2). Despite modest RCYs (8.7%), tetrazine **2b** was also prepared from this one-step radiosynthesis. It is noteworthy that only a few direct radiofluorination of tetrazine-containing compounds were reported previously.^75−78^

We then moved on to more challenging motifs containing functionalities that are appealing from a bioconjugation standpoint but too labile to survive conventional direct arene radiofluorination conditions. ^18^F-labeled *N*-succinimidylfluorobenzoate ([^18^F]SFB) is a robust esterification/amidation agent that has been coupled to protein and peptide probes.^79^ Radiofluorination of succinimidylbenzoate can be easily achieved by implementing silicon fluoride acceptor motif.^80,81^ However, the [^18^F]SFB synthons that do not have silicon fluoride acceptor substructures are much more challenging to synthesize. A three-step radiosynthesis is the typical procedure for preparing [^18^F]SFB via ^18^F-labeled fluorobenzaldehyde and benzoic acid intermediates.^79,82,83^ An alternative approach using an ester precursor via ^18^F-labeled benzoate ester and benzoic acid intermediates was also reported.^84^ The efficiency of this approach was improved by combining the first two steps into a one-pot reaction. A two-step synthesis of [^18^F]SFB was reported by Glaser et al. in 2008 with further improved step-economy via an oxidative esterification.^85^ Ritter et al. reported a one-step radiosynthesis of [^18^F]SFB from a transition metal complex precursor in 2012.^51^ Another one-step radiosynthesis of [^18^F]SFB was reported by Dammicco et al. using organotin precursor.^86^ A few one-step radiosyntheses of nicotinic acid *N*-succinimide ester ([^18^F]SFPy) were reported by Swenson et al. and Kuhnast et al. in 2020.^87,88^ However, there are no established methods to furnish [^18^F]SFB synthons in one step via a metal-free process. We employed the standard organophotoredox conditions previously outlined and successfully isolated methoxy [^18^F]SFB analogue **2c** in 16.3% RCY. We found that the tetrabutylammonium bicarbonate is a basic additive, which might have induced the decomposition of **2c** in the crude mixture. By removal of this additive from the reaction, the RCY of **2c** was improved to 29.6%. Good molar activity was observed for **2c** (2.42 Ci/μmol, or 89.6 GBq/μmol), while the reaction and isolation can be completed in under 40 min in total. As a comparison, most multistep radiosynthesis with automated synthetic modules can prepare [^18^F]SFB in a comparable or slightly higher RCY (25% to 46% RCY) with longer total synthesis time (40 to 98 min).^89−94^ Our step-economic method is especially appealing to researchers that do not have access to automated synthetic modules.

[^18^F]fluorobenzyl halides are another class of robust prosthetic groups that have been previously reported.^95^ Similar to [^18^F]SFB, the conventional approach to [^18^F]fluorobenzyl bromide ([^18^F]FBB) is a lengthy three-step radiosynthesis involving functional group modifications, such as reduction and bromination.^95−99^ Welch et al. reported a two-step radiosynthesis of [^18^F]fluorobenzyl iodide ([^18^F]FBI),^100^ and Ritter et al. reported the one-step synthesis of [^18^F]bromoethyl fluorobenzene synthon from a transition metal complex precursor.^50^ To the best of our knowledge, the typical three-step radiosynthesis is the only established method in the preparation of [^18^F]FBB. A one-step metal-free radiofluorination procedure in the preparation of these halide synthons would be very beneficial. Unfortunately, under our photoredox-catalyzed conditions, benzyl iodide precursor **1f** failed to generate [^18^F]fluoromethoxybenzyl iodide **2f**. Instead, [^18^F]benzyl fluoride derivative **2g** was isolated in a low RCY (14.2%). However, using benzyl bromide analogue **1d** as the precursor, we were able to isolate radiolabeled benzyl bromide synthon **2d** in 51.3% RCY along with small amount of **2g** [∼15.6% estimated radiochemical conversion (RCC)]. Furthermore, chlorinated synthon **2e** bearing a carbamate motif was synthesized in 20.8% RCY, thereby demonstrating the broad functional group tolerance of our method. These successful examples improved the efficiency and step economy for the preparation of radiofluorinated arene with halide coupling sites by providing a one-step metal-free approach permitted by the mild conditions of our organophotoredox-catalyzed S_N_Ar method.

After improving the efficiency in preparing the existing synthons with our method, we aimed to further utilize this method in the development of new reactive prosthetic groups. Isocyanate is a reactive motif that can undergo several types of transformations,^101^ including reaction with nucleophiles, such as amines, alcohols, and thiols. Reported isocyanate prosthetic groups and their applications in radiosyntheses include ^11^C-labeled isocyanate synthons,^102−105^ phenyl isocyanate/isothiocyanate with [^18^F]silicon fluoride acceptor motif,^106,107^ and phenyl isothiocyanate with [^18^F]fluoromethylphenyl motif.^108^ However, to the best of our knowledge, an isocyanate prosthetic group with a radiofluorinated arene motif is still undeveloped. We envisioned the potential implementation of an [^18^F]fluoroarene isocyanate prosthetic group in PET agent constructions and applied our photoredox-catalyzed method in the preparation of **2h**. The initial radiosynthesis of **2h** was carried out with the tetrabutylammonium bicarbonate (TBAB) additive, which did not yield any desired radio product. Considering the labile nature of the isocyanate motif, we removed the TBAB additive and successfully synthesized **2h** in 28.5% RCY.

Isocyanide is another type of robust functionality that is undeveloped in radiosynthesis. Besides the versatile transformations, such as multicomponent reactions and cyclization reactions with alkyne and alkenes, isocyanide is also a good ligand for transition metals and has been used in nuclear imaging and therapies when coordinated to radiometals. Radiofluorinated isocyanide synthons can potentially be used in complex PET agent constructions, as well as metal complexes, as imaging or theranostic agents. However, the vast potential of radiofluorinated isocyanide cannot be revealed unless a preparation procedure compatible with this functionality can be established. Our photoredox reaction successfully generated radiofluorinated isocyanide **2i**. Despite high radiochemical conversions, the majority of the radio product was carried away with the air flow, thereby leaving less than 15% radioactive material in the liquid crude mixture. We assume that the volatile radio material that evaporated was the desired isocyanide **2i**. However, it is difficult to confirm such a hypothesis since our initial attempts in trapping the volatile radio products failed. A revised procedure using a sealed quartz tube generated **2i** in 4.7% RCY. Even though the isocyanide **2i** was obtained in low overall efficiency, we have proven that such functionality is compatible with our photoredox condition.

**Figure 3:**
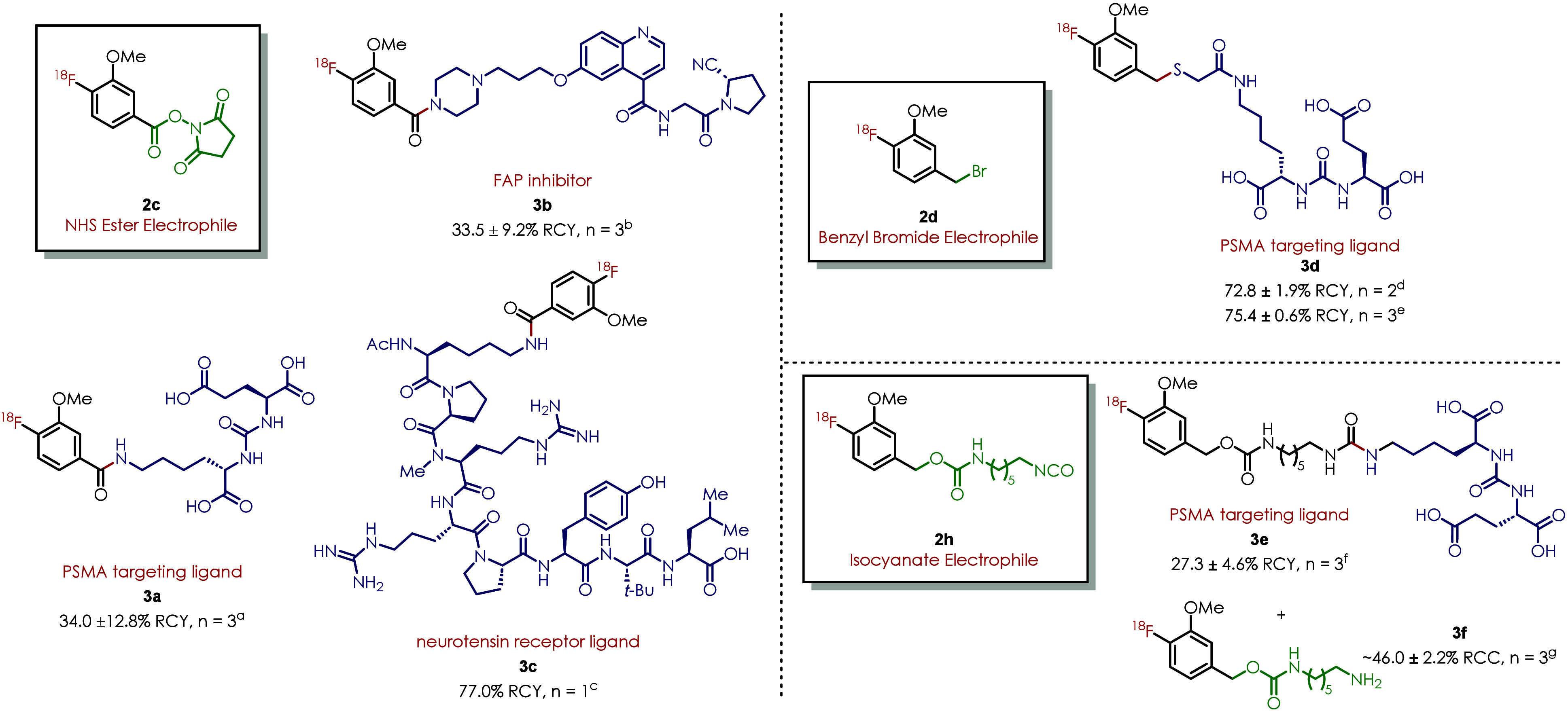
PET agent constructions utilizing [^18^F]SFB analogue **2c**, [^18^F]FBB analogue **2d**, and isocyanate synthon **2h**. ^a^PSMA, DIPEA, MeCN, and DMF, 60 °C, 30 min. ^b^FAPI, DIPEA, and MeCN, 60 °C, 30 min. ^c^NT20.3, DIPEA, MeCN, and water, 60 °C, 30 min. ^d^PSMA, MeCN, water, DMF, and Na_2_CO_3_, 70 °C, 30 min. ^e^PSMA, DIPEA, MeCN, and DMF, 70 °C, 30 min. ^f^PSMA, MeCN, water, and Na_2_CO_3_, 70 °C, 30 min. ^g^Estimated RCC of **3f** was calculated on the basis of the isolated RCY of **3e** and the radio-HPLC peak integration ratio between **3e** and **3f**. ^h^All radiochemical yields (RCYs) reported in this figure are determined from an aliquot of the reaction crude and the collected activity after HPLC column and decay correction. RCYs in this figure are the single-step coupling reaction RCYs.

To demonstrate the utility of the new prosthetic groups prepared by our method, three biomedical probes were coupled to methoxy [^18^F]SFB analogue **2c** to generate a series of new PET agents (Figure 3). Glu-NH–CO–NH-Lys is a small molecule probe that selectively binds to prostate-specific membrane antigen (PSMA) and has previously been applied to prostate cancer imaging.^109^ PSMA-targeting PET agent **3a** was prepared in 34.0% RCY from our methoxy [^18^F]SFB prosthetic group **2c**. Similarly, a fibroblast activation protein inhibitor (FAPI)^110^ and a peptide (NT20.3) that targets neurotensin receptor were also coupled to synthon **2c** to produce PET agents **3b** (33.5% RCY) and **3c** (77.0% RCY) respectively.

Our methoxy [^18^F]FBB analogue **2d** was also utilized in PET agent construction with a thiol derivative of PSMA targeting ligand. Because of reactivity competition concerns between water and the bioactive ligand in the conjugation reaction, PET agent **3d** was constructed under water-free conditions initially in 75.4% RCY. The future application of this prosthetic group might involve protein ligands, which would prefer water-containing conjugation conditions. Therefore, we also tested the conjugation of **2d** with the same PSMA targeting ligand in a water acetonitrile mixture solution and were pleased to find that **3d** was isolated in 72.8% RCY.

To demonstrate the utility of our isocyanate synthon **2h**, as well as provide an initial guidance on the coupling reaction conditions, PET agent **3e** with PSMA targeting vector was constructed (Figure 3). As a competing side reaction, the isocyanate motif is susceptible to the nucleophilic addition of water. The resulting decarboxylated amine **3f** was observed. The ratio of the desired PET agent **3e** and side product **3f** is highly pH-dependent in aqueous media. In the consideration of future PET agent constructions with proteins that cannot be performed in pure organic solvents, we established a coupling procedure in aqueous media and sodium carbonate buffer, which furnished the PET agent **3e** in 27.3% RCY. The hydrolyzed synthon was estimated to be produced in a higher RCC (46.0%).

The preparation of these new PET agents demonstrated the feasibility of applying our newly formed prosthetic groups. The coupling reactions and the subsequent HPLC isolation processes can be completed in under 50 min combined. To further explore the properties of these PET agents, PSMA targeting agent **3a** was applied in a small animal imaging study.

**Figure 4:**
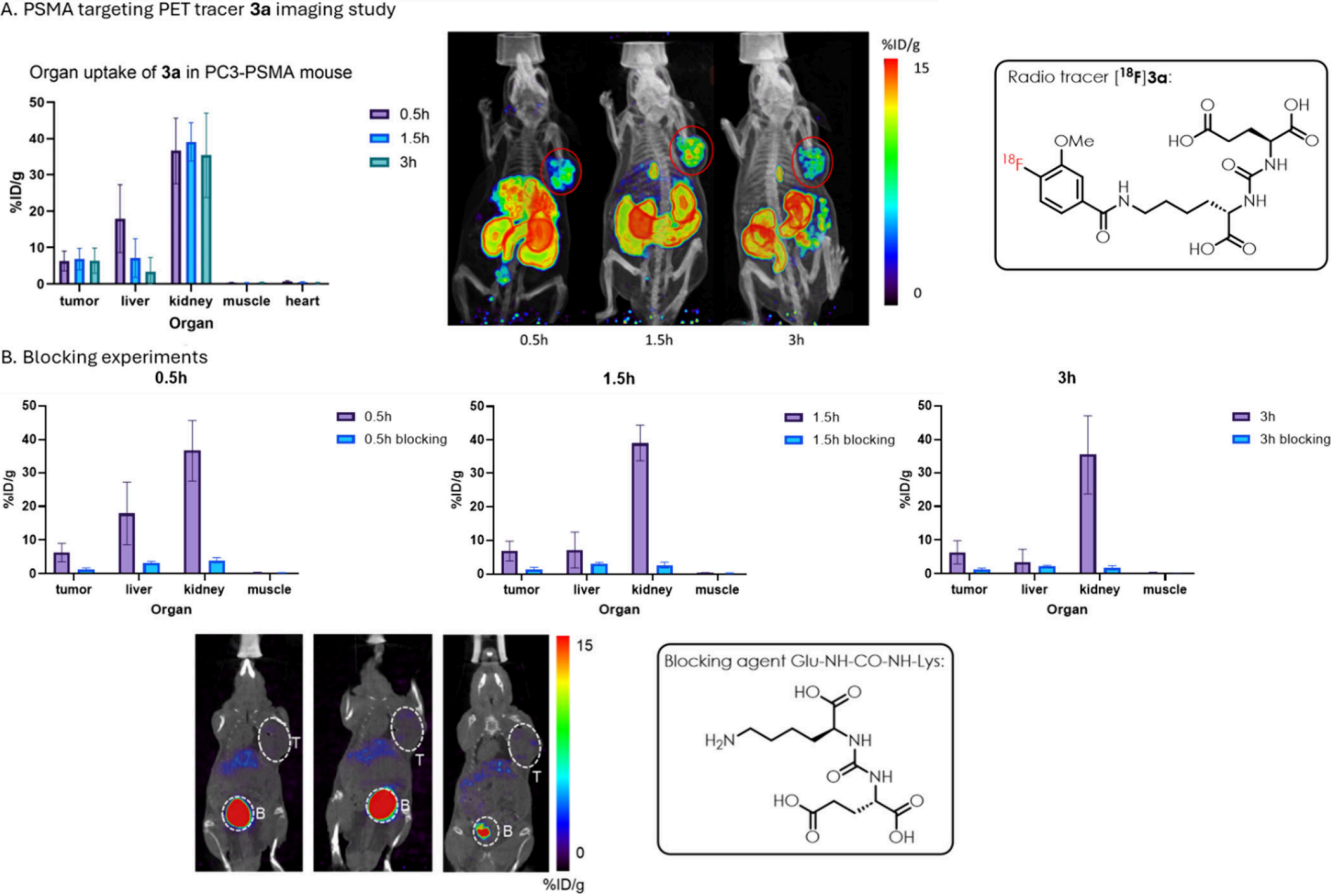
PET/CT imaging study of PC3-PSMA tumor-bearing mice with PET tracer **3a**. (A) Regular imaging study. Tumor circled in red. (B) PSMA-blocking experiment; representative PET/CT images of PSMA-blocked tumor-bearing mice at 0.5, 1.5, and 3 h postinjection; and organ uptakes with the comparison with normal [^**18**^**F**]**3a** tracer injection. T, tumor; B, bladder.

In PC3-PSMA tumor model imaging, tracer **3a** showed a good targeting efficiency and fast liver clearance (Figure 4). The tumor uptake is moderate and persistent, which is 6.31 ± 2.72, 6.84 ± 2.94, and 6.41 ± 3.45% ID/g at 0.5, 1.5, and 3 h postinjection (p.i.). It also showed fast liver clearance with liver uptake quickly decreasing from 17.92 ± 9.32% ID/g at 0.5 h to 3.41 ± 3.87% ID/g at 3 h. The tumor to liver ratio kept rising from 0.37 ± 0.05 at 0.5 h to 3.31 ± 2.26 at 3 h. However, it was observed that the kidney uptake did not decrease and maintained at the same level around 35% ID/g from 0.5 to 3 h, which might be due to the specific expression of PSMA in kidney. Very little of the radiotracer was in circulation after 0.5 h postinjection (blood values 0.73 ± 0.21% ID/g at 0.5 h, 0.44 ± 0.22% ID/g at 1.5 h and 0.28 ± 0.21% ID/g at 3 h). Overall, the *in vivo* image showed that the compound had been successfully labeled and has good targeting effects in tumor-bearing mouse models. Blocking experiments validated that the cancer uptake of **3a** is PSMA-specific. The blocking experiment was carried out by injecting 300 μg of Glu-NH–CO–NH-Lys to each tumor-bearing mouse 5 min before the injection of radio tracer [^**18**^**F**]**3a** (PSMA binding site is blocked by Glu-NH–CO–NH-Lys). The tumor uptake dropped from 6.31 ± 2.72, 6.84 ± 2.94, and 6.41 ± 3.45% ID/g to 1.41 ± 0.31, 1.40 ± 0.56, and 1.26 ± 0.43% ID/g at 0.5, 1.5, and 3 h postinjection (p.i.). Dramatic decrease of kidney uptake was also observed in the blocking group.

It is important to note that our method is not a replacement for established multistep methods, as some of these methods can achieve comparable overall RCYs with a little longer total synthesis time when automated synthesis modules are employed. Instead, our method is a promising alternative approach to prosthetic groups and is especially appealing to researchers without access to automated modules. The step economy and functional group compatibility of our method can be advantageous in new prosthetic group development. Our method also has advantages in application scenarios where metal-free conditions are desired.

## Conclusion

We utilized our organophotoredox methodology in the preparation of ^18^F-labeled prosthetic groups. Benefiting from the mild and robust reaction conditions, an efficient and functional-group-tolerant one-step radiosynthesis was established and can furnish radiolabeled prosthetic groups in under 40 min of total synthesis time. The furnished prosthetic groups have been applied in new PET agent constructions through couplings with molecular imaging probes. A PSMA-targeting PET agent showed decent rates of cancer uptake in the imaging experiment. With improved step economy, our method and the newly generated prosthetic groups will further promote the development of affordable PET agents.

## Supporting Information Available

General information on materials, methods, experimental details, analytical data (NMR spectra, high-resolution mass spectra), molar activity measurement, quality control and coinjection validation of radiolabeled products, and PET imaging data and blocking experiment data (PDF)
