# Supplementary material for: One-Step Synthesis of [18F]Aromatic Electrophile Prosthetic Groups via Organic Photoredox Catalysis
Source: ACS Cent Sci. 2024 Jul 18;10(8):1609–18. doi: 10.1021/acscentsci.4c00407 (PMC11363353; doi:10.1021/acscentsci.4c00407)
Supplement: Supplementary file 1 — oc4c00407_si_001.pdf [file oc4c00407_si_001.pdf]

Supplementary Information

One-Step Synthesis of [^{18}F]Aromatic Electrophile Prosthetic Groups via Organic Photoredox Catalysis

Manshu Li, [a] Carla Staton, [a] Xinrui Ma, [a] Weiling Zhao, [a] Liqin Pan, [a] Ben Giglio, [a] Haiden S. Berton, [a] Zhanhong Wu, [a] David A. Nicewicz*[b] and Zibo Li*[a]

[a] Department of Radiology, Biomedical Research Imaging Center, and Lineberger Comprehensive Cancer Center

University of North Carolina at Chapel Hill

Chapel Hill, North Carolina 27599 United States

E-mail: ziboli@med.unc.edu

[b] Department of Chemistry

University of North Carolina at Chapel Hill

Chapel Hill, North Carolina 27599 United States

E-mail: nicewicz@unc.edu

Contents

General information 3

General methods and materials 3

General radiochemistry methods and materials 3

General HPLC conditions 4

Preparation of precursors and non-radioactive analog references 5

Molar activity measurement 20

Radiochemistry 21

General photoredox method 21

Radiosyntheses 22

Small animal PET imaging study 45

Appendix: NMR 47

References 99

General information

General methods and materials

Commercially available chemicals and reagents were purchased from Fisher Scientific, Millipore Sigma, Acros, Alfa Aesar, TCI, Matrix Scientific, Combi-Blocks, Oakwood Chemical, Bide Pharm, and were used as received unless otherwise noted.

Diethyl ether, dichloromethane, tetrahydrofuran, toluene, acetonitrile, and dimethylformamide were dried by passing through activated alumina under nitrogen prior to use.

Column chromatography purifications were performed using technical grade silica gel (60Å, 230-400 mesh, 40-63 µm particle size) from Millipore Sigma, or using SiliaFlash P60 silica gel (40-63 µm) from Silicycle.

All catalyst and substrate syntheses were run under a nitrogen atmosphere unless otherwise noted.

Nuclear magnetic resonance (NMR) spectra were obtained using Varian 400 MR spectrometer (^{1}H NMR at 400 MHz, ^{13}C NMR at 100 MHz, ^{19}F NMR at 376 MHz). Some NMR spectra were obtained using Bruker Neo Console 500 MHz NMR spectrometer with a cryoprobe (^{1}H NMR at 500 MHz, ^{13}C NMR at 125 MHz, ^{19}F NMR at 470 MHz). All spectra were reported as parts per million. ^{1}H NMR and ^{13}C NMR spectra were referenced to the residual protium solvent peaks of chloroform-d (^{1}H NMR: 7.26 ppm, ^{13}C NMR: 77.16 ppm), or dimethyl sulfoxide-d_6 (^{1}H NMR: 2.50 ppm, ^{13}C NMR: 39.52 ppm).

High resolution mass spectra (HRMS) data were obtained via direct infusion using a ThermoScientific Q Exactive™ HF-X mass spectrometer with positive mode electrospray ionization, positive mode atmospheric-pressure chemical ionization, and/or atmospheric-pressure photoionization. Some HRMS data were obtained via direct infusion using a Thermo LTQ FT mass spectrometer with positive mode electrospray ionization.

General radiochemistry methods and materials

[^{18}F]Fluoride was produced via the ^{18}O (p, n) ^{18}F reaction by proton irradiation (40 µA, 45 min) of an [^{18}O]H_2O containing target in a GE PETTrace cyclotron. The aqueous solution of [^{18}F]Fluoride was delivered into a hot cell, and passed through a QMA cartridge (water preconditioning). The [^{18}F]Fluoride was then eluted from the cartridge with a solution mixture containing tetrabutylammonium bicarbonate aqueous solution (20%, w/w, 70 µL), water (53 µL), and acetonitrile (477 µL). After azeotropic drying with acetonitrile (1 mL X3) under stream of Argon

at 95 °C, the residue was dissolved in acetonitrile (1 mL) to afford the TBA[^{18}F]/MeCN solution for radiolabeling reactions.

The activities of fluorine-18 samples were measured by CRC-25 PET detector from Capintec, or by Atomlab 400 dose calibrator from Biodex.

After each reaction, an aliquot of reaction mixture was taken for the HPLC analysis/purification. The activity injected for HPLC was measured (α) and the time of injection was recorded. The fraction corresponding to the radiolabeled product was collected, the activity of the collected fraction was measured (β) and the time was recorded. The decay corrected β was calculated from the recorded isolation time. The decay corrected RCY was calculated by dividing decay corrected β by α.

Quality controls (QC) were done for most samples in order to confirm the purity of each isolated product: an aliquot of the fraction collected from radio-HPLC (containing the ^{18}F-labeled product) was injected for a separate HPLC analysis with both radio detector and UV detector.

Co-injections were done for most samples in order to confirm the identity of each isolated product: an aliquot of the fraction collected from radio-HPLC (containing the ^{18}F-labeled product) was mixed with ^{19}F-containing standard, then injected for a separate HPLC analysis with both radio detector and UV detector. For the samples without co-injections, the identities of the radiolabeled compounds were confirmed by comparison to authentic fluorine-19 standards.

General HPLC conditions

Reversed-phase high performance liquid chromatography (HPLC) was performed on an Agilent chromatography system (Model 1260 Infinity), or on a SHIMADZU chromatography system (Model CBM-20A).

HPLC column 1: Gemini® C18 10µ 110Å 250X4.6mm column

HPLC column 2: Luna® 5µ C18(2) 100Å 250X4.6mm column

HPLC gradient elution method A: using solvent A (0.1% TFA water) and solvent B (0.1% TFA acetonitrile). Flow rate: 1 mL/min. Grad/isocrat: 0 to 2 min: isocratic elution at 40% solvent B; 2 to 12 min: 40% to 95% solvent B; after 12 min: isocratic elution at 95% solvent B.

HPLC gradient elution method B: using solvent A (0.1% TFA water) and solvent B (0.1% TFA acetonitrile). Flow rate: 1 mL/min. Grad/isocrat: 0 to 2 min: isocratic elution at 5% solvent B; 2 to 22 min: 5% to 95% solvent B; after 22 min: isocratic elution at 95% solvent B.

HPLC gradient elution method C: using solvent A (0.1% TFA water) and solvent B (0.1% TFA acetonitrile). Flow rate: 1 mL/min. Grad/isocrat: 0 to 2 min: isocratic elution at 20% solvent B; 2 to 22 min: 20% to 60% solvent B; after 22 min: isocratic elution at 60% solvent B.

Preparation of precursors and non-radioactive analog references

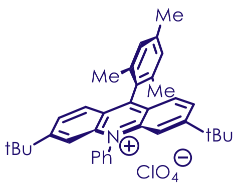

S1

9-mesityl-3,6-di-*tert*-butyl-10-phenylacridinium perchlorate (**S1**)

The acridinium photocatalyst **S1** was prepared according to a published procedure; spectral data are in agreement with literature values.[1]

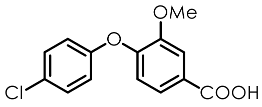

S2

4-(4-chlorophenoxy)-3-methoxybenzoic acid (**S2**)

Carboxylic acid **S2** was prepared according to a published procedure; spectral data are in agreement with literature values.[2]

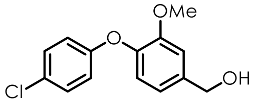

S3

(4-(4-chlorophenoxy)-3-methoxyphenyl)methanol (**S3**)

Alcohol **S3** was prepared according to a published procedure; spectral data are in agreement with literature values.[2]

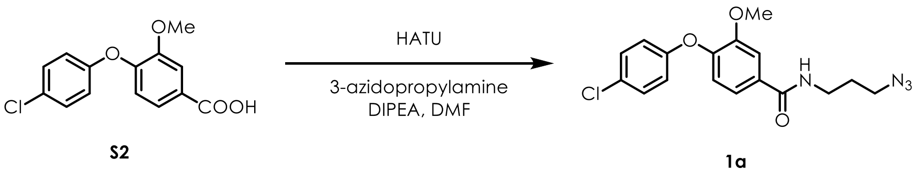

N-(3-azidopropyl)-4-(4-chlorophenoxy)-3-methoxybenzamide (**1a**)

To a solution of **S2** (14 mg, 0.050 mmol) in DMF (0.2 mL) was added HATU (38 mg, 0.10 mmol) and DIPEA (43.5 µL, 0.25 mmol). After 1 hour, 3-azidopropylamine (4.9 µL, 0.050 mmol) was

added. After 3 days, flash chromatography (33% EtOAc in hexane) afforded **1a** (6.8 mg, 38% yield) as a milky wax: ^{1}H NMR (400 MHz, $CDCl_3$) δ 7.54 (d, *J* = 1.7 Hz, 1H), 7.27 (d, *J* = 9.2 Hz, 2H), 7.20 (dd, *J* = 8.2, 1.9 Hz, 1H), 6.95-6.86 (m, 3H), 6.36 (br s, 1 H), 3.89 (s, 3H), 3.56 (dt, apparent q, *J* = 6.4 Hz, 2H), 3.46 (t, *J* = 6.4 Hz, 2H), 1.92 (tt, apparent quintet, *J* = 6.5 Hz, 2H); ^{13}C NMR (100 MHz, $CDCl_3$) δ 167.0, 155.8, 151.3, 148.1, 131.2, 129.8, 128.5, 119.7, 119.3, 119.0, 112.4, 56.3, 49.8, 38.1, 28.9; HRMS (ESI) m/z $[M + H]^+$ calcd for $C_{17}H_{18}ClN_4O_3$ 361.1067; found 361.1062.

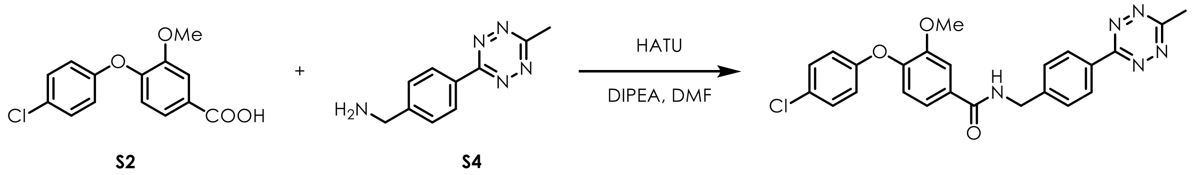

4-(4-chlorophenoxy)-3-methoxy-N-(4-(6-methyl-1,2,4,5-tetrazin-3-yl)benzyl)benzamide (**1b**)

To a solution of **S2** (14 mg, 0.050 mmol) in DMF (0.2 mL) was added HATU (38 mg, 0.10 mmol) and DIPEA (43.5 µL, 0.25 mmol). After 1 hour, amine **S4** (10 mg, 0.050 mmol) was added. After 2 days, gradient flash chromatography (9% EtOAc in hexane to 33% EtOAc in hexane) afforded **1b** (12.2 mg, 53% yield) as a purple powder: ^{1}H NMR (400 MHz, $CDCl_3$) δ 8.54 (d, *J* = 8.2 Hz, 2H), 7.60 (d, *J* = 1.4 Hz, 1H), 7.54 (d, *J* = 8.2 Hz, 2H), 7.31-7.23 (m, 3H), 6.93-6.85 (m, 3H), 6.72 (t, *J* = 5.8 Hz, 1H), 4.75 (d, *J* = 5.7 Hz, 2H), 3.88 (s, 3H), 3.08 (s, 3H); ^{13}C NMR (100 MHz, $CDCl_3$) δ 167.3, 166.8, 163.8, 155.5, 151.2, 148.2, 143.1, 131.1, 130.6, 129.7, 128.5, 128.4, 128.3, 119.5, 119.2, 119.0, 112.4, 56.1, 43.8, 21.2; HRMS (ESI) m/z $[M + H]^+$ calcd for $C_{24}H_{21}ClN_5O_3$ 462.1333; found 462.1327.

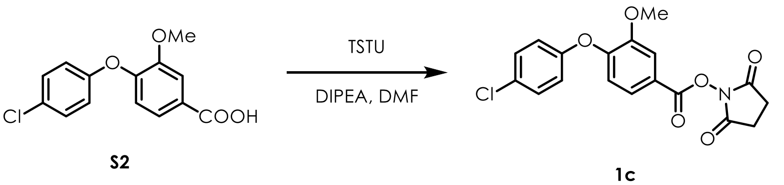

2,5-dioxopyrrolidin-1-yl 4-(4-chlorophenoxy)-3-methoxybenzoate (**1c**)

To a solution of carboxylic acid **S2** (249 mg, 0.89 mmol) in DMF (3.0 mL) was added *N*,*N*,*N'*,*N'*-tetramethyl-*O*-(*N*-succinimidyl)uronium tetrafluoroborate (TSTU, 536 mg, 1.78 mmol) and DIPEA (472 µL, 2.70 mmol). After 20 hours, flash chromatography (33% EtOAc in hexane) furnished the succinimidyl ester **1c** (250 mg, 74% yield) as a white solid: ^{1}H NMR (400 MHz, $CDCl_3$) δ 7.76-7.68 (m, 2H), 7.33 (d, *J* = 8.9 Hz, 2H), 6.96 (d, *J* = 8.9 Hz, 2H), 6.91 (d, *J* = 8.4 Hz, 1H), 3.93 (s, 3H), 2.91 (s, 4H); ^{13}C NMR (100 MHz, $CDCl_3$) δ 169.2, 161.2, 154.5, 151.7, 150.6, 130.0, 129.4, 124.6, 120.5, 120.3, 118.4, 114.1, 56.2, 25.7; HRMS (APCI) m/z $[M]^{+}$ calcd for $C_{18}H_{14}ClO_6N$ 375.0510; found 375.0507.

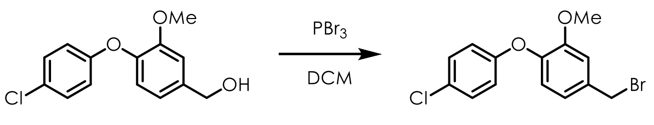

S3 **1d**

4-(bromomethyl)-1-(4-chlorophenoxy)-2-methoxybenzene (**1d**).

To a solution of alcohol **S3** (380 mg, 1.44 mmol) in DCM (14.4 mL) was added phosphorus tribromide solution (2.16 mL, 1 M solution in DCM, 2.16 mmol). After 6 hours, water (1 mL) was added. The mixture was extracted with EtOAc. Concentration and flash chromatography (9% EtOAc in hexane) furnished brominated **1d** (363 mg, 77% yield) as a pale yellow oil: ^{1}H NMR (400 MHz, $CDCl_3$) δ 7.27-7.22 (m, 2H), 7.03 (d, *J* = 1.7 Hz, 1H), 6.97-6.85 (m, 4H), 4.50 (s, 2H), 3.84 (s, 3H); ^{13}C NMR (100 MHz, $CDCl_3$) δ 156.3, 151.4, 145.1, 134.8, 129.7, 127.9, 121.9, 120.9, 118.8, 113.7, 56.2, 33.6; HRMS (ESI) m/z $[M + H]^+$ calcd for $C_{14}H_{13}BrClO_2$ 326.9787; found 326.9779.

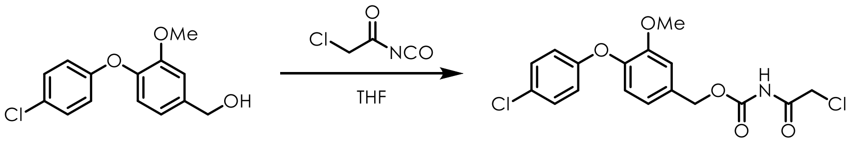

S3 **1e**

4-(4-chlorophenoxy)-3-methoxybenzyl (2-chloroacetyl)carbamate (**1e**).

To a solution of alcohol **S3** (300 mg, 1.13 mmol) in THF (11.3 mL) was added chloroacetyl isocyanate (96 µL, 1.13 mmol). After 4 hours, mixture was concentrated. Gradient flash chromatography (9% EtOAc in hexane to 33% EtOAc in hexane) afforded **1e** (202 mg, 46% yield) as a pale yellow solid: ^{1}H NMR (400 MHz, DMSO-d6) δ 11.06 (s, 1H), 7.35 (d, *J* = 8.9 Hz, 2H), 7.25 (s, 1H), 7.12-7.01 (m, 2H), 6.83 (d, *J* = 8.9 Hz, 2H), 5.16 (s, 2H), 4.50 (s, 2H), 3.74 (s, 3H); ^{13}C NMR (100 MHz, DMSO-d6) δ 166.7, 156.7, 151.4, 151.2, 143.0, 133.4, 129.5, 125.9, 121.8, 121.0, 117.6, 113.5, 66.4, 55.8, 44.2; HRMS (ESI) m/z $[M + Na]^+$ calcd for $C_{17}H_{15}Cl_2NO_5Na$ 406.0225; found 406.0226.

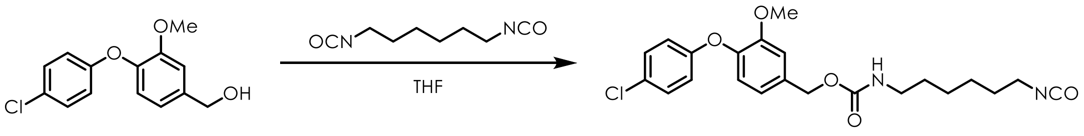

S3 **1h**

4-(4-chlorophenoxy)-3-methoxybenzyl (6-isocyanatohexyl)carbamate (**1h**)

To a solution of alcohol **S3** (400 mg, 1.51 mmol) in MeCN (1.5 mL) was added hexamethylene diisocyanate (1.21 mL, 7.55 mmol). After 7 days, mixture was concentrated. Flash chromatography (33% EtOAc in hexane) furnished the isocyanate **1h** (351 mg, 54% yield) as a pale-yellow oil: ^{1}H NMR (400 MHz, $CDCl_3$) δ 7.20 (d, *J* = 8.9 Hz, 2H), 6.98 (s, 1H), 6.90 (s, 2H), 6.83 (d, *J* = 8.9 Hz, 2H), 5.05 (s, 2H), 4.79 (br s, 1H), 3.79 (s, 3H), 3.26 (t, *J* = 6.6 Hz, 2H), 3.18 (dt,

apparent q, J = 6.6 Hz, 2H), 1.63-1.44 (m, 4H), 1.42-1.27 (m, 4H); ^{13}C NMR (100 MHz, $CDCl_3$) δ 156.6, 156.4, 151.4, 144.5, 133.9, 129.5 (2C), 127.6, 121.0, 118.4 (2C), 112.9, 66.4, 56.0, 42.9, 41.0, 31.2, 30.0, 26.3, 26.2; HRMS (ESI) m/z $[M + H]^+$ calcd for $C_{22}H_{26}ClN_2O_5$ 433.1530; found 433.1523.

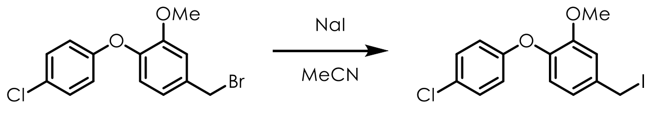

1d **1f**

1-(4-chlorophenoxy)-4-(iodomethyl)-2-methoxybenzene (**1f**)

To a solution of bromide **1d** (296 mg, 0.90 mmol) in MeCN (18 mL) was added sodium iodide (405 mg, 2.7 mmol). After 24 hours, concentration and flash chromatography (5% EtOAc in hexane) furnished the iodide **1f** (328 mg, 97% yield) as a yellow oil: ^{1}H NMR (400 MHz, $CDCl_3$) δ 7.26 (s, 1H), 7.24 (s, 1H), 7.00 (d, J = 1.8 Hz, 1H), 6.94 (dd, J = 8.2, 1.9 Hz, 1H), 6.90-6.82 (m, 3H), 4.47 (s, 2H), 3.84 (s, 3H); ^{13}C NMR (100 MHz, $CDCl_3$) δ 156.3, 151.4, 144.6, 136.3, 129.7, 127.9, 121.5, 120.9, 118.8, 113.4, 56.1, 5.7; HRMS (ESI) m/z $[M + H]^+$ calcd for $C_{14}H_{13}ClIO_2$ 374.9649; found 374.9643.

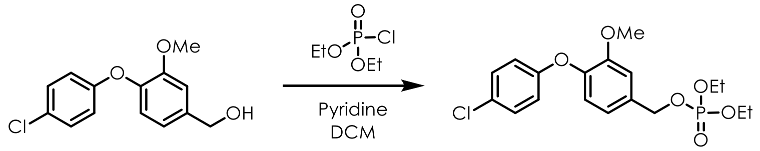

S3 **S5**

4-(4-chlorophenoxy)-3-methoxybenzyl diethyl phosphate (**S5**)

To a solution of alcohol **S3** (771 mg, 2.91 mmol) in DCM (5.8 mL) was added diethyl chlorophosphate (0.84 mL, 5.8 mmol) and pyridine (0.59 mL, 7.3 mmol). After 3.5 hours, water (10 mL) was added. The mixture was extracted with DCM. Concentration and flash chromatography (50% EtOAc in hexane) afforded **S5** (978 mg, 84% yield) as a yellow oil: ^{1}H NMR (400 MHz, $CDCl_3$) δ 7.24 (d, J = 8.9 Hz, 2H), 7.06 (s, 1H), 6.94 (s, 2H), 6.86 (d, J = 8.9 Hz, 2H), 5.04 (d, J = 8.2 Hz, 2H), 4.12 (dt, J = 14.4, 7.3 Hz, 4H), 3.84 (s, 3H), 1.33 (t, J = 7.0 Hz, 6 H); ^{13}C NMR (100 MHz, $CDCl_3$) δ 156.5, 151.5, 145.0, 133.4 (d, J = 7.0 Hz), 129.6, 127.8, 121.0, 120.8, 118.6, 112.7, 68.9 (d, J = 5.5 Hz), 64.0 (d, J = 5.9 Hz), 56.2, 16.3 (d, J = 6.7 Hz); HRMS (ESI) m/z $[M + Na]^+$ calcd for $C_{18}H_{22}ClO_6PNa$ 423.0740, found 423.0727.

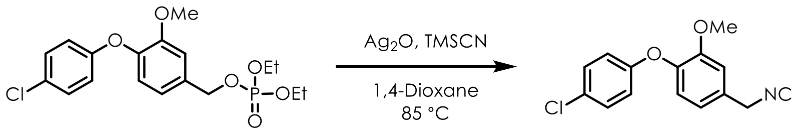

S5 **1i**

1-(4-chlorophenoxy)-4-(isocyanomethyl)-2-methoxybenzene (**1i**)

To a solution of **S5** (200 mg, 0.50 mmol) in 1,4-dioxane (1 mL) was added silver oxide (11.6 mg, 0.05 mmol) and TMSCN (125 µL, 1.0 mmol). The mixture was heated at 85 °C for 30 min. Flash chromatography (25% EtOAc in hexane) afforded **1i** (54.3 mg, 40% yield) as a pale-yellow oil: ^{1}H NMR (400 MHz, $CDCl_3$) δ 7.25 (d, *J* = 8.9 Hz, 2H), 7.00-6.95 (m, 2H), 6.91-6.83 (m, 3H), 4.63 (s, 2H), 3.85 (s, 3H); ^{13}C NMR (100 MHz, $CDCl_3$) δ 158.1 (t, *J* = 5.3 Hz), 156.4, 151.9, 145.0, 129.7, 129.4, 127.9, 121.4, 119.5, 118.6, 111.4, 56.2, 45.4 (t, *J* = 7.1 Hz); HRMS (APCI/APPI) m/z $[M]^+$ calcd for $C_{15}H_{12}ClNO_2$ 273.0557, found 273.0549.

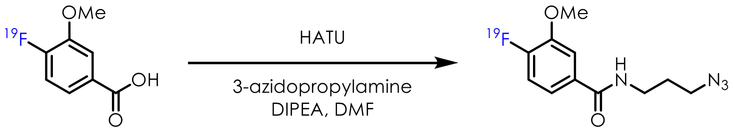

S6 [^{19}**F**]**2a**

N-(3-azidopropyl)-4-fluoro-3-methoxybenzamide ([^{19}**F**]**2a**)

To a solution of carboxylic acid **S6** (8.5 mg, 0.050 mmol) in DMF (0.2 mL) was added HATU (38 mg, 0.10 mmol) and DIPEA (43.5 µL, 0.25 mmol). After 1 hour, 3-azidopropylamine (4.9 µL, 0.050 mmol) was added. After 3 days, flash chromatography (33% EtOAc in hexane) afforded [^{19}**F**]**2a** (6.6 mg, 52% yield) as a colorless oil: ^{1}H NMR (400 MHz, $CDCl_3$) δ 7.50 (dd, *J* = 8.1, 1.7 Hz, 1H), 7.24-7.17 (m, 1H), 7.08 (dd, *J* = 10.6, 8.4 Hz, 1H), 6.50 (br s, 1H), 3.92 (s, 3H), 3.54 (dd, *J* = 12.3, 6.3 Hz, 2H), 3.44 (t, *J* = 6.5 Hz, 2H), 1.90 (tt, apparent quintet, *J* = 6.5 Hz, 2H); ^{19}F NMR (376 MHz, $CDCl_3$) δ -130.0 - -130.1 (m); ^{13}C NMR (100 MHz, $CDCl_3$) δ 166.8, 154.4 (d, *J* = 254.5 Hz), 147.9 (d, *J* = 11.0 Hz), 130.9 (d, *J* = 3.6 Hz), 118.8 (d, *J* = 7.6 Hz), 115.8 (d, *J* = 19.3 Hz), 113.0 (d, *J* = 2.8 Hz), 56.3, 49.6, 38.0, 28.7; HRMS (ESI) m/z $[M + H]^+$ calcd for $C_{11}H_{14}FN_4O_2$ 253.1101; found 253.1095.

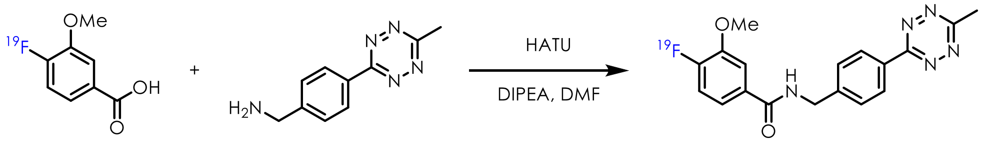

S6 **S4** [^{19}**F**]**2b**

4-fluoro-3-methoxy-N-(4-(6-methyl-1,2,4,5-tetrazin-3-yl)benzyl)benzamide ([^{19}**F**]**2b**)

To a solution of carboxylic acid **S6** (8.5 mg, 0.050 mmol) in DMF (0.2 mL) was added HATU (38 mg, 0.10 mmol) and DIPEA (43.5 µL, 0.25 mmol). After 1 hour, amine **S4** (10 mg, 0.05 mmol) was added. After 18 hours, flash chromatography (33% EtOAc in hexane) afforded [^{19}**F**]**2b** (13.5 mg, 77% yield) as a purple solid: ^{1}H NMR (400 MHz, $CDCl_3$) δ 8.56 (d, *J* = 8.2 Hz, 2H), 7.60-7.53 (m, 3H), 7.26 (s, 1H), 7.10 (dd, *J* = 10.5, 8.5 Hz, 1H), 6.60 (br s, 1H), 4.75 (d, *J* = 5.7 Hz, 2H), 3.94 (s, 3H), 3.09 (s, 3H); ^{19}F NMR (376 MHz, $CDCl_3$) δ -129.5 - -129.7 (m); ^{13}C NMR (100 MHz, $CDCl_3$) δ 167.4, 166.7, 164.0, 154.7 (d, *J* = 252.3 Hz), 148.2 (d, *J* = 10.8 Hz), 143.1, 131.3, 130.8 (d, *J* = 3.7 Hz), 128.6, 128.5, 119.0 (d, *J* = 7.6 Hz), 116.0 (d, *J* = 19.2 Hz), 113.3 (d, *J* = 2.9 Hz), 56.5, 44.0, 21.3; HRMS (ESI) m/z $[M + H]^+$ calcd for $C_{18}H_{17}FN_5O_2$ 354.1366; found 354.1360.

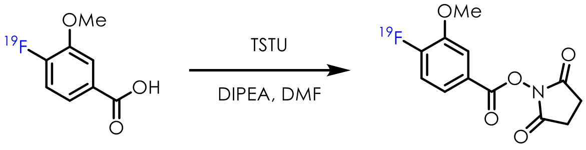

S6 [^{19}**F**]**2c**

2,5-dioxopyrrolidin-1-yl 4-fluoro-3-methoxybenzoate ([^{19}**F**]**2c**)

To a solution of carboxylic acid **S6** (50 mg, 0.29 mmol) in DMF (970 µL) was added *N*,*N*,*N'*,*N'*-tetramethyl-*O*-(*N*-succinimidyl)uronium tetrafluoroborate (174 mg, 0.58 mmol) and DIPEA (152 µL, 0.87 mmol). After 19 hours, flash chromatography (33% EtOAc in hexane) furnished succinimidyl ester [^{19}**F**]**2c** (71 mg, 91% yield) as a white solid: ^{1}H NMR (400 MHz, $CDCl_3$) δ 7.80-7.74 (m, 1H), 7.70 (dd, *J* = 8.1, 1.8 Hz, 1H), 7.20 (dd, *J* = 10.5, 8.5 Hz, 1H), 3.95 (s, 3H), 2.91 (s, 4H); ^{19}F NMR (376 MHz, $CDCl_3$) δ -123.1 - -123.2 (m); ^{13}C NMR (100 MHz, $CDCl_3$) δ 169.3, 161.1, 156.8 (d, *J* = 257.4 Hz), 148.2, (d, *J* = 11.2 Hz), 124.6 (d, *J* = 8.4 Hz), 121.5 (d, *J* = 3.6 Hz), 116.7 (d, *J* = 19.5 Hz), 115.2 (d, *J* = 3.7 Hz), 56.5, 25.8; HRMS (ESI) m/z $[M + H]^+$ calcd for $C_{12}H_{11}FNO_5$ 268.0621; found 268.0616.

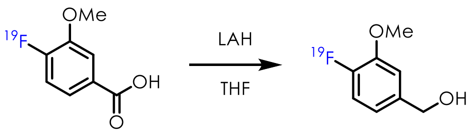

S6 **S7**

(4-fluoro-3-methoxyphenyl)methanol (**S7**).

To a solution of carboxylic acid **S6** (500 mg, 2.94 mmol) in THF (2.9 mL) was added lithium aluminum hydride powder (223 mg, 5.88 mmol). After 20 minutes, water (10 mL) was added slowly. The mixture was extracted with EtOAc. The organic layer was concentrated. Gradient flash chromatography (9% EtOAc in hexane to 33% EtOAc in hexane) afforded **S7** (463 mg, quantitative yield) as a pale yellow liquid: ^{1}H NMR (400 MHz, $CDCl_3$) δ 7.08-6.98 (m, 2H), 6.89-6.82 (m, 1H), 4.64 (s, 2H), 3.90 (s, 3H); ^{19}F NMR (376 MHz, $CDCl_3$) δ -136.9 - -137.0 (m); ^{13}C NMR (100 MHz, $CDCl_3$) δ 152.0 (d, *J* = 245.3 Hz), 147.9 (d, *J* = 10.7 Hz), 137.3 (d, *J* = 3.8 Hz), 119.3 (d, *J* = 7.0 Hz), 116.0 (d, *J* = 18.5 Hz), 112.3 (d, *J* = 2.0 Hz), 65.0, 56.3; HRMS (APCI/APPI) m/z $[M]^{+}$ calcd for $C_8H_9FO_2$ 156.0587; found 156.0580.

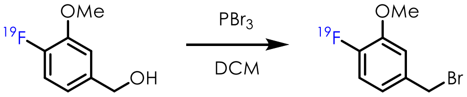

S7 [^{19}**F**]**2d**

4-(bromomethyl)-1-fluoro-2-methoxybenzene ([^{19}**F**]**2d**).

To a solution of alcohol **S7** (20 mg, 0.13 mmol) in DCM (1.3 mL) was added phosphorus tribromide solution (0.20 mL, 1 M solution in DCM, 0.20 mmol). After 1 hour, water (1 mL) was added. The mixture was extracted with EtOAc. The organic layer was concentrated. Flash

chromatography (33% EtOAc in hexane) afforded brominated [^{19}**F**]**2d** (13.7 mg, 49% yield) as a pale yellow oil: ^{1}H NMR (400 MHz, $CDCl_3$) δ 7.06-6.97 (m, 2H), 6.94-6.89 (m, 1H), 4.46 (s, 2H), 3.90(s, 3H); ^{19}F NMR (375 MHz, $CDCl_3$) δ -137.75 - -134.84 (m); ^{13}C NMR (100 MHz, $CDCl_3$) δ 152.5 (d, *J* = 257.8 Hz), 147.9 (d, *J* = 10.9 Hz), 134.2 (d, *J* = 3.9 Hz), 121.6 (d, *J* = 7.3 Hz), 116.28 (d, *J* = 18.6 Hz), 114.3 (d, *J* = 2.2 Hz), 56.4, 33.3; HRMS (APCI/APPI) m/z $[M]^{+}$ calcd for C_8H_8BrFO 217.9743; found 217.9736.

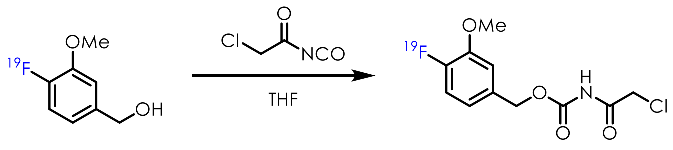

S7 [^{19}**F**]**2e**

4-fluoro-3-methoxybenzyl (2-chloroacetyl)carbamate ([^{19}**F**]**2e**).

To a solution of alcohol **S7** (20 mg, 0.13 mmol) in THF (1.3 mL) was added chloroacetyl isocyanate (17 µL, 0.20 mmol). After 19 hours, the mixture was concentrated. Flash chromatography (33% EtOAc in hexane) afforded [^{19}**F**]**2e** (23.2 mg, 66% yield) as a white solid: ^{1}H NMR (400 MHz, $CDCl_3$) δ 8.02 (s, 1H), 7.07 (dd, *J* = 11.0, 8.2 Hz, 1H), 6.99 (dd, *J* = 8.0, 1.7 Hz, 1H), 6.94-6.88 (m, 1H), 5.15 (s, 2H), 4.46 (s, 2H), 3.90 (s, 3H); ^{19}F NMR (375 MHz, $CDCl_3$) δ -134.3 - -134.2 (m); ^{13}C NMR (100 MHz, $CDCl_3$) δ 166.5, 152.8 (d, *J* = 248.2 Hz), 151.0, 148.0 (d, *J* = 10.7 Hz), 130.9 (d, *J* = 3.9 Hz), 121.5 (d, *J* = 7.2 Hz), 116.4 (d, *J* = 18.7 Hz), 114.1 (d, *J* = 2.3 Hz), 68.2, 56.4, 43.6; HRMS (ESI) m/z $[M + Na]^{+}$ calcd for $C_{11}H_{11}ClFNO_4Na$ 298.0258; found 298.0253.

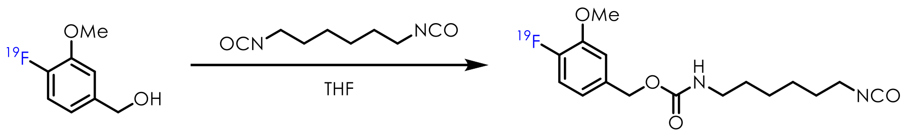

S7 [^{19}**F**]**2h**

4-fluoro-3-methoxybenzyl (6-isocyanatohexyl)carbamate ([^{19}**F**]**2h**)

To a solution of alcohol **S7** (20 mg, 0.13 mmol) in THF (152 µL) was added hexamethylene diisocyanate (122 µL, 0.76 mmol). After 7 days, the mixture was concentrated. Gradient flash chromatography (9% EtOAc in hexane to 33% EtOAc in hexane) furnished isocyanate [^{19}**F**]**2h** (12 mg, 29% yield) as pale-yellow oil: ^{1}H NMR (400 MHz, $CDCl_3$) δ 7.04 (dd, *J* = 11.1, 8.3 Hz, 1H), 6.97 (dd, *J* = 8.1, 1.4 Hz, 1H), 6.92-6.85 (m, 1H), 5.03 (s, 2H), 4.73 (br s, 1H), 3.89 (s, 3H), 3.29 (t, *J* = 6.6 Hz, 2H), 3.20 (dt, apparent q, *J* = 6.5 Hz, 2H), 1.66-1.46 (m, 4H), 1.45-1.29 (m, 4H); ^{19}F NMR (375 MHz, $CDCl_3$) δ -135.8 - -135.9 (m); ^{13}C NMR (100 MHz, $CDCl_3$) δ 156.4, 152.4 (d, *J* = 247.9 Hz), 147.8 (d, *J* = 11.0 Hz), 133.1 (d, *J* = 3.9 Hz), 120.9 (d, *J* = 6.9 Hz), 116.1 (d, *J* = 18.4 Hz), 113.7 (d, *J* = 1.9 Hz), 99.9, 66.4, 56.4, 43.0, 41.1, 31.2, 30.0, 26.3, 26.2; HRMS (ESI) m/z $[M + H]^{+}$ calcd for $C_{16}H_{22}FN_2O_4$ 325.1563; found 325.1555.

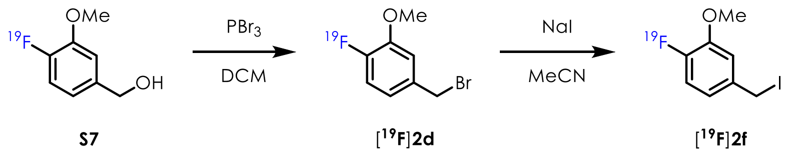

1-fluoro-4-(iodomethyl)-2-methoxybenzene ([^{19}**F**]**2f**)

Bromide [^{19}**F**]**2d** was prepared from alcohol **S7** (30 mg, 0.19 mmol) via the aforementioned procedure. To a solution of the obtained [^{19}**F**]**2d** in MeCN (3.8 mL) was added sodium iodide (85 mg, 0.57 mmol). After 48 hours, concentration and flash chromatography (20% EtOAc in hexane) afforded [^{19}**F**]**2f** (21.9 mg, 43% yield over 2 steps) as a pale-yellow oil: ^{1}H NMR (400 MHz, $CDCl_3$) δ 7.02-6.94 (m, 2H), 6.94-6.88 (m, 1H), 4.42 (s, 2H), 3.89 (s, 3H); ^{19}F NMR (375 MHz, $CDCl_3$) δ -135.2 - -135.4 (m); ^{13}C NMR (100 MHz, $CDCl_3$) δ 152.0 (d, *J* = 246.9 Hz), 147.8 (d, *J* = 11.0 Hz), 135.7 (d, *J* = 3.8 Hz), 121.2 (d, *J* = 7.0 Hz), 116.3 (d, *J* = 18.8 Hz), 114.1 (d, *J* = 2.2 Hz), 56.4, 5.2; HRMS (APCI/APPI) m/z [M+H] calcd for C_8H_9FIO 266.9682; found 266.9674.

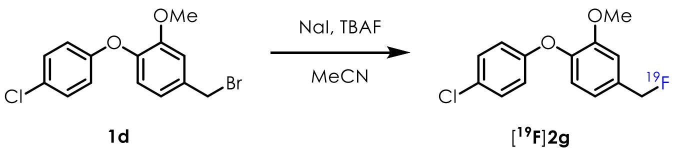

1-(4-chlorophenoxy)-4-(fluoromethyl)-2-methoxybenzene ([^{19}**F**]**2g**)

To a solution of bromide **1d** (43 mg, 0.13 mmol) in MeCN (2.6 mL) was added sodium iodide (58 mg, 0.39 mmol) and TBAF (1.1 mL, 1 M solution in THF, 1.1 mmol). After 23 hours, concentration and flash chromatography (9% EtOAc in hexane) afforded [^{19}**F**]**2g** (34.7 mg, 99% yield) as a colorless oil: ^{1}H NMR (400 MHz, $CDCl_3$) δ 7.25 (d, *J* = 9.1 Hz, 2H), 7.04 (s, 1H), 6.99-6.90 (m, 2H), 6.87 (d, *J* = 8.9 Hz, 2H), 5.36 (d, *J* = 47.9 Hz, 2H), 3.85 (s, 3H); ^{19}F NMR (375 MHz, $CDCl_3$) δ -204.7 (t, *J* = 48.0 Hz); ^{13}C NMR (100 MHz, $CDCl_3$) δ 156.5, 151.6, 145.2 (d, *J* = 3.4 Hz), 133.4 (d, *J* = 17.2 Hz), 129.7, 127.8, 121.0 (d, *J* = 1.3 Hz), 120.6 (d, *J* = 6.0 Hz), 118.6, 112.3 (d, *J* = 5.6 Hz), 84.5 (d, *J* = 166.8 Hz), 56.1; HRMS (APCI/APPI) m/z $[M]^{+}$ calcd for $C_{14}H_{12}ClFO_2$ 266.0510; found 266.0502.

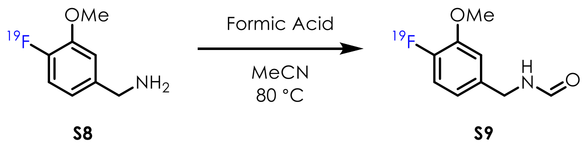

N-(4-fluoro-3-methoxybenzyl)formamide (**S9**)

To a solution of amine **S8** (300 mg, 1.93 mmol, liquid measured by weight) in MeCN (7.7 mL) was added formic acid (0.36 mL, 9.6 mmol). The mixture was heated at 80 °C. After 7 hours, mixture was cooled to room temperature, and saturated sodium bicarbonate aqueous solution (20 mL) was added. The mixture was extracted with EtOAc. Concentration and gradient flash chromatography (17% EtOAc in hexane to 50% EtOAc in hexane to 83% EtOAc in hexane) afforded **S9** (194 mg, 55% yield) as a white solid: ^{1}H NMR (500 MHz, $CDCl_3$) δ 8.27 (s, 1H), 7.02 (dd, *J* = 11.1, 8.2 Hz, 1H), 6.91 (dd, *J* = 8.0, 1.9 Hz, 1H), 6.83-6.75 (m, 1H), 5.84 (s, 1H), 4.44 (d, *J* =

6.0 Hz), 3.88 (s, 3H); ^{19}F NMR (470 MHz, $CDCl_3$) δ -136.4 - -136.7 (m); ^{13}C NMR (125 MHz, $CDCl_3$) δ 161.0, 152.1 (d, *J* = 245.7 Hz), 148.0 (d, *J* = 11.0 Hz), 134.1 (d, *J* = 4.1 Hz), 120.2 (d, *J* = 6.4 Hz), 116.2 (d, *J* = 18.4 Hz), 113.2 (d, *J* = 1.8 Hz), 56.4, 42.0; HRMS (ESI) m/z [M + Na] calcd for $C_9H_{10}FNO_2Na$ 206.0593, found 206.0586.

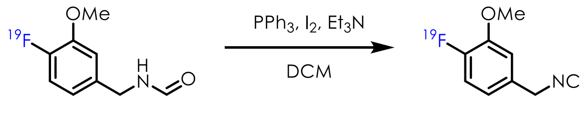

1-fluoro-4-(isocyanomethyl)-2-methoxybenzene ([**^{19}F]2i**)

To a solution of **S9** (30 mg, 0.16 mmol) in DCM (0.48 mL) was added triphenylphosphine (63 mg, 0.24 mmol), iodine (61 mg, 0.24 mmol), and triethylamine (67 µL, 0.48 mmol). After 1 hour, saturated sodium thiosulfate aqueous solution (5 mL) was added. The mixture was extracted with DCM. Concentration and gradient flash chromatography (9% EtOAc in hexane to 17% EtOAc in hexane) afforded the isocyanide [**^{19}F]2i** (10 mg, 37% yield) as a colorless oil: ^{1}H NMR (500 MHz, $CDCl_3$) δ 7.08 (dd, *J* = 10.9, 8.3 Hz, 1H), 6.96 (dd, *J* = 7.8, 2.1 Hz, 1H), 6.88-6.83 (m, 1H), 4.60 (s, 2H), 3.92 (s, 3H); ^{19}F NMR (470 MHz, $CDCl_3$) δ -135.17 - -135.27 (m); ^{13}C NMR (125 MHz, $CDCl_3$) δ 158.1 (t, *J* = 5.0 Hz), 152.4 (d, *J* = 247.5 Hz), 148.2 (d, *J* = 11.1 Hz), 128.7 (d, *J* = 3.8 Hz), 119.2 (d, *J* = 7.1 Hz), 116.5 (d, *J* = 18.4 Hz), 112.0, 56.5, 45.3 (d, *J* = 7.3 Hz); HRMS (APCI/APPI) m/z $[M]^{+}$ calcd for C_9H_8FNO 165.0590, found 165.0584.

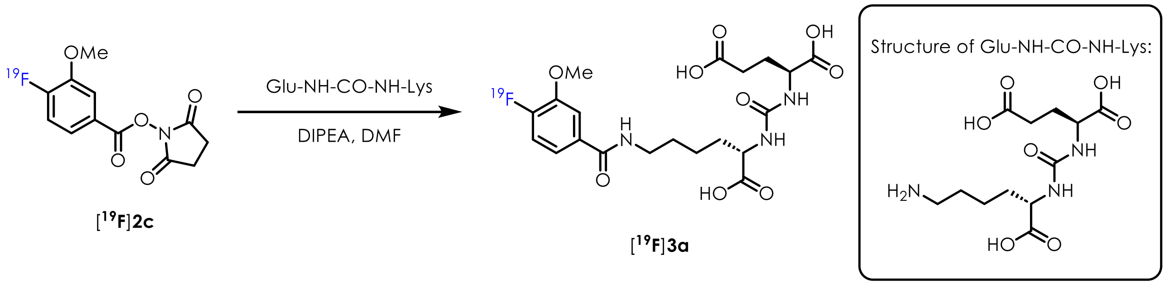

(((S)-1-carboxy-5-(4-fluoro-3-methoxybenzamido)pentyl)carbamoyl)-L-glutamic acid ([**^{19}F]3a**).

To a solution of [**^{19}F]2c** (0.2 mg, 0.75 µmol) in DMF (40 µL) was added Glu-NH-CO-NH-Lys (0.1 mg, 0.31 µmol, in 10 µL DMF). More DMF (150 µL) and DIPEA (10 µL) were then added. After 24 hours, the mixture was purified by HPLC (HPLC column 2, HPLC gradient elution method B), affording [**^{19}F]3a** in 32% yield (estimated by the crude HPLC trace with 212 nm detector, based on peak integration of [**^{19}F]3a** and all other peaks) HRMS (ESI) m/z $[M + Na]^{+}$ calcd for $C_{20}H_{26}FN_3O_9Na$ 494.1551; found 494.1540.

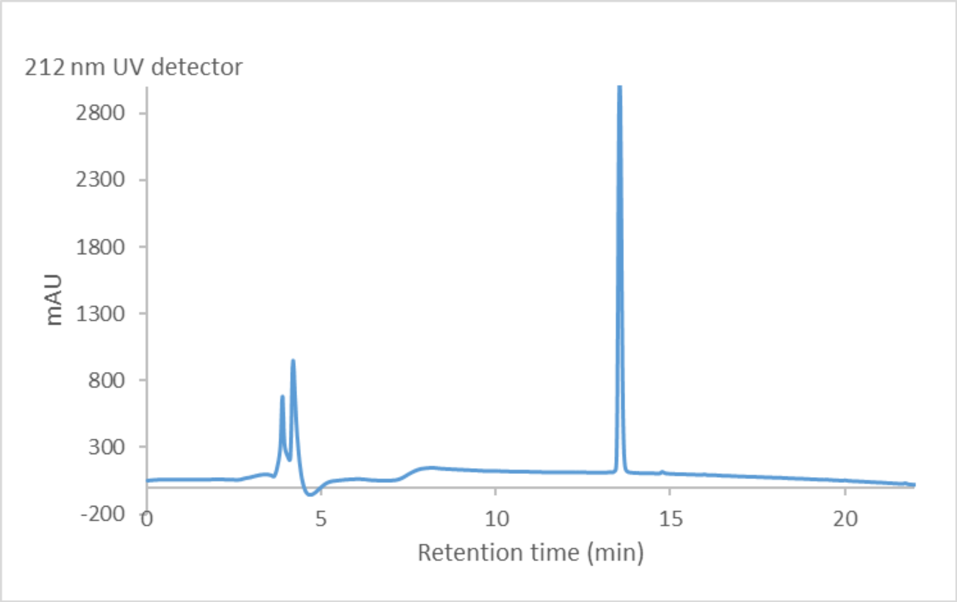

Figure S1. Quality control of [^{19}F]**3a** (retention time is 13.53 min, HPLC column 2, HPLC gradient elution method B)

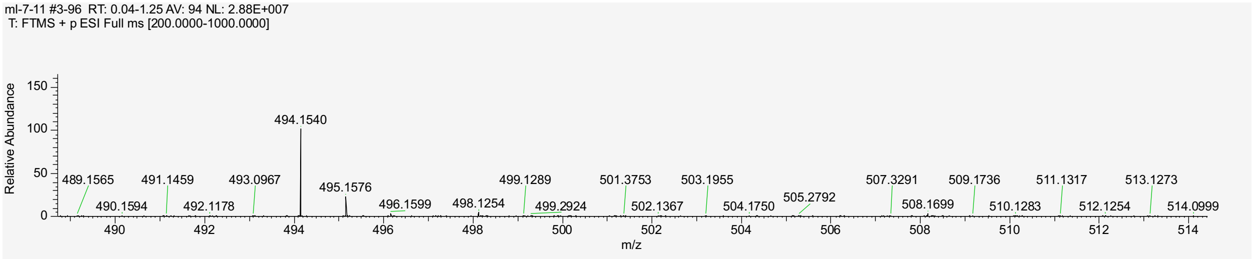

Figure S2. HRMS of [^{19}F]**3a**

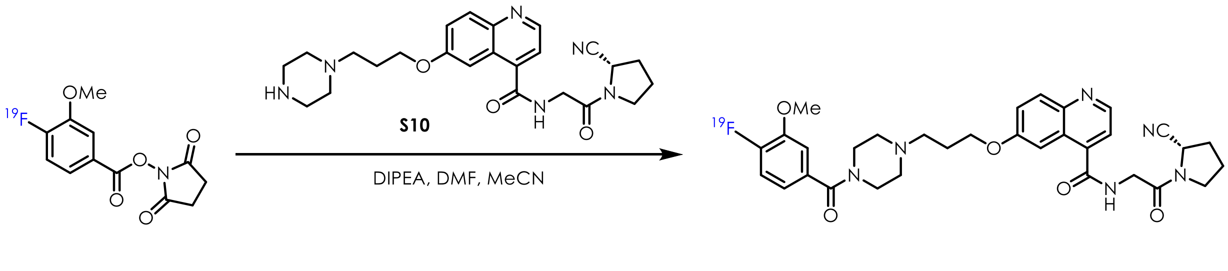

[^{19}F]**2c**

[^{19}F]**3b**

(S)-N-(2-(2-cyanopyrrolidin-1-yl)-2-oxoethyl)-6-(3-(4-(4-fluoro-3-methoxybenzoyl)piperazin-1-yl)propoxy)quinoline-4-carboxamide ([^{19}F]**3b**)

To a solution of [^{19}F]**2c** (0.2 mg, 0.75 µmol) in DMF (40 µL) was added FAPI **S10** (0.1 mg, 0.22 µmol, in 3.6 µL MeCN). DIPEA (10 µL) was then added. After 6 hours, the mixture was purified by HPLC (HPLC column 2, HPLC gradient elution method B), affording [^{19}F]**3b** in 38% yield (estimated by the crude HPLC trace with 254 nm detector, based on peak integration of [^{19}F]**3b** and all other peaks) HRMS (ESI) m/z $[M + H]^+$ calcd for $C_{32}H_{36}FN_6O_5$ 603.2731; found 603.2717.

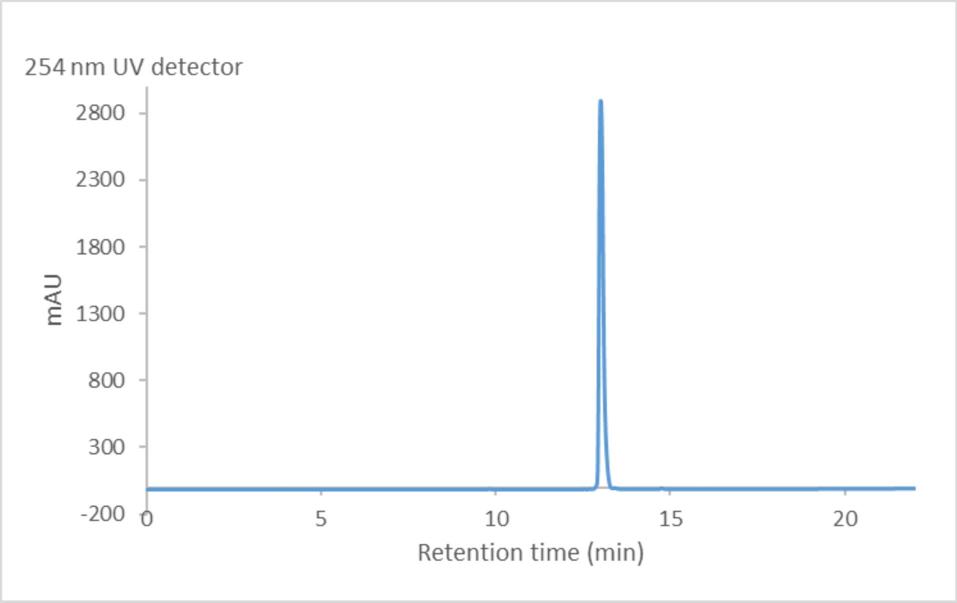

Figure S3. Quality control of [^{19}**F]3b** (retention time is 13.00 min, HPLC column 2, HPLC gradient elution method B)

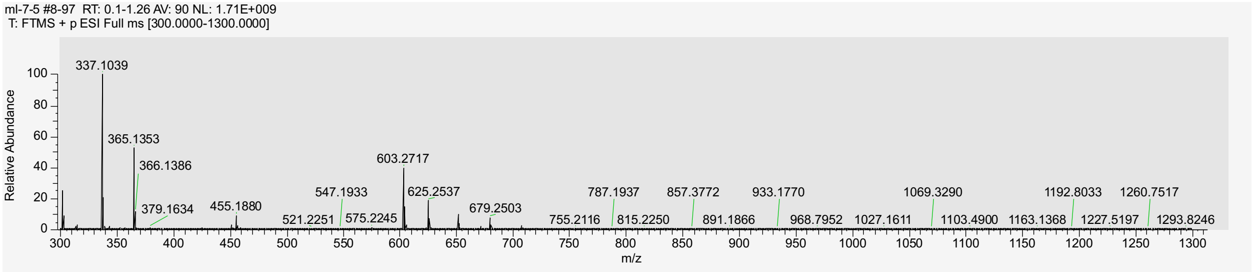

Figure S4. HRMS of [^{19}**F]3b**

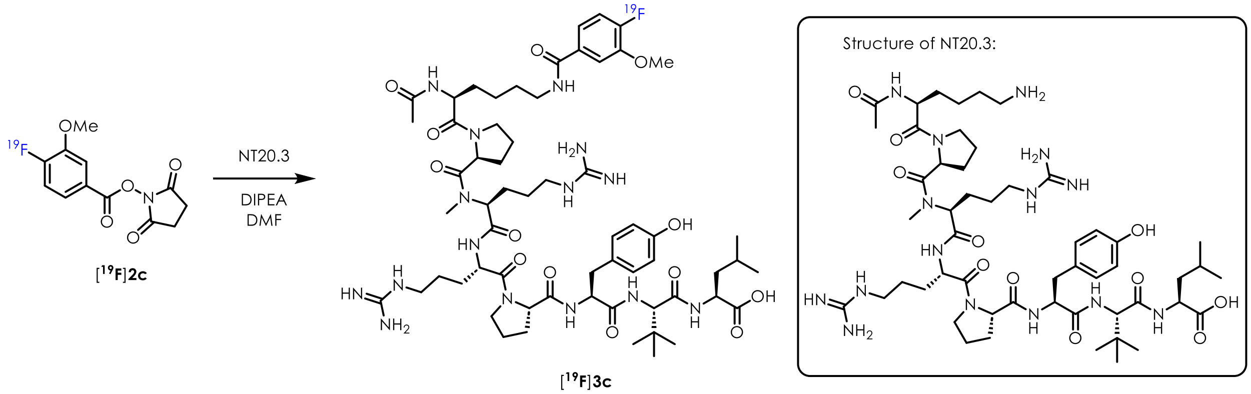

((S)-2-((S)-2-((S)-1-(N2-N2-acetyl-N6-(4-fluoro-3-methoxybenzoyl)-L-lysyl-L-prolyl-N2-methyl-L-arginyl-L-arginyl)pyrrolidine-2-carboxamido)-3-(4-hydroxyphenyl)propanamido)-3,3-dimethylbutanoyl)-L-leucine ([^{19}**F]3c**).

To a solution of [^{19}F]**2c** (0.2 mg, 0.75 µmol) in DMF (40 µL) was added NT20.3 (0.1 mg, 0.080 µmol, in 10 µL DMF). More DMF (150 µL) and DIPEA (10 µL) were then added. After 24 hours, the mixture was purified by HPLC (HPLC column 2, HPLC gradient elution method B), affording [^{19}F]**3c** in 70% yield (estimated by the crude HPLC trace with 254 nm detector, based on peak integration of [^{19}F]**3c** and all other peaks). HRMS (ESI) m/z [M + Na]$^+$ calcd for $C_{60}H_{92}FN_{15}O_{13}Na$ 1272.6881; found 1272.6939.

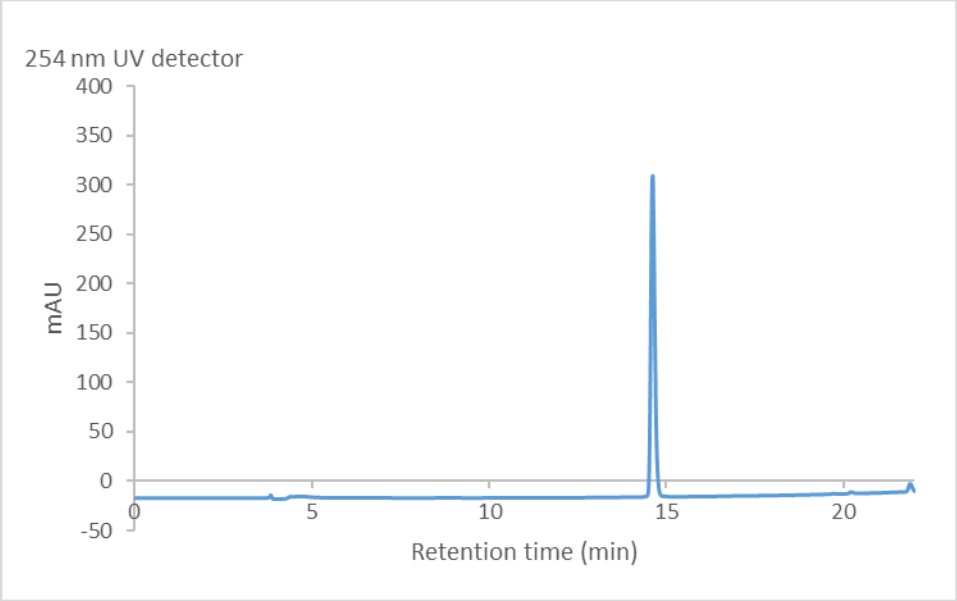

Figure S5. Quality control of [^{19}F]**3c** (retention time is 14.62 min, HPLC column 2, HPLC gradient elution method B)

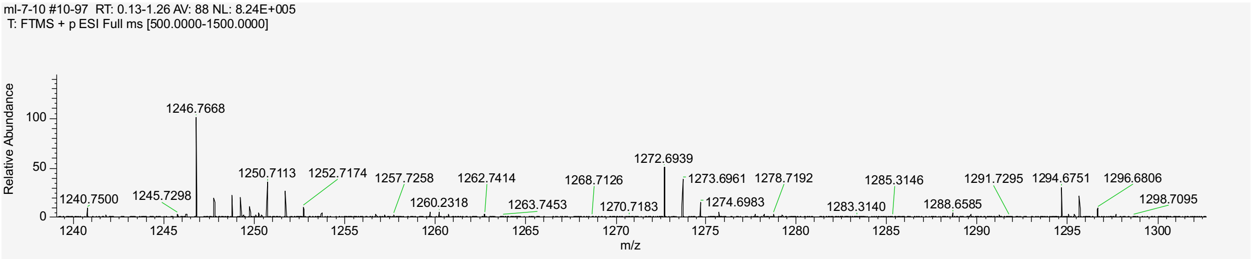

Figure S6. HRMS of [^{19}F]**3c**

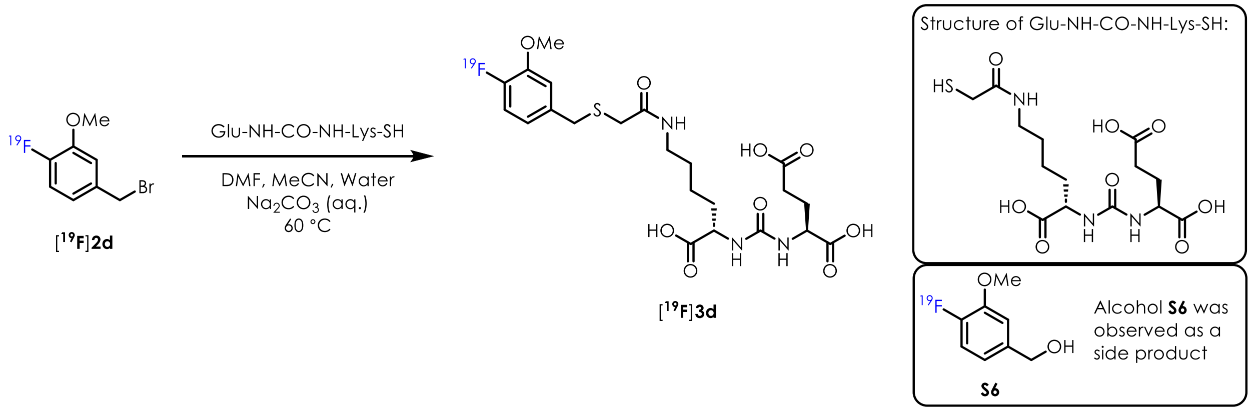

(10S,14S)-1-(4-fluoro-3-methoxyphenyl)-4,12-dioxo-2-thia-5,11,13-triazahexadecane-10,14,16-tricarboxylic acid ([^{19}F]**3d**)

To a stock solution of [^{19}**F**]**2d** (0.11 mg, 0.50 µmol, 4.4 µL 0.11 M stock solution) in a microcentrifuge tube was added Glu-NH-CO-NH-Lys-SH stock solution (0.10 mg, 0.25 µmol, 3.3 µL 0.078 M stock solution). MeCN (50 µL), water (50 µL), and sodium carbonate aqueous buffer solution (50 µL, 0.5 M) were then added. The mixture was incubated at 60 °C and 400 rpm. After 1 hour, HPLC purification (HPLC column 2, HPLC gradient elution method B) afforded [^{19}**F**]**3d** in 38% yield (estimated by the crude HPLC trace with 212 nm detector, based on peak integration of [^{19}**F**]**3d** and all other major peaks after solvent front). HRMS (ESI) m/z $[M + H]^+$ calcd for 532.1766, found 532.1760.

Note: [^{19}**F**]**3d** and **S6** do not have baseline separation under HPLC gradient elution method B. Baseline separation of the two compounds can be achieved with HPLC gradient elution method C. The ratio between water adduct **S6** and desired [^{19}**F**]**3d** is 34:100 (based on HPLC peak integration with 212 nm detector).

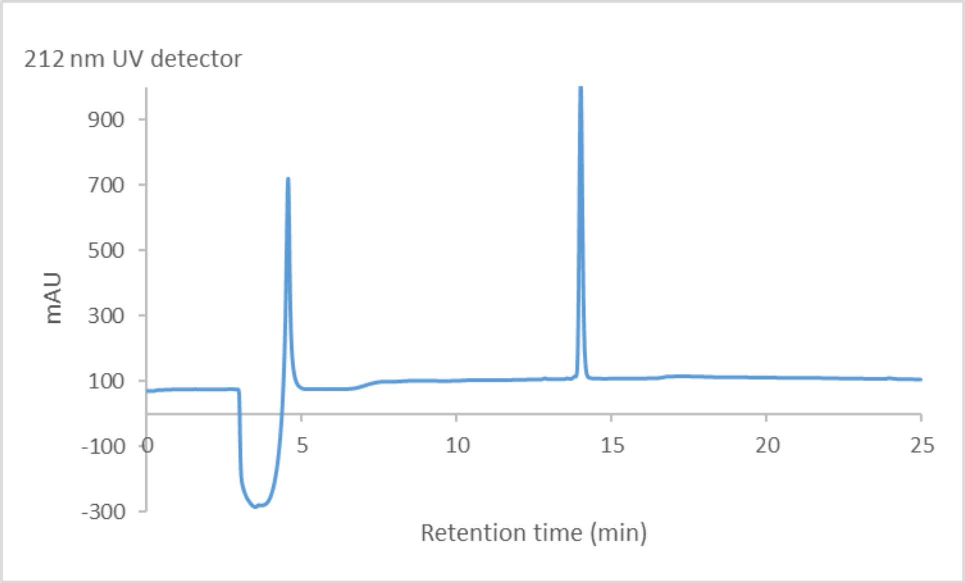

Figure S7. Quality control of [^{19}F]**3d** (retention time is 14.0 min, HPLC column 2, HPLC gradient elution method C, retention time of **S6** is 12.8 min)

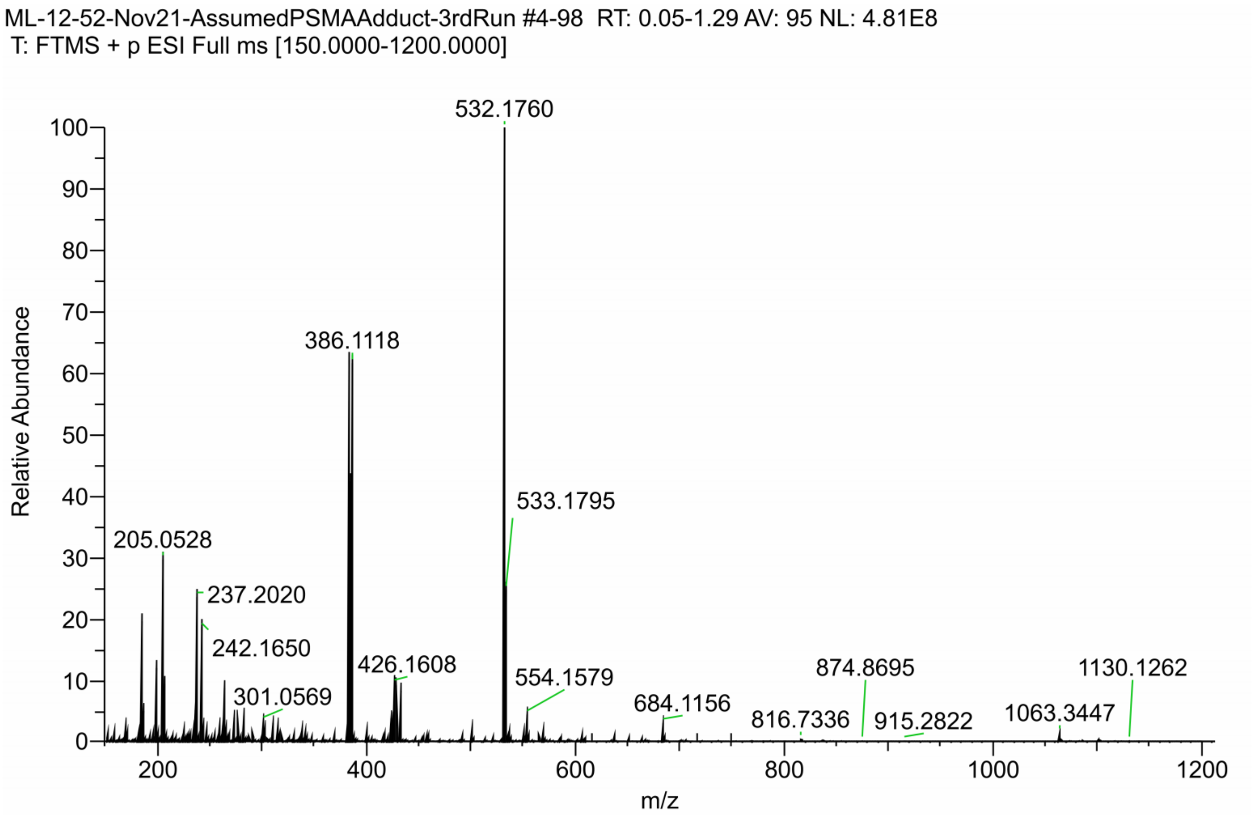

Figure S8. HRMS of [^{19}F]**3d**

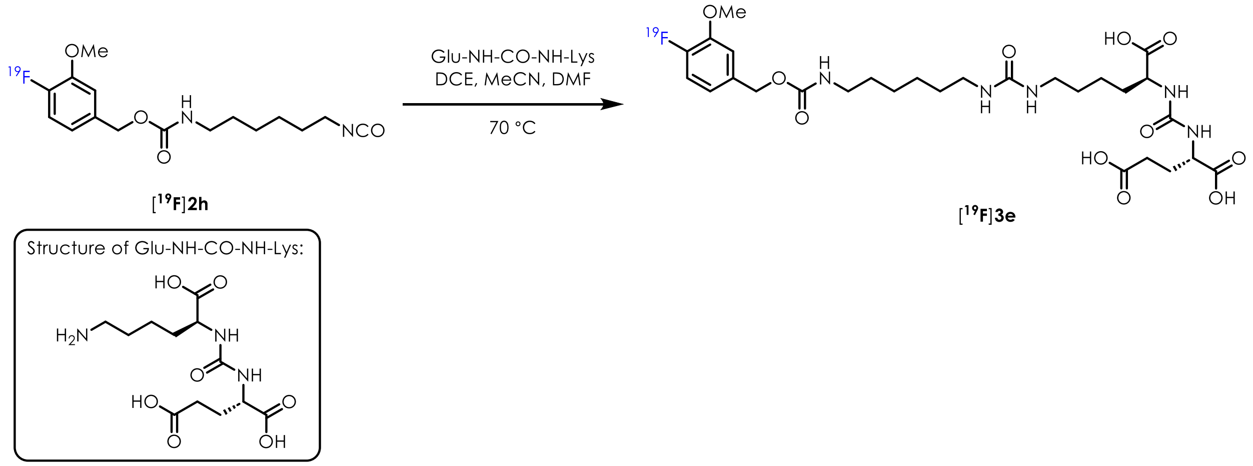

[^{19}F]**2h**

[^{19}F]**3e**

(18S,22S)-1-(4-fluoro-3-methoxyphenyl)-3,12,20-trioxo-2-oxa-4,11,13,19,21-pentaazatetracosane-18,22,24-tricarboxylic acid ([^{19}F]**3e**)

The solution of [^{19}F]**2h** (1 mg, 3 µmol) in DCE (20 µL) and the solution of Glu-NH-CO-NH-Lys (1 mg, 3 µmol) in MeCN (200 µL) and DMF (200 µL) were mixed in a microcentrifuge tube. The mixture was incubated at 70 °C and 300 rpm. After 1 hour, HPLC purification (HPLC column 2, HPLC gradient elution method B) afforded [^{19}**F**]**3e** in 22% yield (estimated by the crude HPLC trace with 254 nm detector, based on peak integration of [^{19}**F**]**3e** and all other major peaks). HRMS (ESI) m/z [M + Na]$^+$ calcd for $C_{28}H_{42}FN_5O_{11}Na$ 666.2763, found 666.2755.

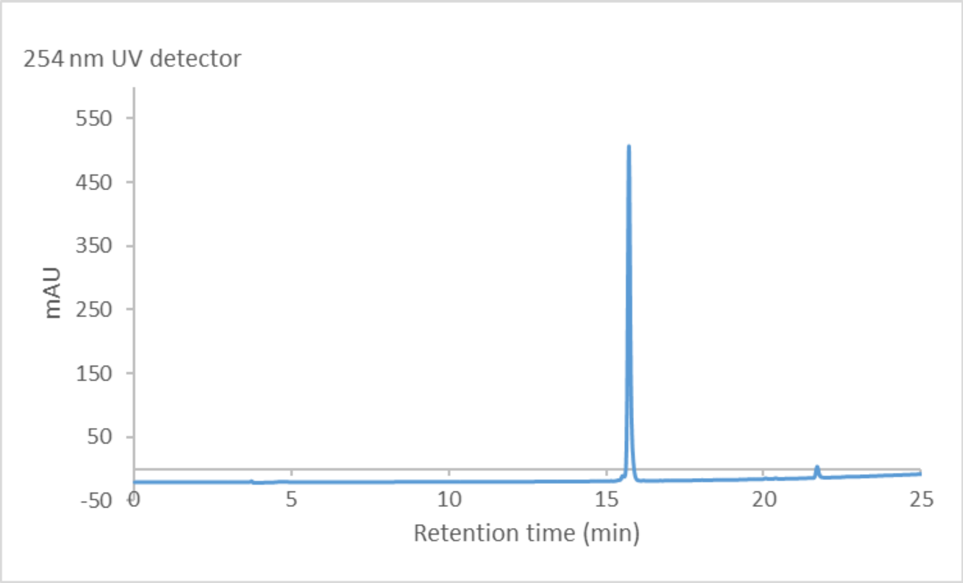

Figure S9. Quality control of [^{19}**F**]**3e** (retention time is 15.72 min, HPLC column 2, HPLC gradient elution method B)

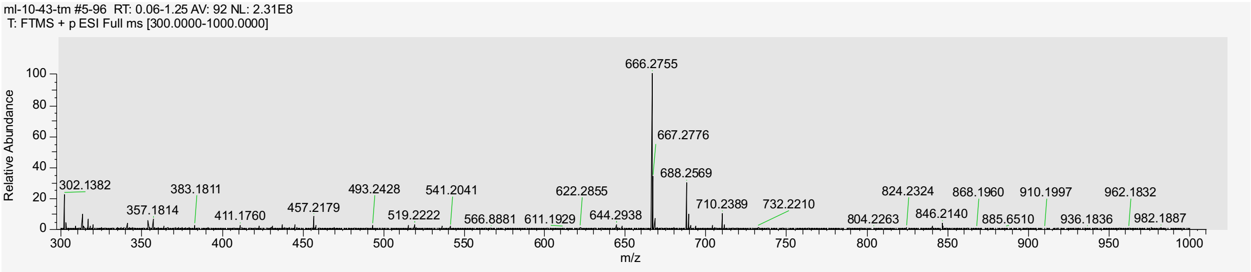

Figure S10. HRMS of [^{19}**F**]**3e**

Molar activity measurement

Molar activity was calculated using the standard curve of [^{19}F]**2c**, which was created from HPLC traces (with 254 nm detector) of a series of [^{19}F]**2c** standard solutions. The HPLC purified [^{18}F]**2c** was then analyzed by HPLC again for quality control, the UV area (254 nm detector) overlapping with the desired radio peak was recorded.

Injected [^{19}F]**2c** (µg)	UV Area (mAU·s)
1	822.6
0.4	344.1
0.2	176.8
0.1	94.5
0.05	51.4
0.01	16.1

Table **S1**. Standard [^{19}F]**2c** solution and UV area from HPLC

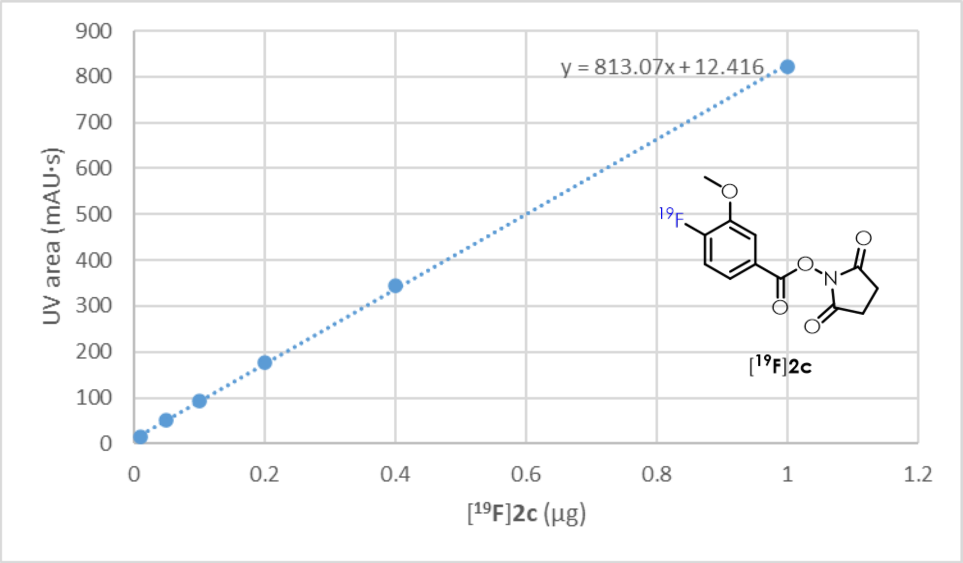

Table **S2**. Standard curve

The UV area of [^{18}F]**2c** was recorded to be 16.6 mAU·s. The mole number was calculated to be 1.93 X 10^{-5} µmol. Decay corrected (EOB) activity was 46.8 µCi (or 1.73 MBq). The molar activity was calculated to be 2.42 Ci/µmol (or 89.6 GBq/µmol).

Radiochemistry

General photoredox method

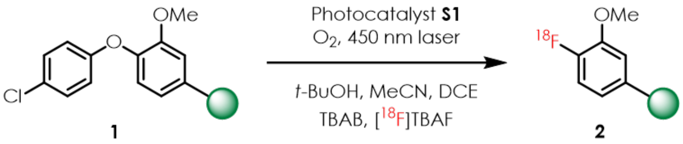

A solution of precursor **1** (0.05 mmol) in a solvent mixture (*t*-butyl alcohol:DCM:MeCN = 4:3:1, 800 µL or 400 µL) was prepared in a 5 mL V vial in the open air. The photocatalyst **S1** (1.5 mg, 0.0025 mmol) and tetrabutylammonium bicarbonate solution (60 mg/mL solution in MeCN, 25 µL, 15 µL or 0 µL) were then added. [^{18}F]TBAF/MeCN solution (typically 10 – 20 mCi, or 370 MBq to 740 MBq) was added last. A balloon (filled with pure oxygen or air) was attached to a needle, which was inserted into the reaction mixture and provided steady oxygen (or air) flow in a bubbling manner. The V vial containing the mixture was then irradiated top-down with a laser (MDL-D-450, 450 nm, 3.5 W, 30 min or 20 min irradiation) that is placed about 5 cm above the V vial. The irradiation process was performed at room temperature. An aliquot of the resulting mixture was analyzed and purified by HPLC, furnishing the corresponding ^{18}F-labeled synthons. All radiochemical yields (RCYs) reported are decay corrected. Process for RCY calculation is described on page 4 of SI. Example of RCY calculation is described below.

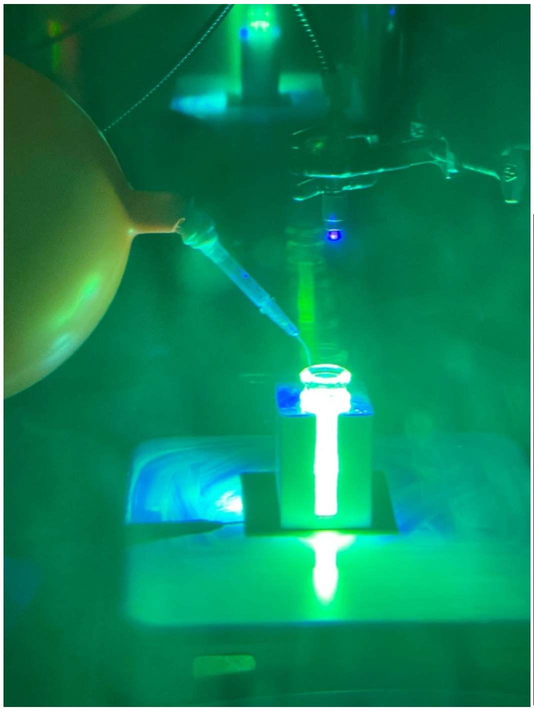

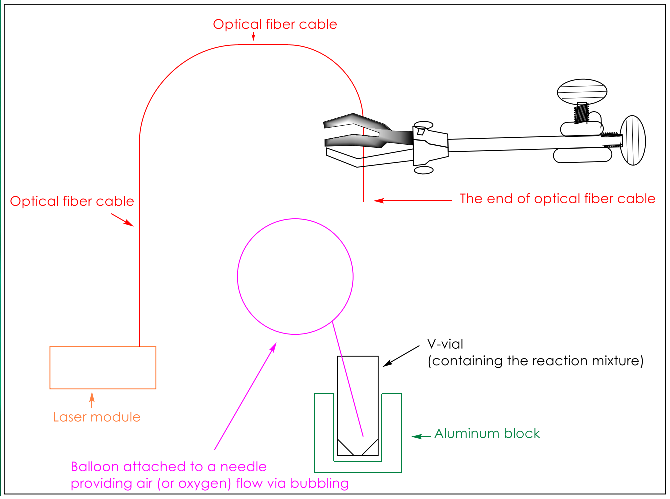

Figure S11. Reaction setup with 450 nm laser irradiating top-down. Left: picture was taken through lead glass shielding, actual color of the light appears different without lead glass; Right: explanation of the reaction setup, the laser was generated in the laser module, and directed into the V-vial via optical fiber cable. The end of the optical fiber cable was placed about 5 cm above the top of the V-vial.

Each radiochemical yield (RCY) was calculated based on the radioactivity of an aliquot of the crude mixture that was injected into the HPLC, and the radioactivity that was collected after the HPLC. Below is an example of RCY calculation process: after laser irradiation, an aliquot of the crude mixture containing radiolabeled succinimidyl ester [^{18}F]**2c** was transferred into a clean microcentrifuge tube, and diluted in MeCN and water. The radioactivity of this sample was measured to be 1409 μCi (52.13 MBq) by the dose calibrator (denoted as α). This sample was then injected into the HPLC, and the material containing [^{18}F]**2c** was collected after the HPLC column. The radioactivity of the collected material was measured to be 524 μCi (19.4 MBq) by the dose calibrator (denoted as β). The time difference between the first radioactivity measurement (for the HPLC injection sample) and the second radioactivity measurement (for the material collected from the HPLC) was recorded to be 11 min. The decay corrected radioactivity of the HPLC collected material (denoted as β') was calculated to be 562 μCi (20.8 MBq) from the measured β and the time difference (524 μCi activity decay corrected by 11 min). The decay corrected RCY was calculated by dividing β' by α (562 μCi divided by 1409 μCi, or 20.8 MBq divided by 52.13 MBq), which is 39.9%. The RCYs of two other succinimidyl ester labeling reactions were calculated by the same process to be 28.2% and 20.8% respectively. The average RCY was calculated as (39.9%+28.2%+20.8%)/3=29.6%, with 9.6% deviation.

We believe it is important to use proper terminology in reporting radiochemistry results. The misuse of terminologies may convey inaccurate information and hinder the communication of scientific results. The terminologies used in this manuscript follows the guidelines established by experts as consensus nomenclature rules.[3] Other efforts in unifying radiochemistry terminologies are also available.[4]

Radiosyntheses

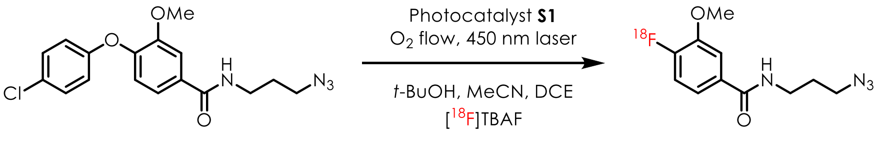

1a [^{18}F]**2a**

[^{18}F]2a was prepared via general photoredox procedure (800 μL solvent mixture, 15 μL TBAB/MeCN, oxygen flow and 30 min laser irradiation).

RCY #1: 30.6%

RCY #2: 62.7%

RCY #3: 53.2%

Average RCY: 48.8±22.7% (n=3)

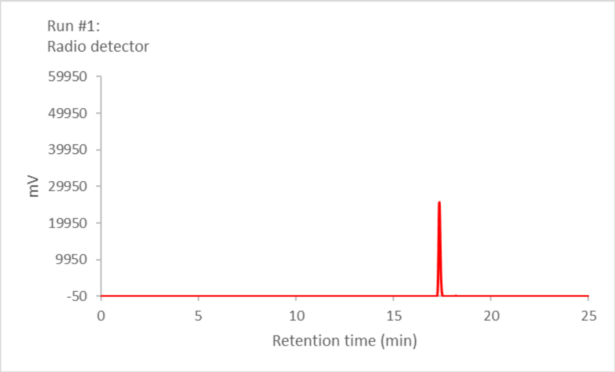

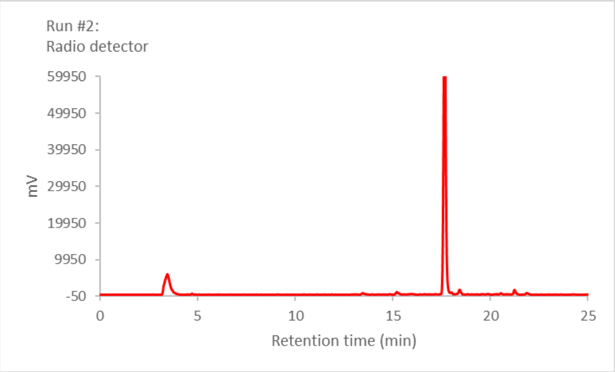

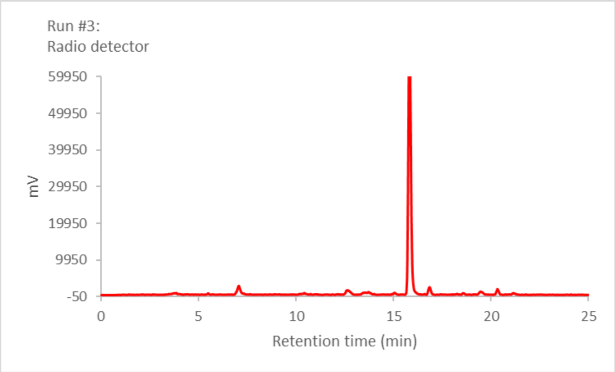

Figure S12. Crude radio-HPLC traces for [^{18}F]**2a** (retention time is 17.7 min and 15.8 min, HPLC column 1, HPLC gradient elution method B)

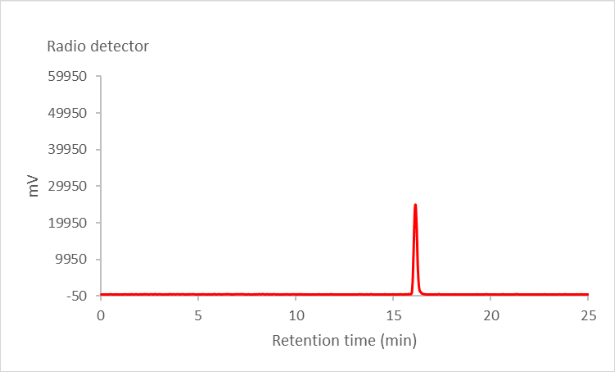

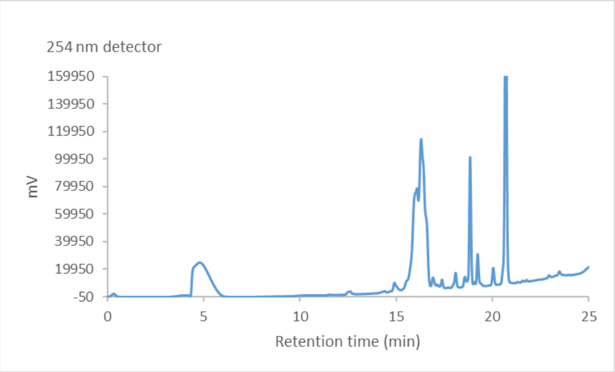

Figure S13. [^{18}F]**2a** QC (HPLC column 1, HPLC gradient elution method B)

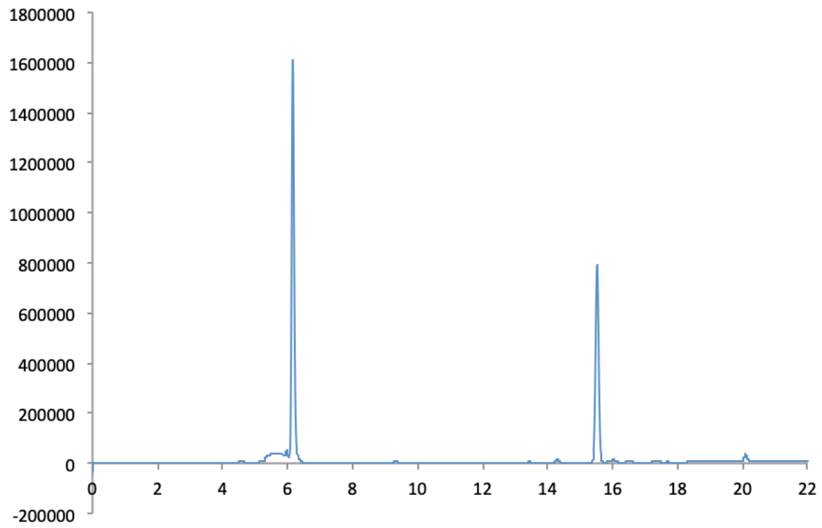

Figure S14. [^{19}F]**2a** reference QC (HPLC column 1, HPLC gradient elution method B, 254 nm detector)

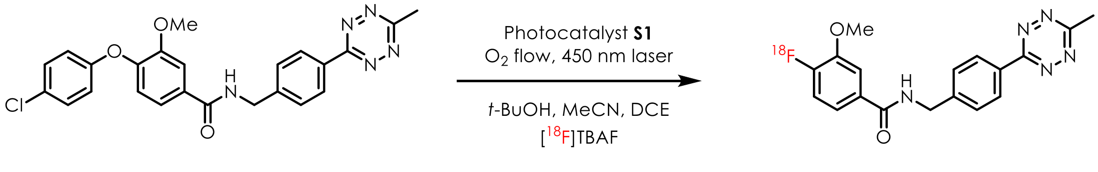

[^{18}F]2b was prepared via general photoredox procedure (800 µL solvent mixture, 15 µL TBAB/MeCN, oxygen flow and 30 min laser irradiation) in 8.7% RCY (n=1)

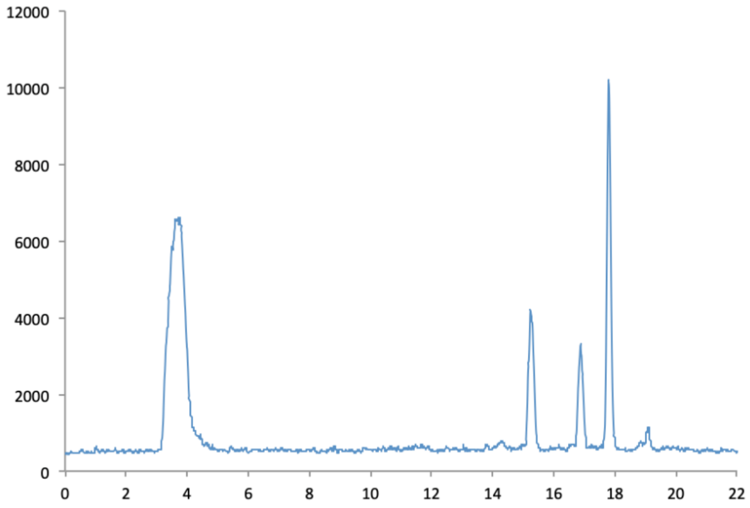

Figure S15. Crude radio-HPLC trace for [^{18}F]**2b** (retention time is 17.8 min, HPLC column 1, HPLC gradient elution method B)

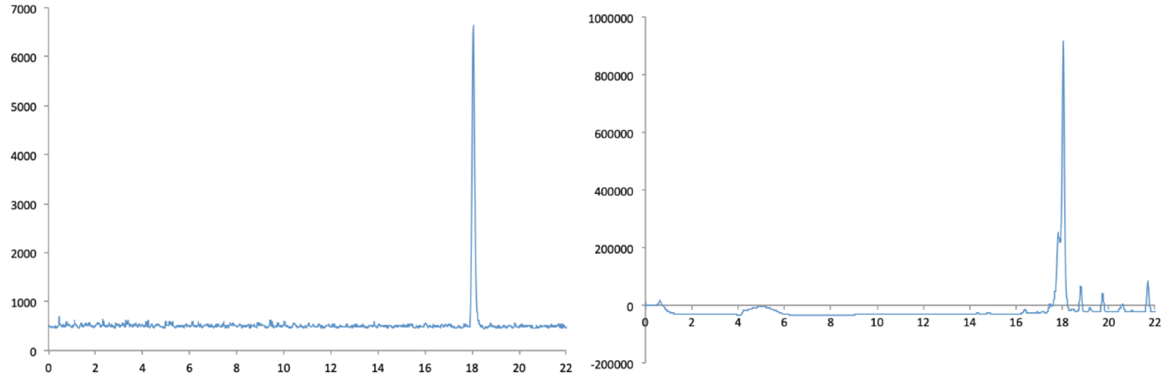

Figure S16. [^{18}F]**2b** QC (left: radio trace; right: UV trace with 254 nm detector; HPLC column 1, HPLC gradient elution method B)

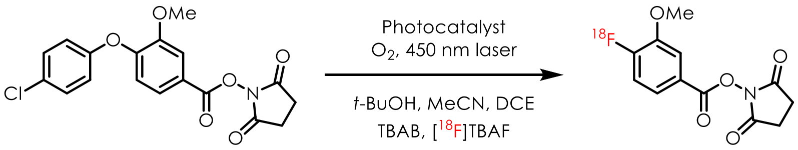

1c [^{18}F]**2c**

[^{18}F]2c was initially prepared via general photoredox procedure (800 µL solvent mixture, 15 µL TBAB/MeCN, oxygen flow and 30 min laser irradiation) in 16.3±1.7% (n=3). Due to the stability concern of **[^{18}F]2c** under basic conditions, TBAB/MeCN additive was later removed from the reaction condition. This synthon was then synthesized via general photoredox procedure (800 µL solvent mixture, no TBAB/MeCN, air flow and 20 min laser irradiation) in 29.6±9.6% (n=3) RCY.

Initial photoredox procedure (800 µL solvent mixture, 15 µL TBAB/MeCN, oxygen flow and 30 min laser irradiation):

Run #1: 18.3%

Run #2: 15.6%

Run #3: 15.1%

Average RCY: 16.3±1.7% (n=3)

Modified photoredox procedure (800 µL solvent mixture, no TBAB/MeCN, air flow and 20 min laser irradiation):

Run #1: 28.2%

Run #2: 39.9%

Run #3: 20.8%

Average RCY: 29.6±9.6% (n=3)

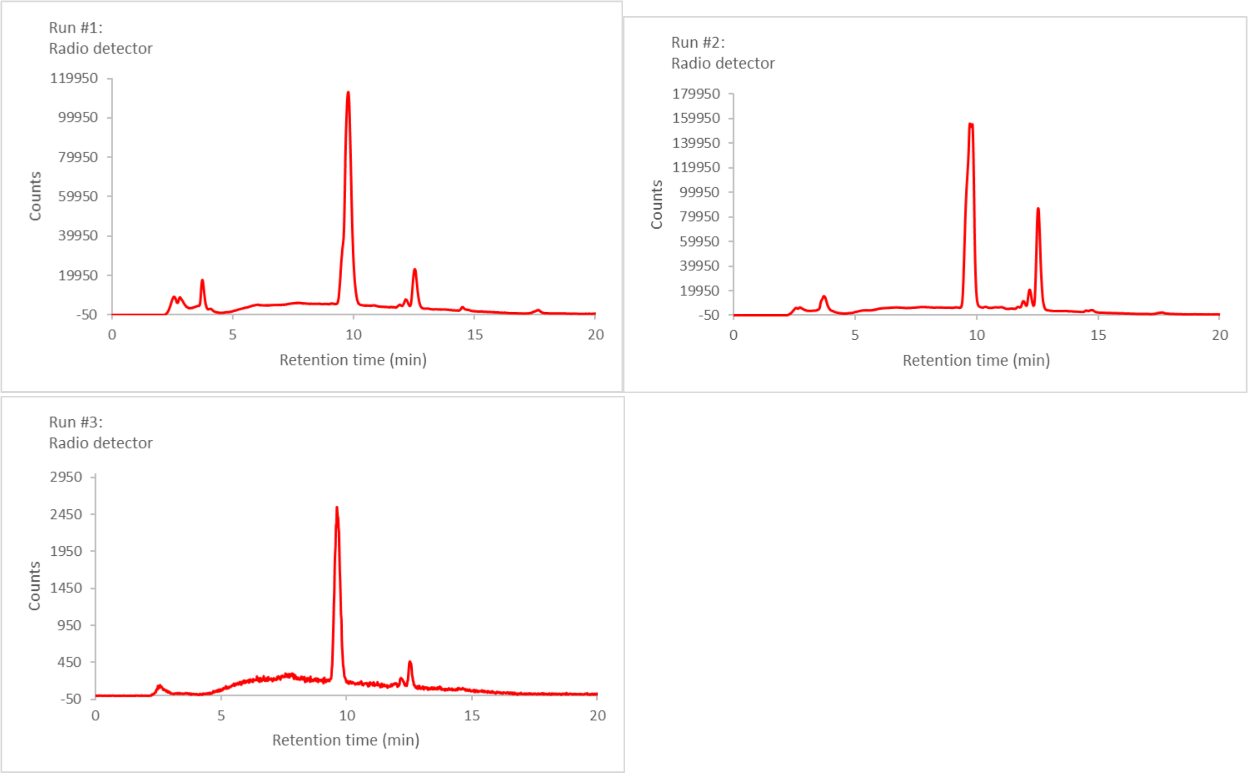

Figure S17. Crude radio-HPLC traces for [^{18}F]**2c** (Modified photoredox procedure: 800 µL solvent mixture, no TBAB/MeCN, air flow and 20 min laser irradiation; HPLC column 2; HPLC gradient elution method A; retention time is 9.77 min)

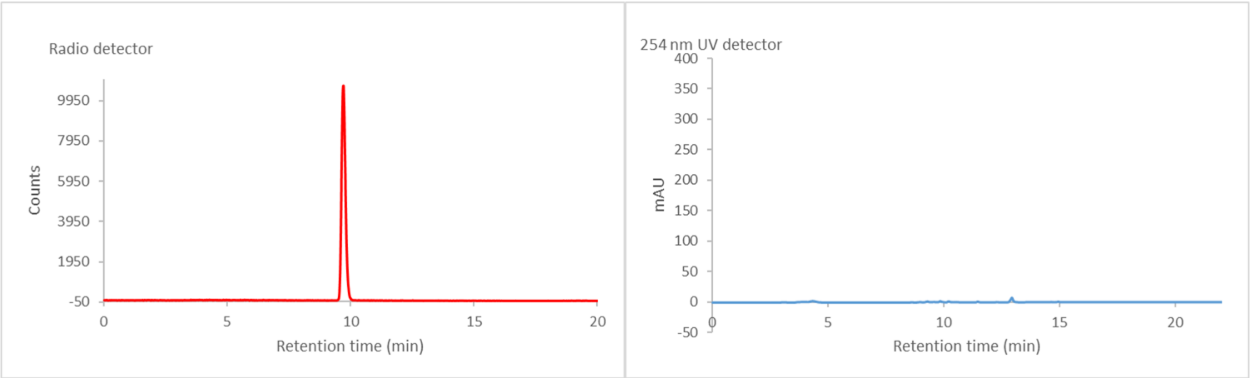

Figure S18. [^{18}F]**2c** QC (HPLC column 2; HPLC gradient elution method A)

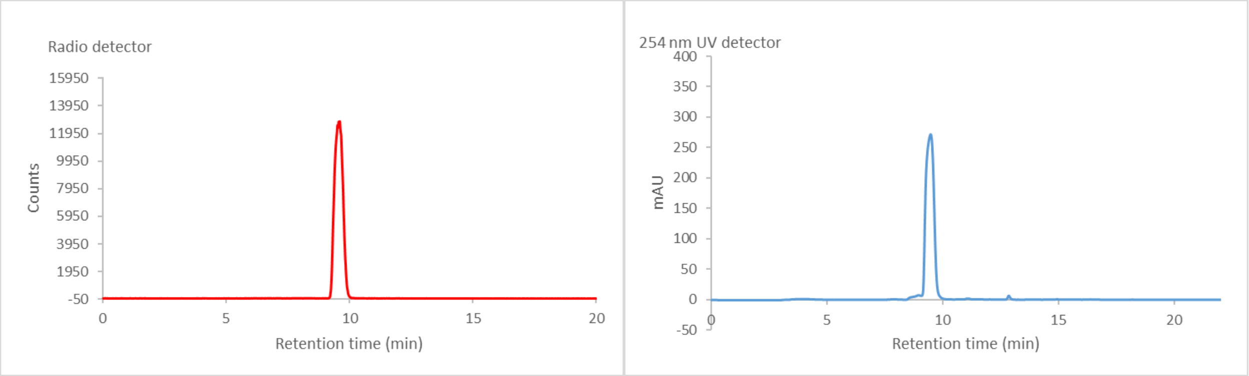

Figure S19. [^{18}F]**2c** Co-injection (HPLC column 2; HPLC gradient elution method A)

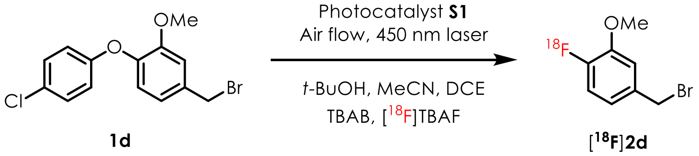

[^{18}F]**2d** was prepared from bromide precursor **1d** via general photoredox procedure (400 µL solvent mixture, 25 µL TBAB/MeCN, air flow and 20 min laser irradiation) in 51.3±9.7% (n=3) RCY.

The radio product [^{18}F]**2d** is mildly volatile. The heat from the laser slowly evaporated the solvent mixture, and [^{18}F]**2d** could also evaporate if solvent volume was reduced to less than 200 µL. The solvent mixture was refilled every 4 min during the laser irradiation to maintain the 400 µL reaction mixture volume. No radioactivity loss was observed when solvent refill was performed. The estimated RCC of side product [^{18}F]**2g** was calculated based on the isolated RCY of [^{18}F]**2d** and the radio-HPLC peak integration ratio between [^{18}F]**2d** and [^{18}F]**2g**.

[^{18}F]**2d** RCY Run #1: 62.4%   Estimated RCC of [^{18}F]**2g** Run #1: 6.2%

[^{18}F]**2d** RCY Run #2: 47.1%   Estimated RCC of [^{18}F]**2g** Run #2: 23.6%

[^{18}F]**2d** RCY Run #3: 44.5%   Estimated RCC of [^{18}F]**2g** Run #3: 16.9%

Average RCY of [^{18}F]**2d**: 51.3±9.7% (n=3), average estimated RCC of [^{18}F]**2g**: 15.6±8.8% (n=3).

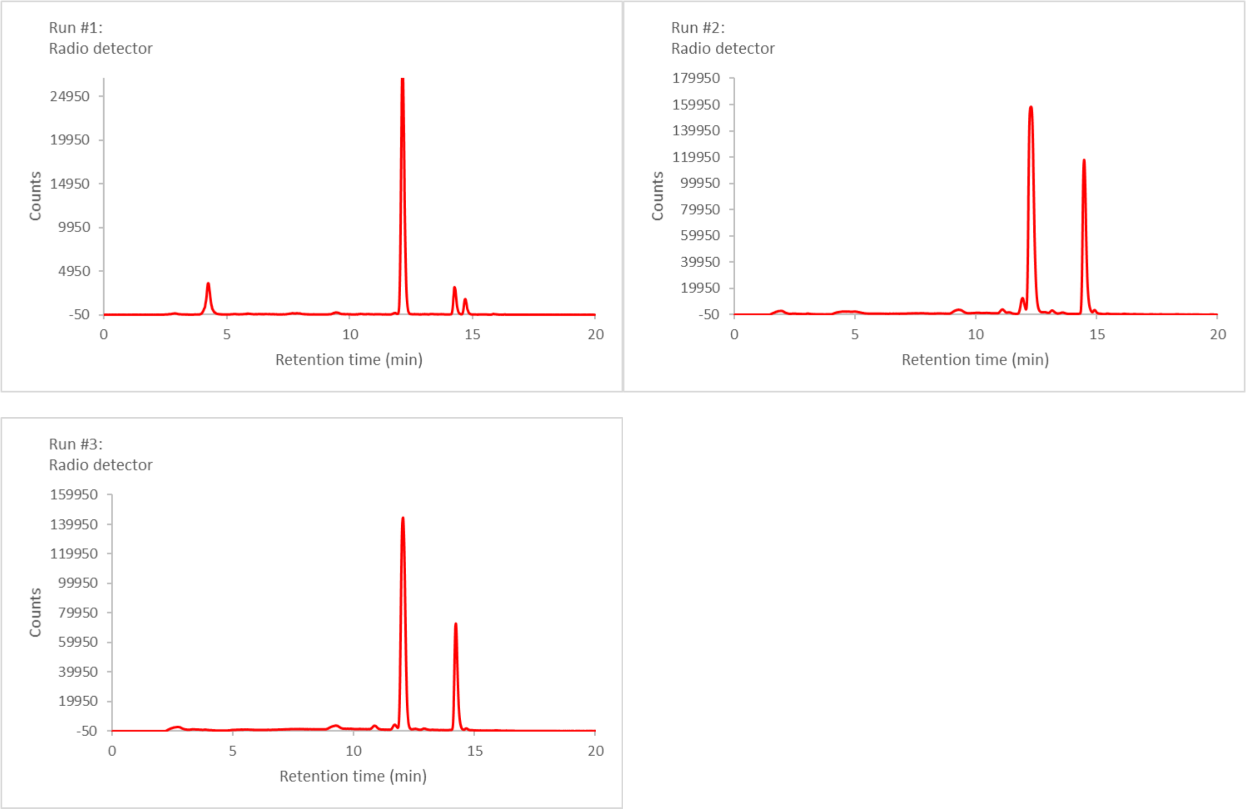

Figure S20. Crude radio-HPLC traces for [^{18}F]**2d** (HPLC column 2; HPLC gradient elution method A; retention time is 12.17 min)

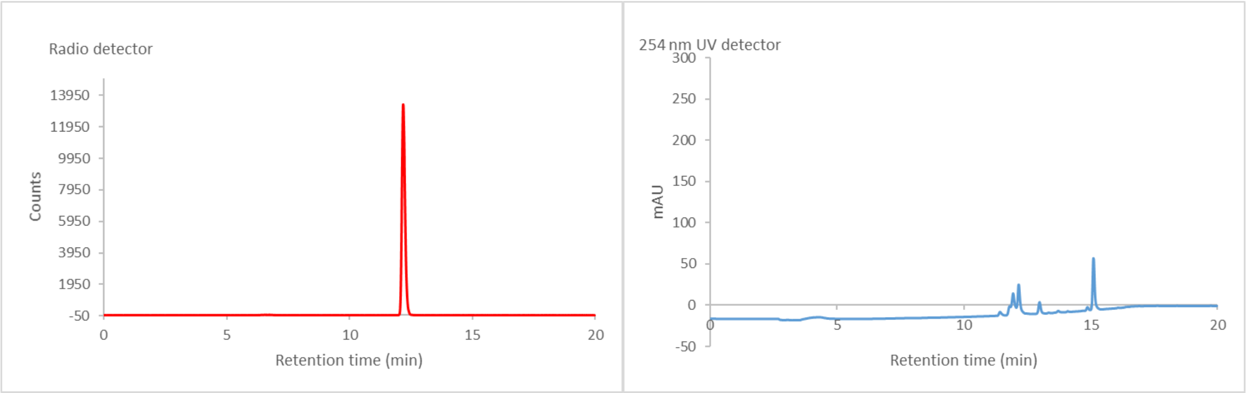

Figure S21. [^{18}F]**2d** QC (HPLC column 2; HPLC gradient elution method A)

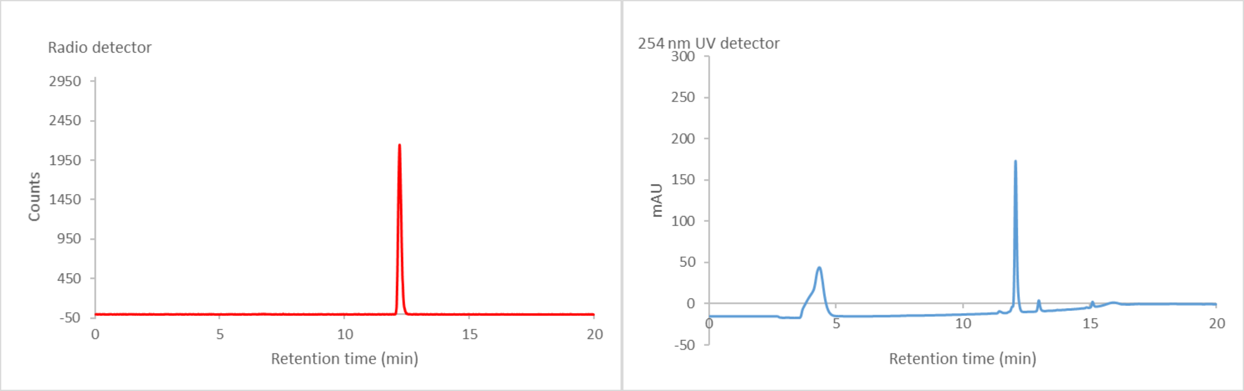

Figure S22. [^{18}F]**2d** co-injection (HPLC column 2; HPLC gradient elution method A)

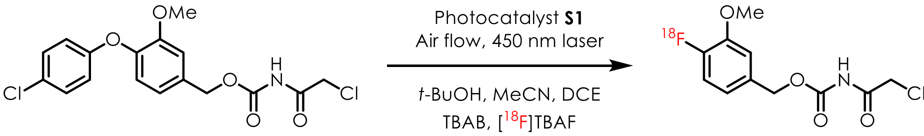

1e [^{18}F]**2e**

[^{18}F]**2e** was prepared from chloride precursor **1e** via general photoredox procedure (400 µL solvent mixture, 25 µL TBAB/MeCN, air flow and 20 min laser irradiation) in 23.2% RCY.

RCY #1: 23.2%

RCY #2: 22.3%

RCY #3: 16.8%

Average RCY: 20.8±3.5% (n=3)

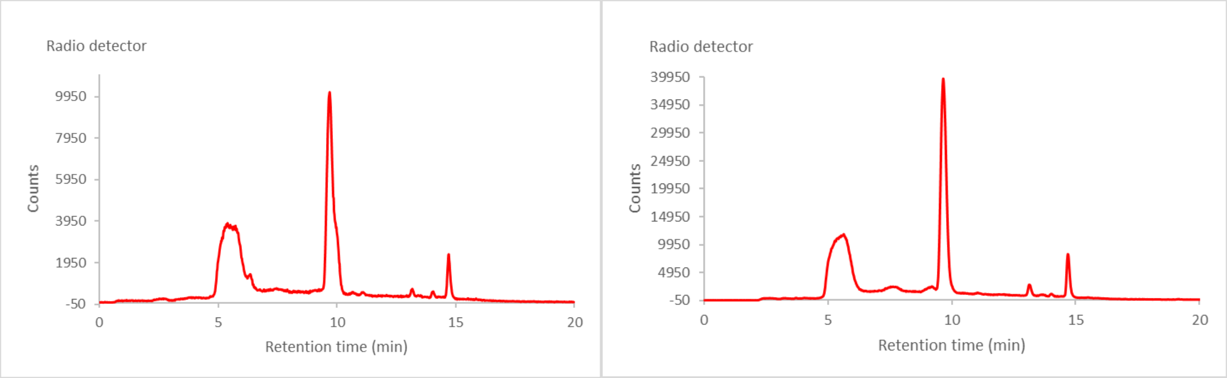

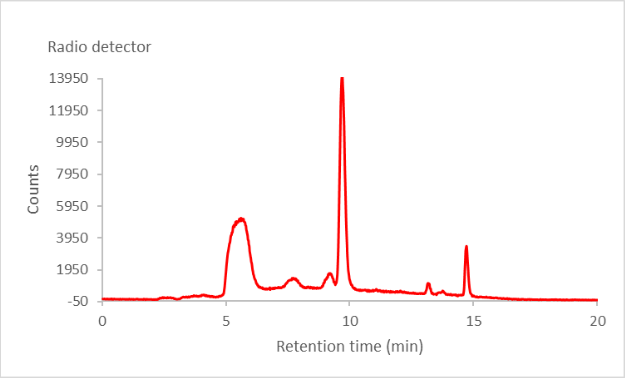

Figure S23. Crude radio-HPLC trace for [^{18}F]**2e** (HPLC column 2; HPLC gradient elution method A; retention time is 9.70 min)

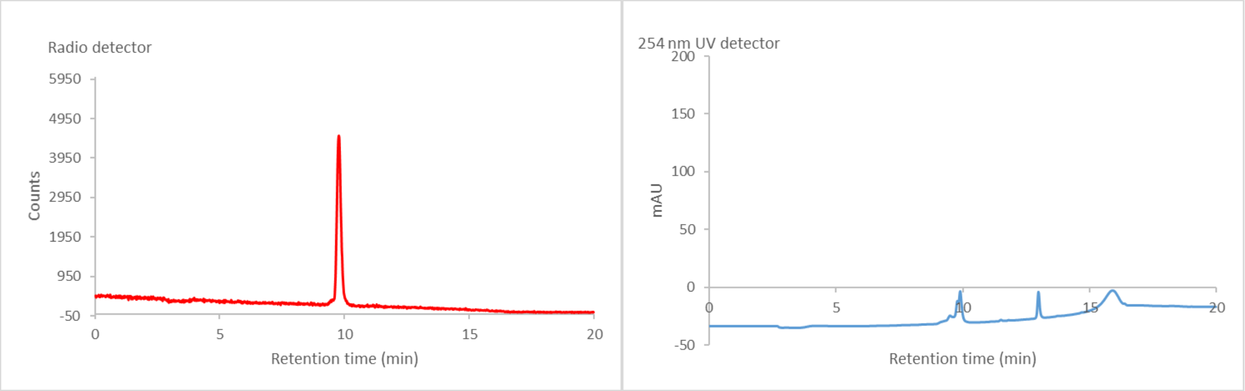

Figure S24. [^{18}F]**2e** QC (HPLC column 2; HPLC gradient elution method A)

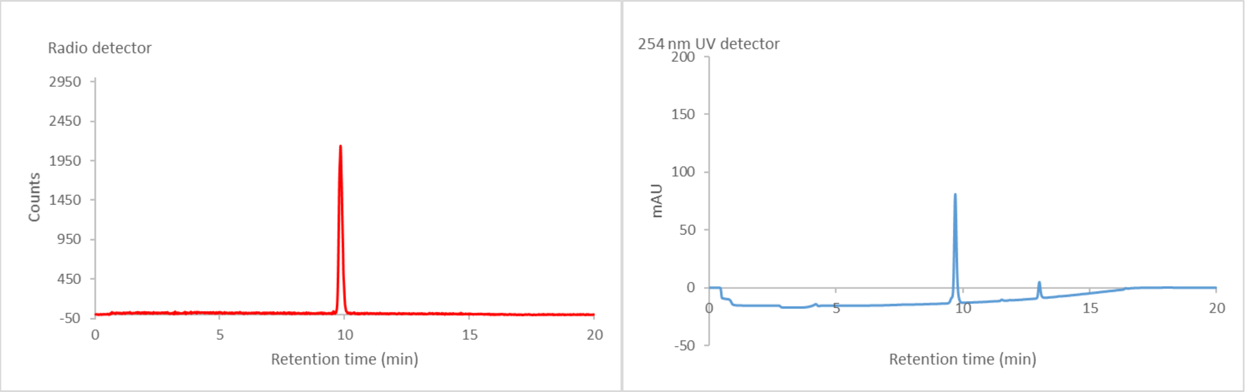

Figure S25. [^{18}F]**2e** co-injection (HPLC column 2; HPLC gradient elution method A)

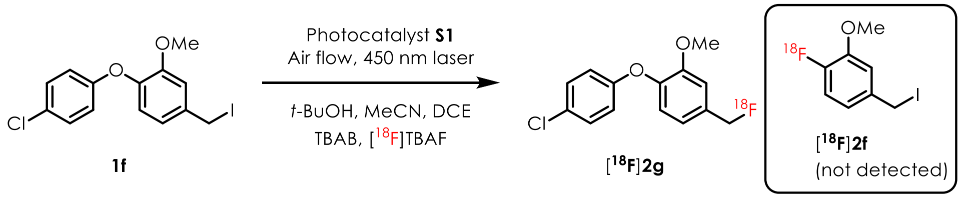

[^{18}F]**2g** was generated from iodide precursor **1f** via general photoredox procedure (400 µL solvent mixture, 25 µL TBAB/MeCN, air flow and 20 min laser irradiation) in 14.2±7.4% (n=2) RCY. The desired radio product [^{18}F]**2f** was not detected.

Run #1: 19.5%

Run #2: 9.0%

Average RCY: 14.2±7.4% (n=2)

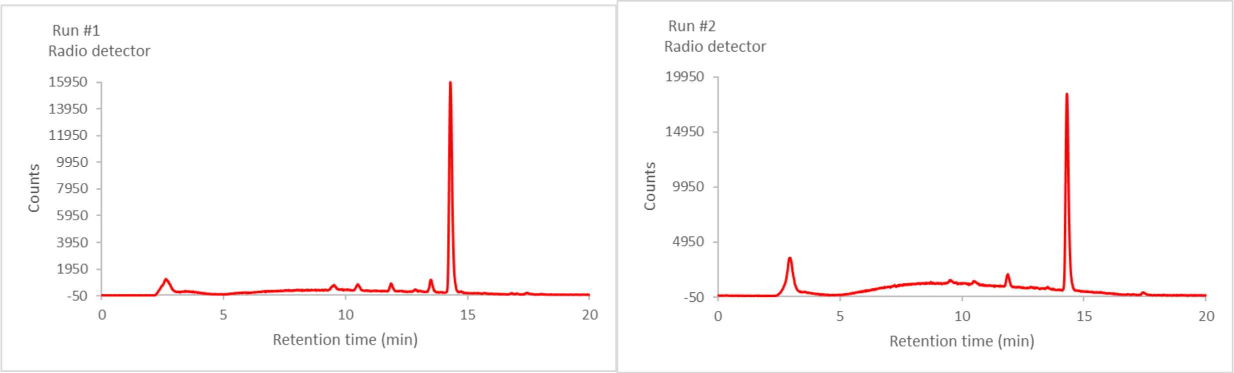

Figure S26. Crude radio-HPLC trace for [^{18}F]**2g** (HPLC column 2; HPLC gradient elution method A; retention time is 14.3 min)

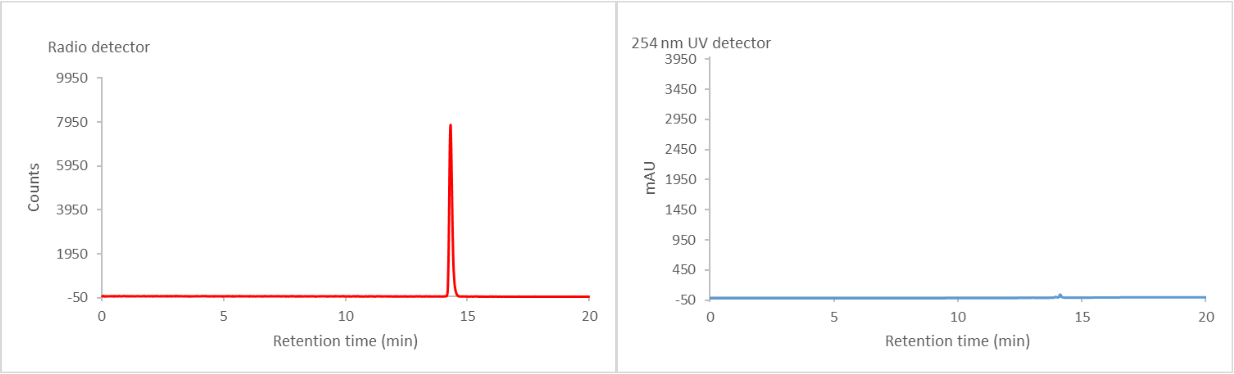

Figure S27. [^{18}F]**2g** QC (HPLC column 2; HPLC gradient elution method A)

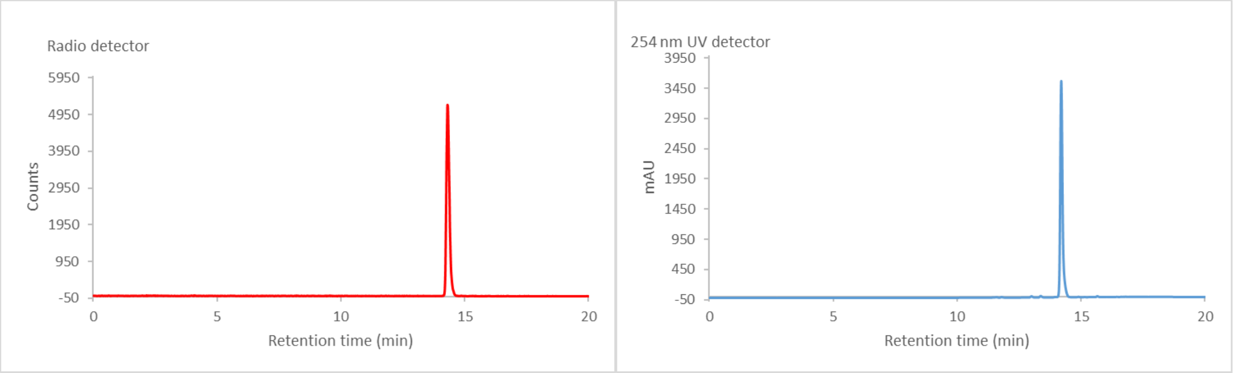

Figure S28. [^{18}F]**2g** co-injection (HPLC column 2; HPLC gradient elution method A)

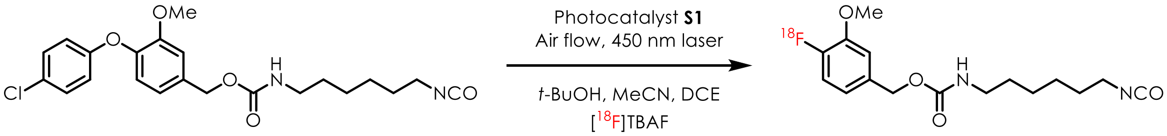

1h [^{18}**F**]**2h**

[^{18}**F**]**2h** was prepared from isocyanate precursor **1h** via general photoredox procedure (400 µL solvent mixture, no TBAB/MeCN, air flow and 20 min laser irradiation) in 28.5±3.6% (n=3) RCY.

Run #1: 32.5%

Run #2: 27.6%

Run #3: 25.4%

Average RCY: 28.5±3.6% (n=3)

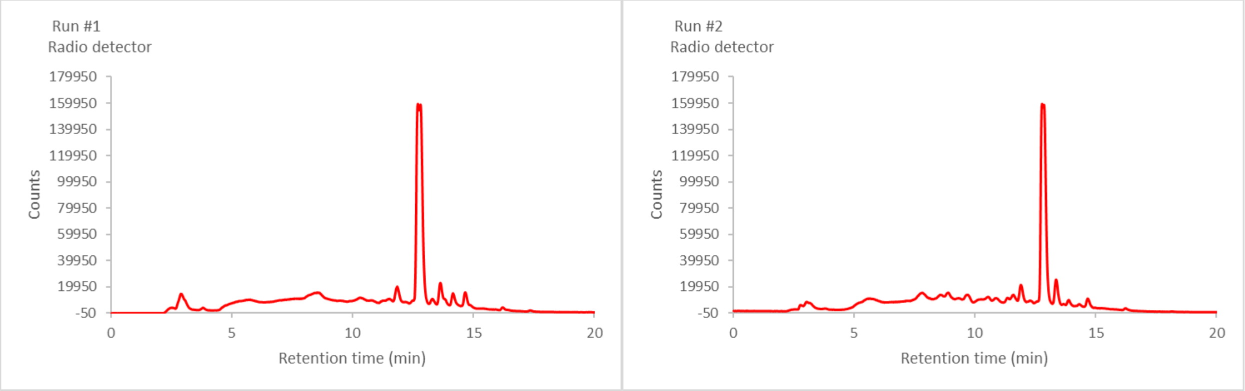

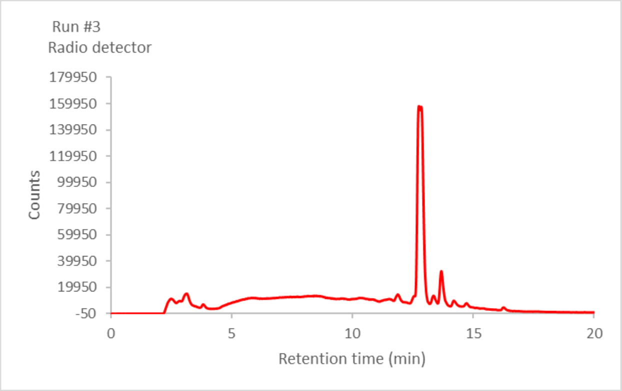

Figure S29. Crude radio-HPLC traces for [^{18}F]**2h** (HPLC column 2; HPLC gradient elution method A; retention time is 12.68 min)

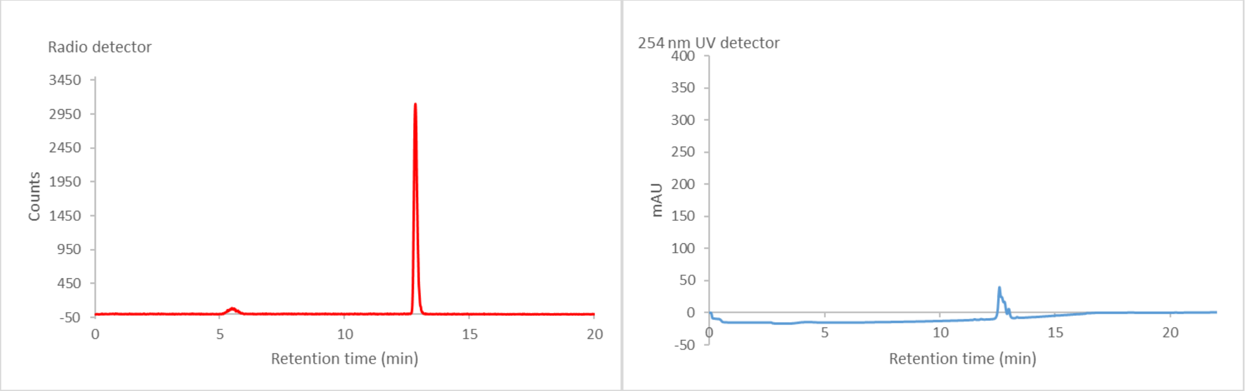

Figure S30. [^{18}F]**2h** QC (HPLC column 2; HPLC gradient elution method A)

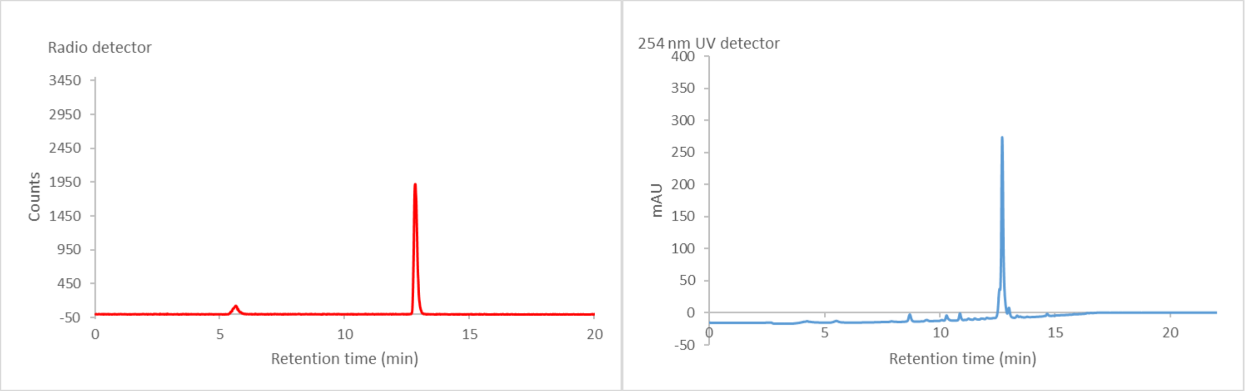

Figure S31. [^{18}F]**2h** co-injection (HPLC column 2; HPLC gradient elution method A)

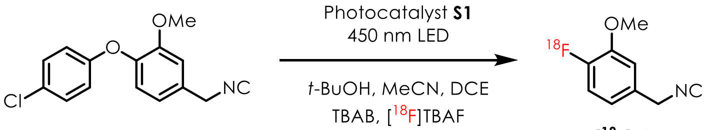

1i [^{18}F]**2i**

[^{18}**F**]**2i** was initially prepared from isocyanide precursor **1i** via general photoredox procedure (400 µL solvent mixture, 25 µL TBAB/MeCN, air flow and 20 min laser irradiation). It was observed that significant amounts of radioactivity evaporated along with the air flow.

[^{18}**F**]**2i** was then prepared via a revised procedure: isocyanide **1i** (4.5 mg, 0.016 mmol) was dissolved in solvent mixture (*t*-butyl alcohol:DCM:MeCN = 4:3:1, 133 µL) in a quartz tube opened to air. Photocatalyst **S1** (0.5 mg, 8.5 µmol), tetrabutylammonium bicarbonate solution (60 mg/mL solution in MeCN, 8.3 µL), and [^{18}F]TBAF/MeCN solution (1.7 mCi to 8.1 mCi) were added. The quartz tube was then sealed with a plastic screw cap, and irradiated with a 450 nm LED for 20 min. An aliquot of the resulting mixture was analyzed and purified by HPLC, affording the [^{18}**F**]**2i** in 4.7±0.4% RCY (n=3).

Run #1: 4.7%

Run #2: 5.0%

Run #3: 4.3%

Average RCY: 4.7±0.4% (n=3)

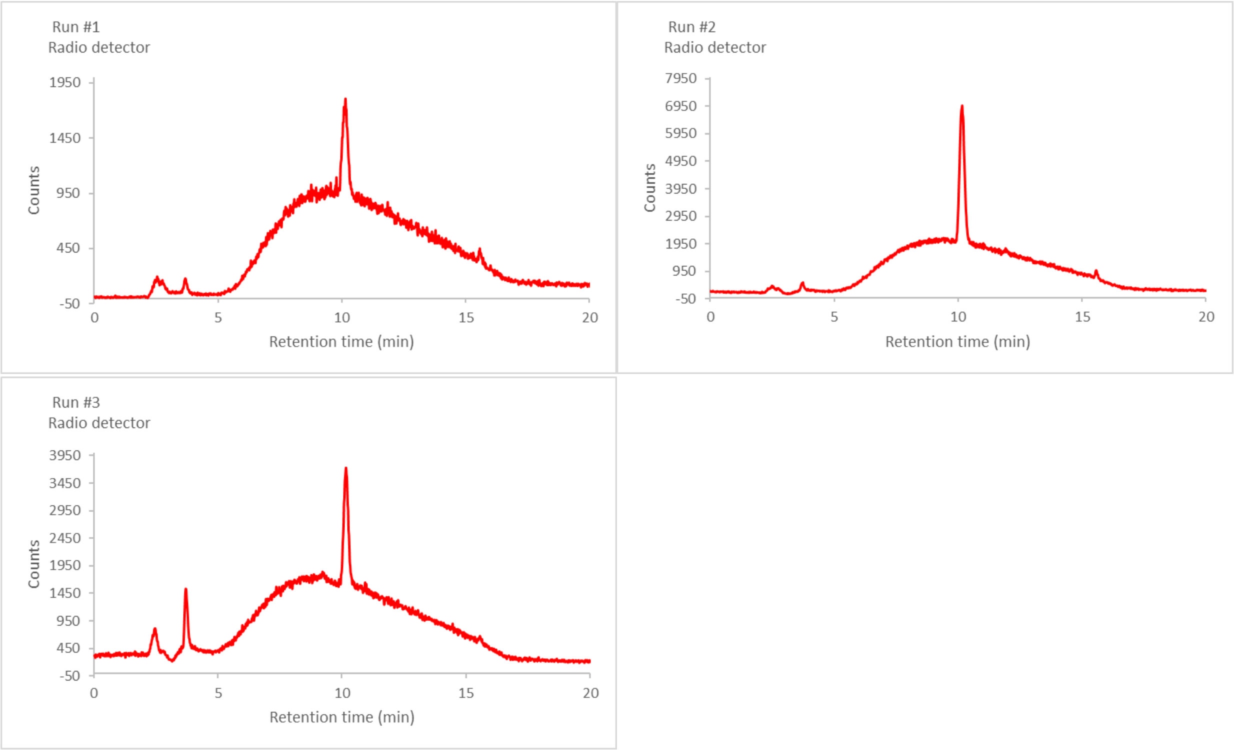

Figure S32. Crude radio-HPLC traces for [^{18}**F**]**2i** (HPLC column 2, HPLC gradient elution method A, retention time is 10.15 min)

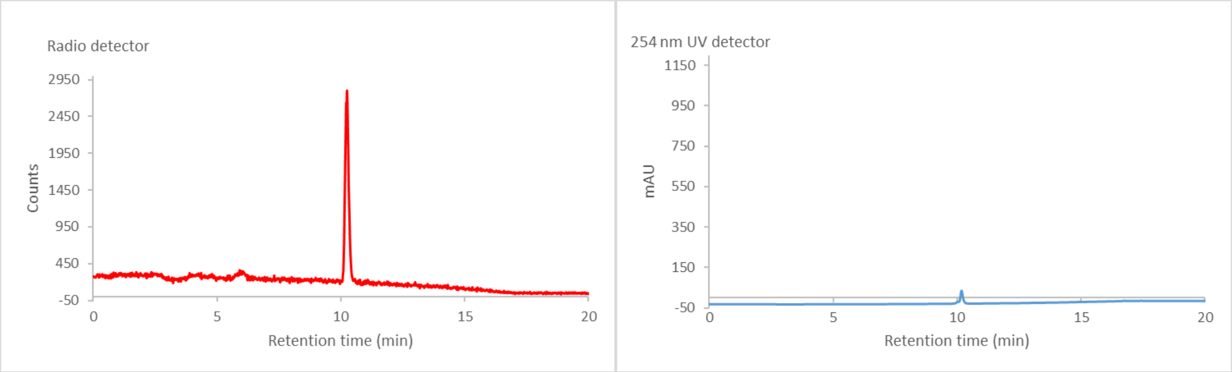

Figure S33. [^{18}F]**2i** QC (HPLC column 2, HPLC gradient elution method A)

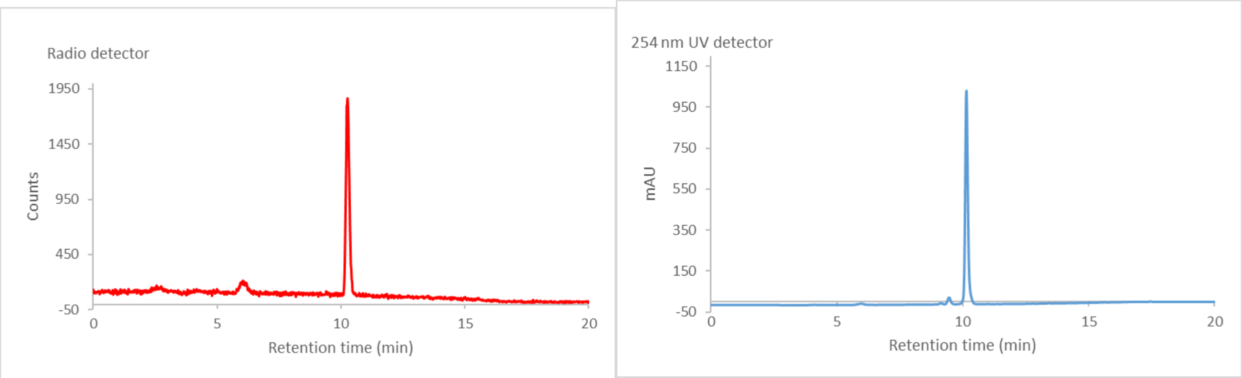

Figure S34. [^{18}F]**2i** co-injection (HPLC column 2, HPLC gradient elution method A)

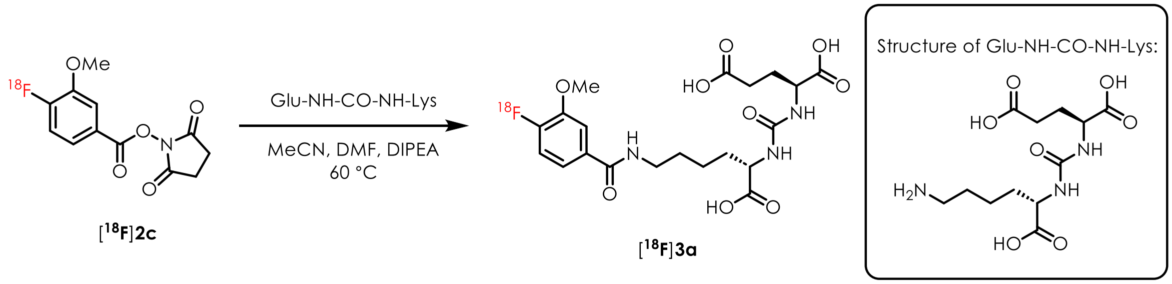

[^{18}F]**3a**: The HPLC purified [^{18}F]**2c** was concentrated to remove MeCN, water and TFA. Anhydrous MeCN was then added to redissolve [^{18}F]**2c**. To a solution of [^{18}F]**2c** (100 µCi to 400 µCi each experiment) in MeCN (200 µL) was added the Glu-NH-CO-NH-Lys (0.1 mg, in 10 µL DMF) and DIPEA (6 µL). The mixture was incubated at 60 °C and 550 rpm for 30 minutes. An aliquot of the mixture was then purified by HPLC.

Run #1: 24.6% RCY

Run #2: 28.9% RCY

Run #3: 48.6% RCY

Average RCY: 34.0±12.8% (n=3)

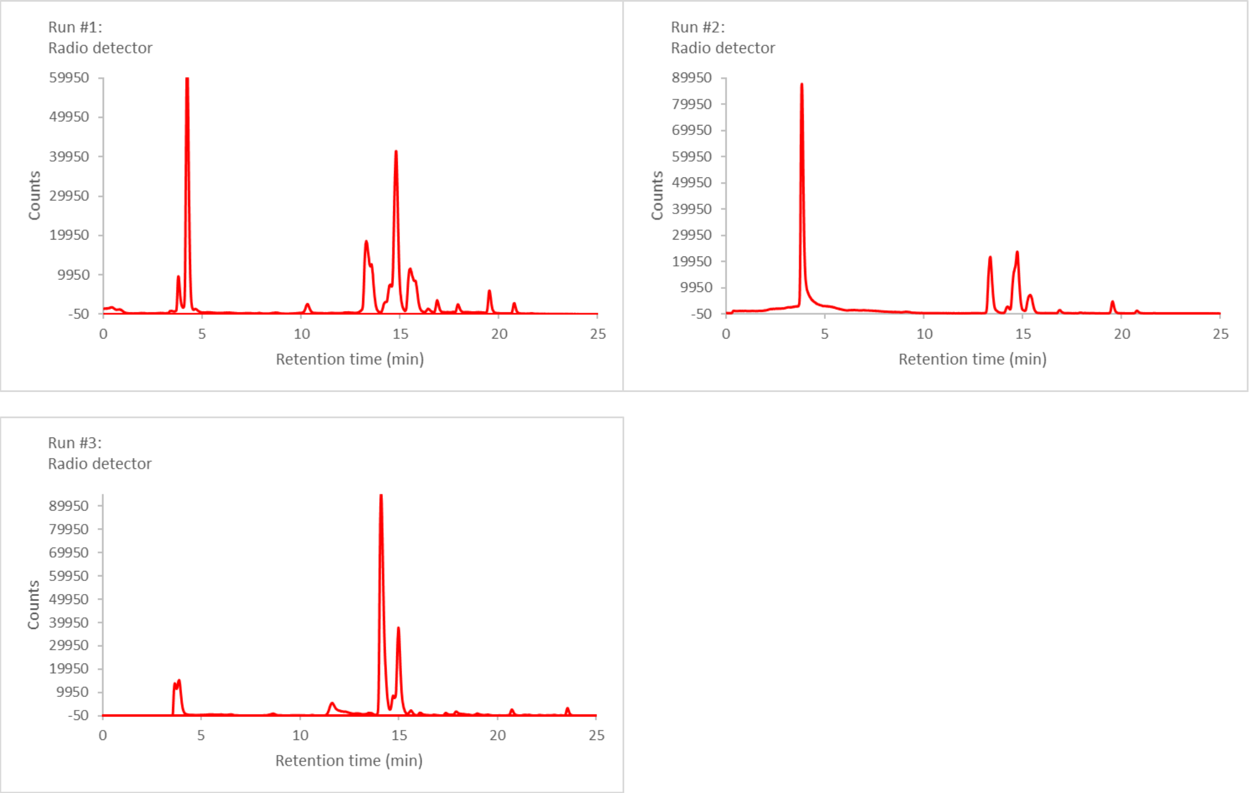

Figure S35. Crude radio-HPLC traces for [^{18}**F**]**3a** (HPLC column 2, HPLC gradient elution method B, retention time shifted between 13.28 min to 14.12 min)

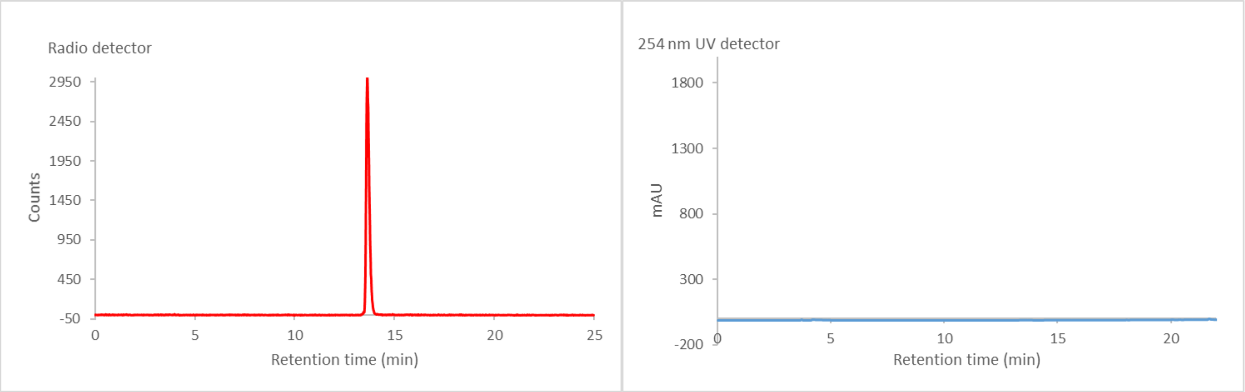

Figure S36. [^{18}**F**]**3a** QC (HPLC column 2, HPLC gradient elution method B)

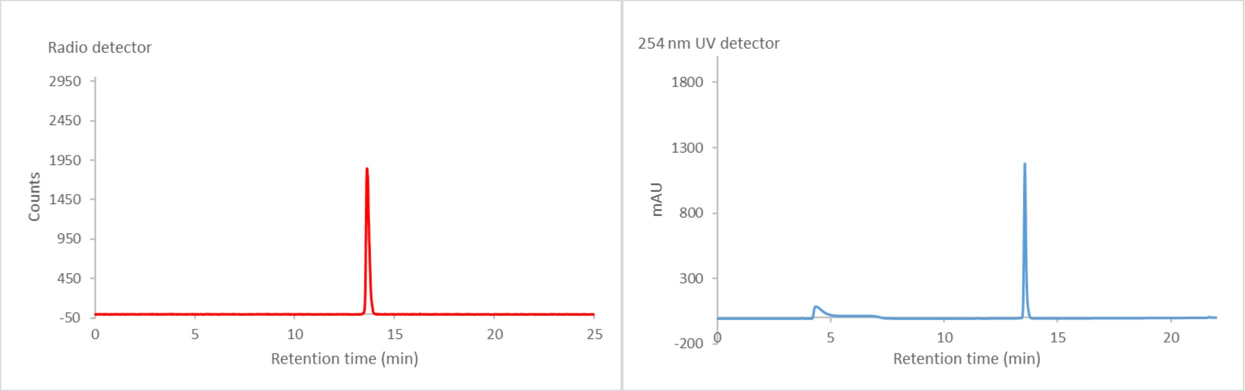

Figure S37. [^{18}F]**3a** co-injection (HPLC column 2, HPLC gradient elution method B)

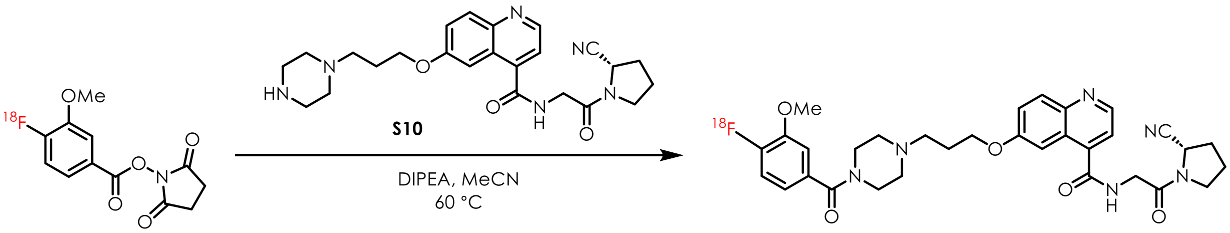

[^{18}F]**2c** [^{18}F]**3b**

[^{18}**F**]**3b**: The HPLC purified [^{18}**F**]**2c** was concentrated to remove MeCN, water and TFA. Anhydrous MeCN was then added to redissolve [^{18}**F**]**2c**. To a solution of [^{18}**F**]**2c** (100 µCi to 400 µCi each experiment) in MeCN (100 µL) was added the FAPI (0.41 mg, in 30 µL MeCN) and DIPEA (3 µL). The mixture was incubated at 60 °C and 550 rpm for 30 minutes. An aliquot of the mixture was then purified by HPLC.

Run #1: 39.4%

Run #2: 38.2%

Run #3: 22.8%

Average RCY: 33.5±9.2% (n=3)

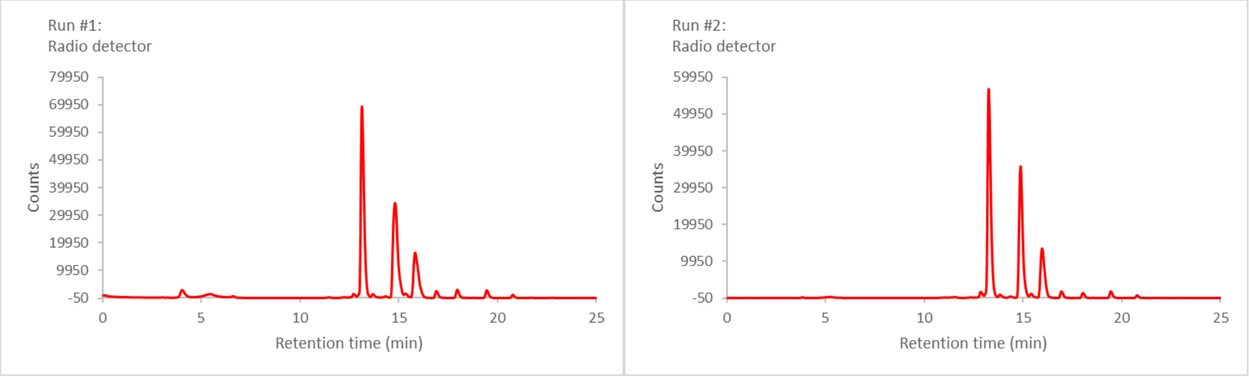

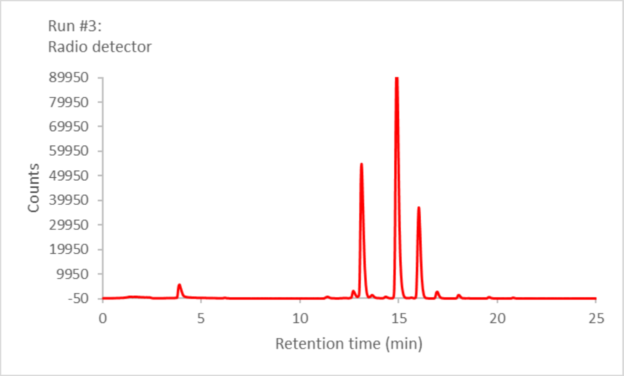

Figure S38. Crude radio-HPLC traces for [^{18}F]**3b** (HPLC column 2, HPLC gradient elution method B, retention time is 13.23 min)

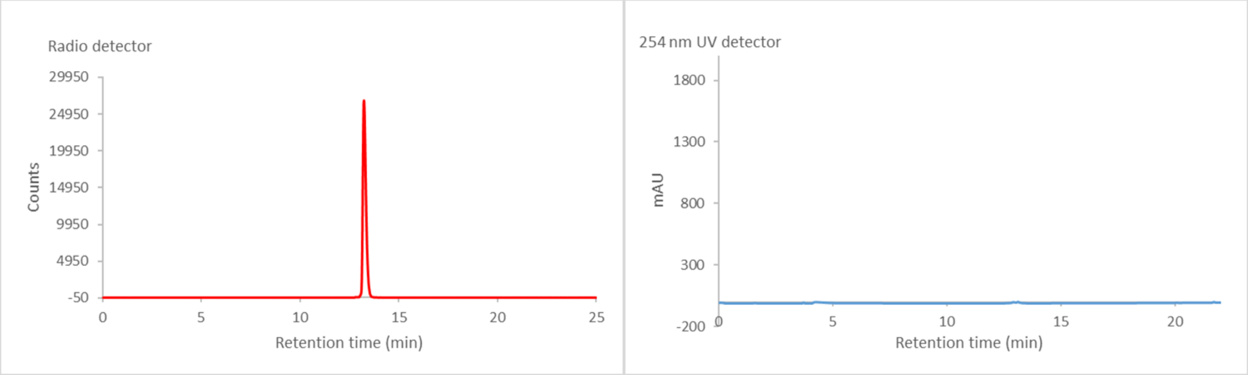

Figure S39. [^{18}F]**3b** QC (HPLC column 2, HPLC gradient elution method B)

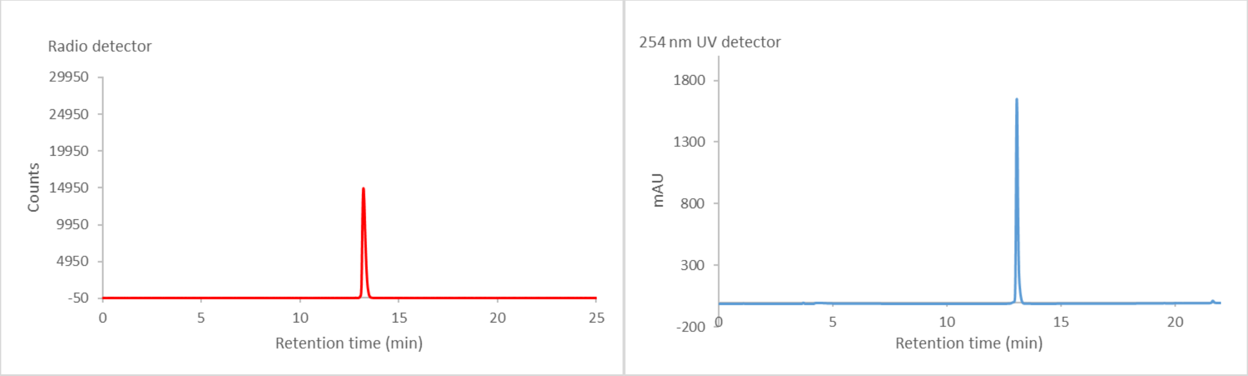

Figure S40. [^{18}F]**3b** co-injection (HPLC column 2, HPLC gradient elution method B)

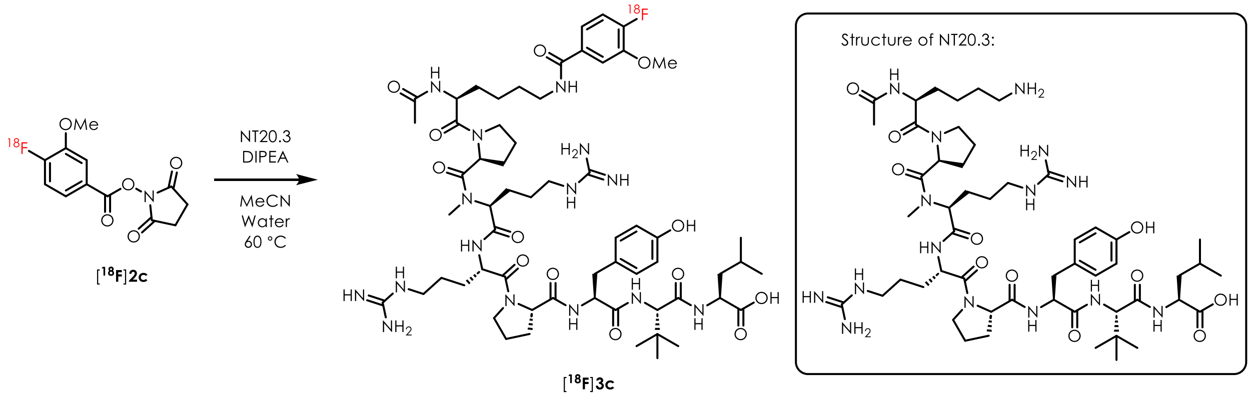

[^{18}F]**3c**: The HPLC purified [^{18}F]**2c** was concentrated to remove MeCN, water and TFA. Anhydrous MeCN was then added to redissolve [^{18}F]**2c**. To a solution of [^{18}F]**2c** (197 µCi) in MeCN (100 µL) was added the NT20.3 peptide (0.4 mg, in 40 µL 1:1 MeCN:water) and DIPEA (3 µL). The mixture was incubated at 60 °C and 550 rpm for 30 minutes. An aliquot of the mixture was then purified by HPLC.

Run #1: 77.0% RCY (n=1)

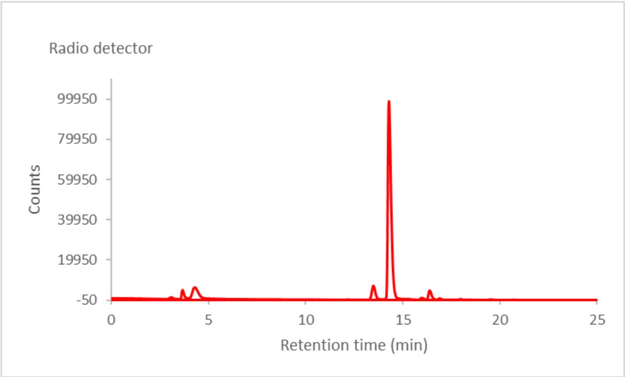

Figure S41. Crude radio-HPLC trace for [^{18}F]**3c** (HPLC column 2, HPLC gradient elution method B, retention time is 14.28 min)

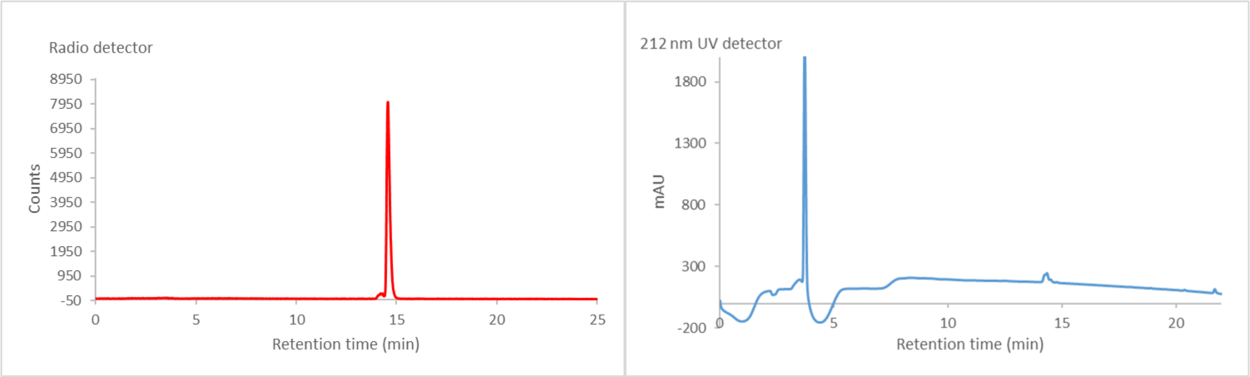

Figure S42. [^{18}F]**3c** QC (HPLC column 2, HPLC gradient elution method B)

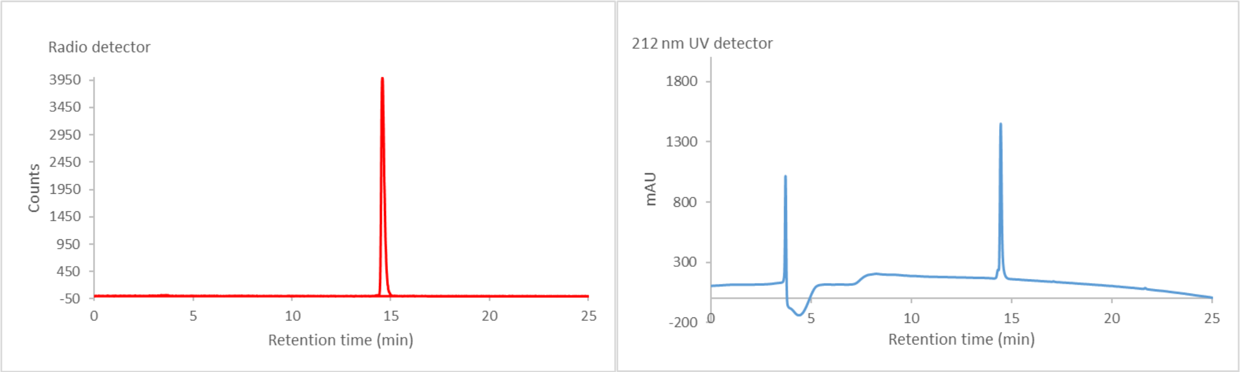

Figure S43. [^{18}F]**3c** co-injection (HPLC column 2, HPLC gradient elution method B)

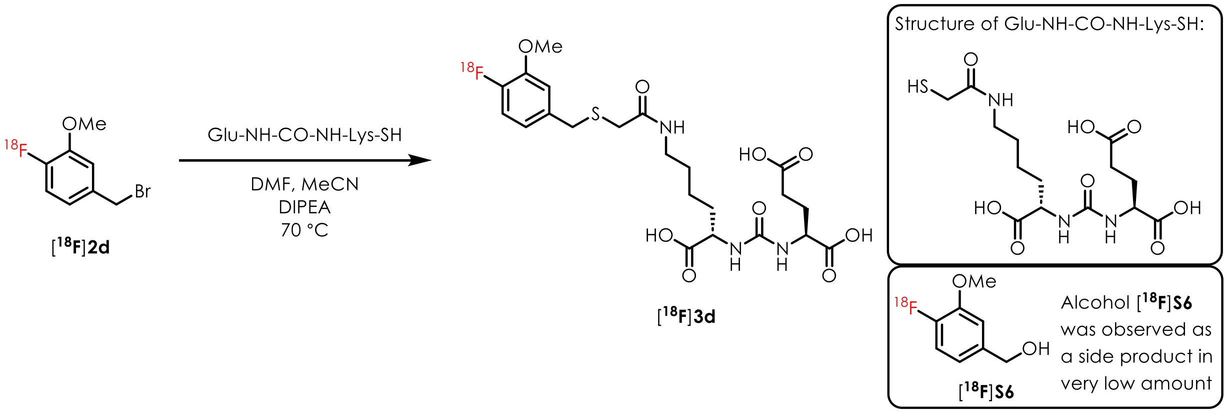

[^{18}F]**3d** preparation via water-free condition: HPLC isolated [^{18}F]**2d** (in MeCN/water mixture) was loaded into a C18 cartridge. A large amount of air was passed through the C18 cartridge to remove residue water. [^{18}F]**2d** was then eluted from the C18 cartridge with anhydrous MeCN. A fraction of this [^{18}F]**2d** MeCN solution (100 µL) was mixed with Glu-NH-CO-NH-Lys-SH stock solution (0.1 mg, 0.25 µmol, 3.3 µL 0.078 M stock solution in DMF) in a microcentrifuge tube. DIPEA (2 µL) was added last, and the mixture was incubated at 70 °C and 300 rpm. After 30 min, HPLC purification afforded [^{18}F]**3d** in 75.4% RCY.

Note: Run #1 and #2 were purified by HPLC gradient elution method B, which cannot separate [^{18}F]**3d** and [^{18}F]**S6**. Separate HPLC analyses were done for the purified material from run #1 and #2 with HPLC gradient elution method C, and [^{18}F]**S6** was found in very small amount (7.1% and 8.1%). Crude from run #3 was purified by HPLC gradient elution method C, [^{18}F]**S6** was found in trace amount.

Run #1: 75.6%

Run #2: 75.9%

Run #3: 74.8%

Average RCY: 75.4±0.6% (n=3)

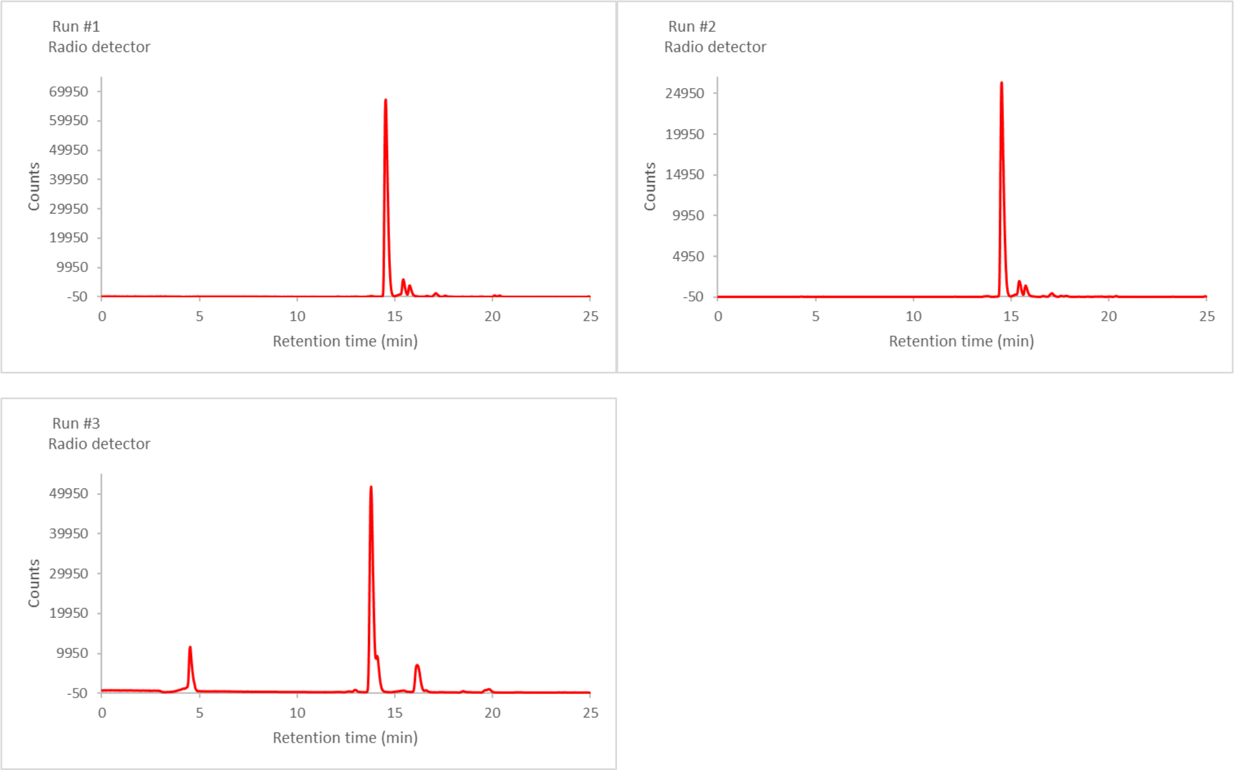

Figure S44. Crude radio-HPLC traces for [^{18}F]**3d** (Run #1 and #2 used HPLC column 2, HPLC gradient elution method B, retention time for [^{18}F]**3d** is 14.55 min; run #3 used HPLC column 2, HPLC gradient elution method C, retention time for [^{18}F]**3d** is 13.80 min, retention time for [^{18}F]**S6** is 13:00 min)

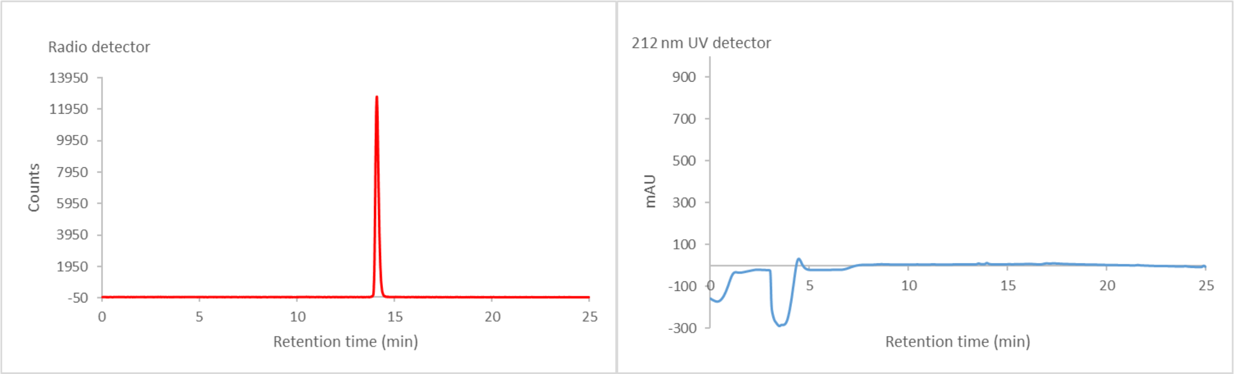

Figure S45. [^{18}F]**3d** QC (HPLC column 2, HPLC gradient elution method C)

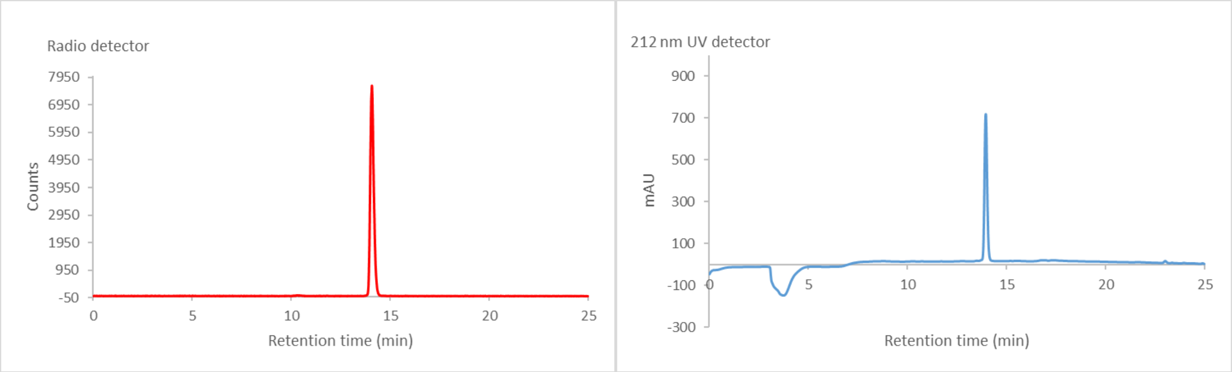

Figure S46. [^{18}F]**3d** co-injection (HPLC column 2, HPLC gradient elution method C)

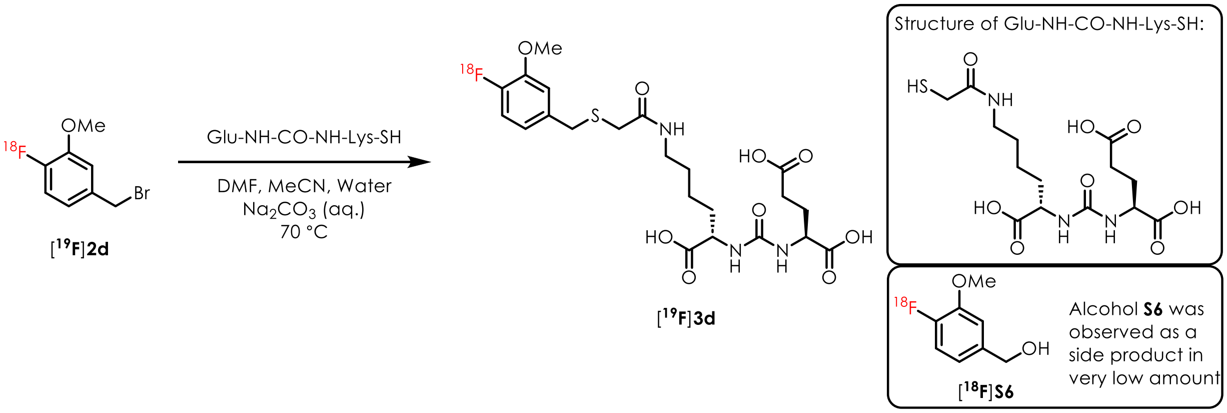

[^{18}F]**3d** preparation via aqueous condition: HPLC isolated [^{18}F]**2d** (100 µL, in MeCN/water mixture) was mixed with Glu-NH-CO-NH-Lys-SH stock solution (0.1 mg, 0.25 µmol, 3.3 µL 0.078 M stock solution in DMF) in a microcentrifuge tube. Sodium carbonate buffer solution (50 µL, 0.5 M in water) was then added. The mixture was incubated at 70 °C and 300 rpm. After 30 min, HPLC purification afforded [^{18}F]**3d** in 72.8% RCY.

Run #1: 74.1%

Run #2: 71.4%

Average RCY: 72.8±1.9% (n=2)

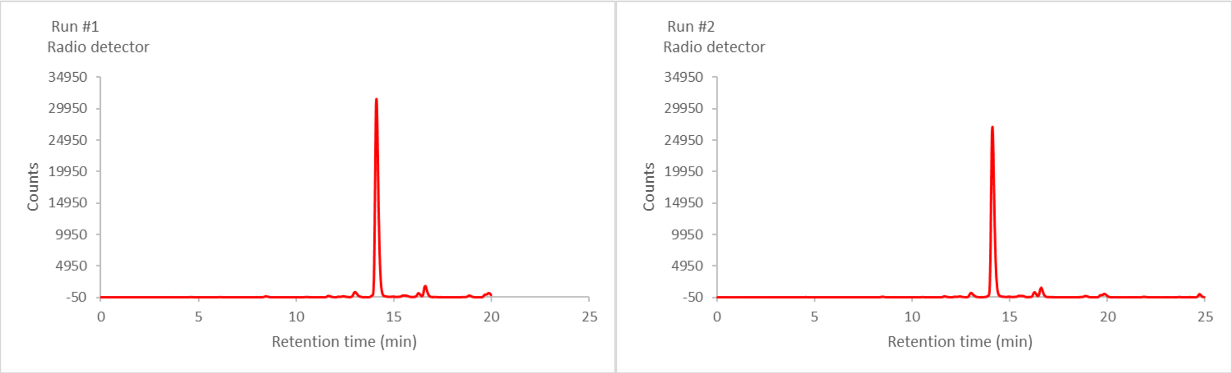

Figure S47. Crude radio-HPLC traces for [^{18}F]**3d** (HPLC column 2, HPLC gradient elution method C)

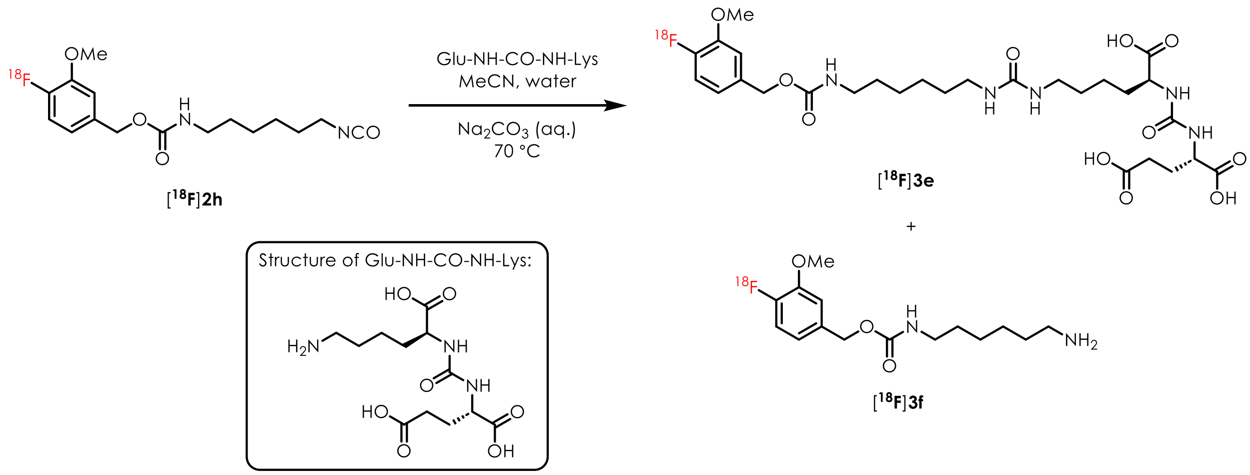

[^{18}F]**3e**: To a solution of Glu-NH-CO-NH-Lys (0.5 mg, 1.6 μmol) in MeCN (10 μL) and water (10 μL) was added sodium carbonate aqueous buffer solution (0.5 M in water, 100 μL). The HPLC purified [^{18}F]**2h** (100 μL, 84 μCi to 192 μCi activity) was then added. The mixture was incubated at 70 °C and 400 rpm. After 30 min, the mixture was purified by HPLC. The RCY of [^{18}F]**3e** was calculated based on the amount of radioactive material isolated, the RCC of [^{18}F]**3f** was estimated based on the HPLC radio trace peak integration ratio (between [^{18}F]**3e** and [^{18}F]**3f**) and the isolated RCY of [^{18}F]**3e**.

[^{18}F]**3e** RCY #1: 28.4% estimated [^{18}F]**3f** RCC #1: 43.4%

[^{18}F]**3e** RCY #2: 31.3% estimated [^{18}F]**3f** RCC #2: 47.0%

[^{18}F]**3e** RCY #3: 22.3% estimated [^{18}F]**3f** RCC #3: 47.5%

Average RCY of [^{18}F]**3e**: 27.3±4.6% (n=3)

Average estimated RCY of [^{18}F]**3f**: 46.0±2.2% (n=3)

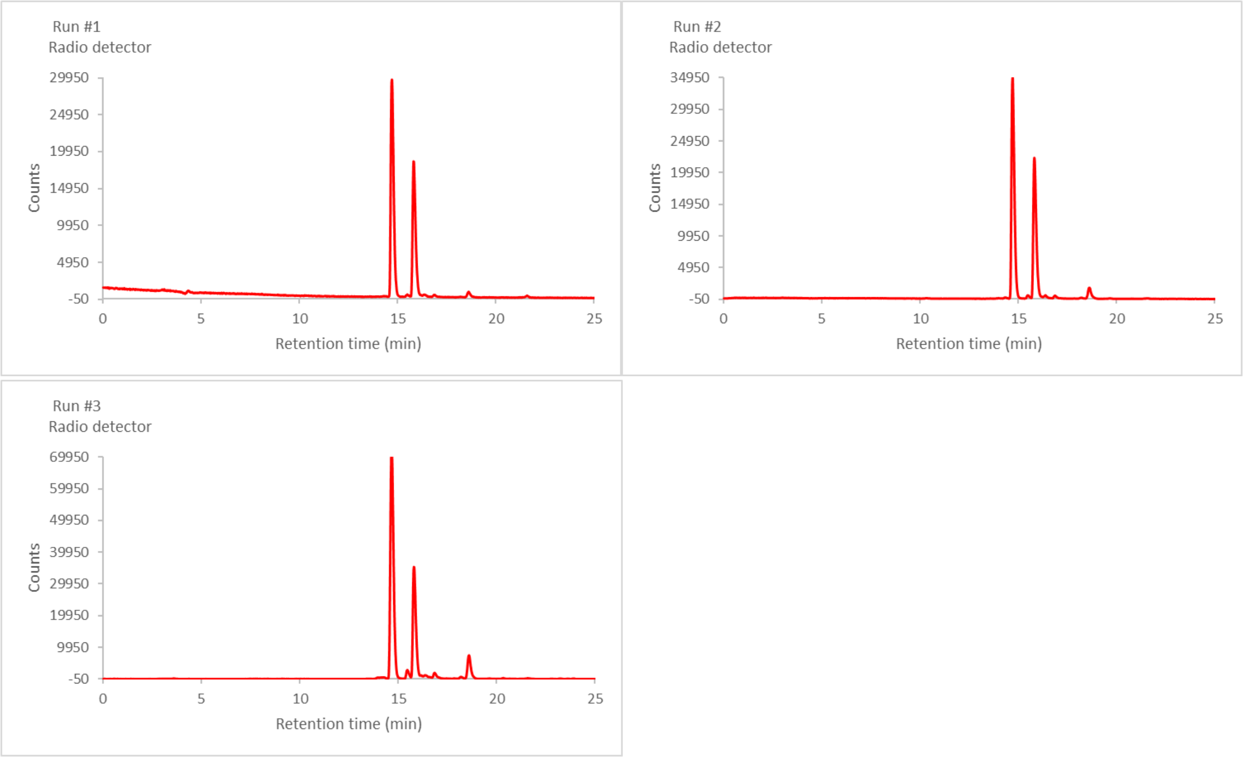

Figure S48. Crude radio-HPLC traces for [^{18}F]**3e** (HPLC column 2, HPLC gradient elution method B, retention time for [^{18}F]**3e** is 15.80 min, retention time for [^{18}F]**3f** is 14.70 min)

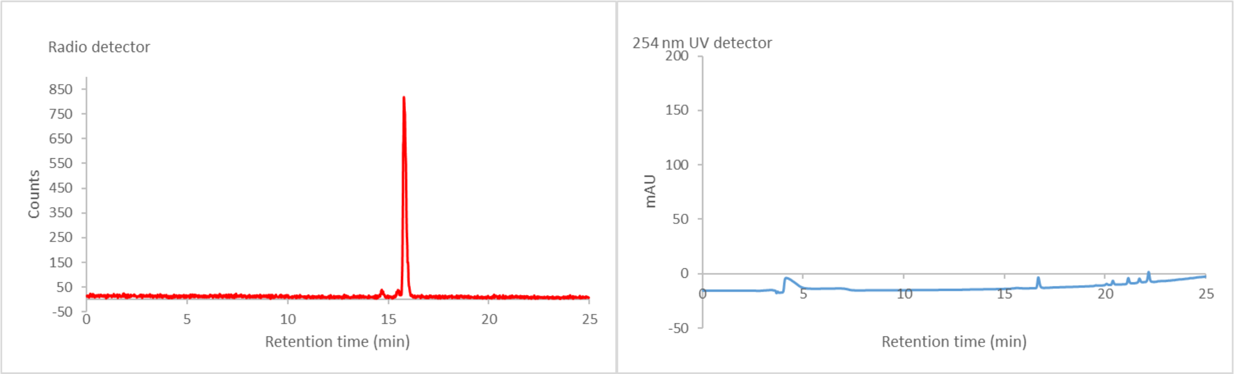

Figure S49. [^{18}F]**3e** QC (HPLC column 2, HPLC gradient elution method B)

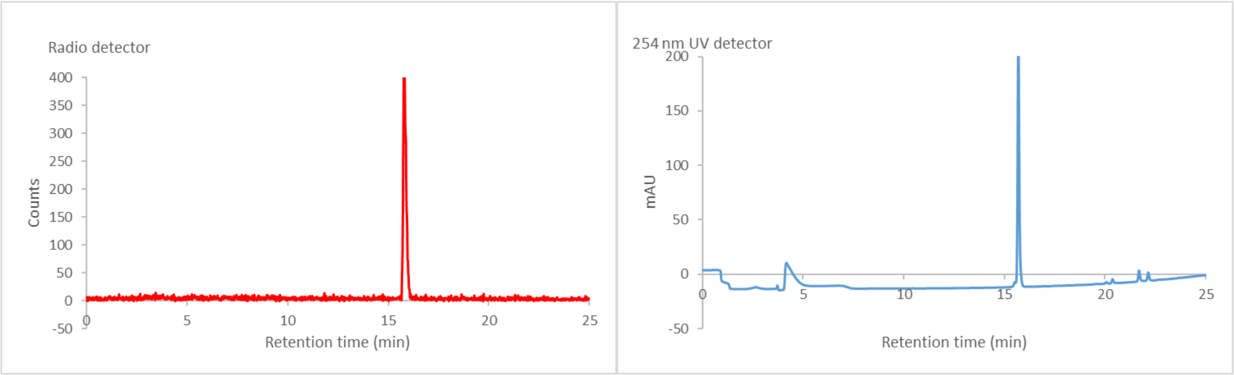

Figure S50. [^{18}**F**]**3e** co-injection (HPLC column 2, HPLC gradient elution method B)

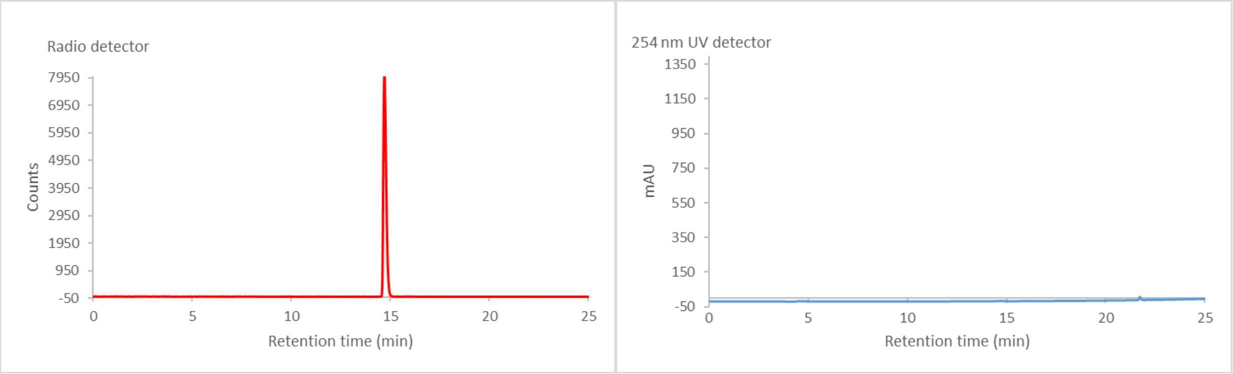

Figure S51. [^{18}**F**]**3f** QC (HPLC column 2, HPLC gradient elution method B)

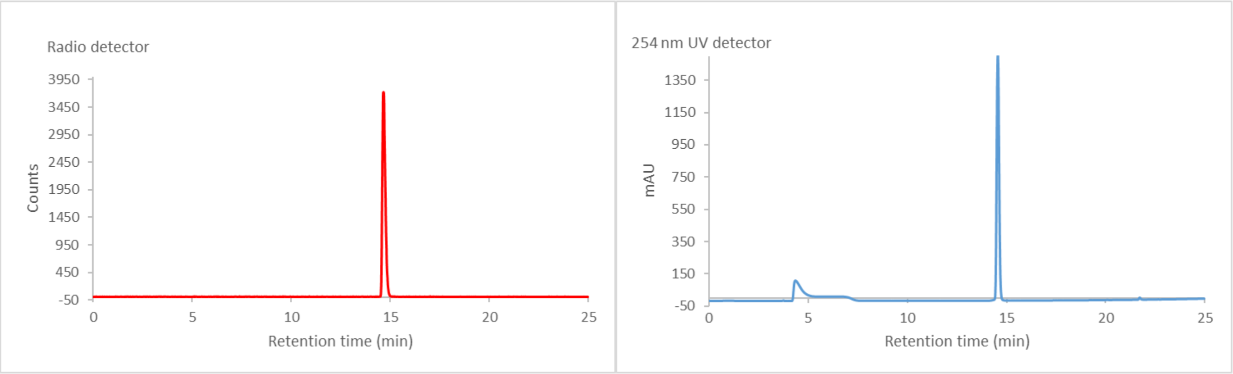

Figure S52. [^{18}**F**]**3f** co-injection (HPLC column 2, HPLC gradient elution method B)

Small animal PET imaging study

The human prostate cancer cell line with high PSMA expression, PC3-PSMA, was obtained from the Tissue Culture Facility, UNC Lineberger Comprehensive Cancer Center. PC3-PSMA cells were cultured in DMEM medium supplemented with 10% FBS and 100 U/ml of penicillin and 100 µg/ml streptomycin in a humidified atmosphere of 5% CO_2 at 37°C. Nude mice were obtained from the Animal Study Facility of UNC Chapel Hill. When the mice were 4-6 weeks old, about 2X106 PC3-PSMA cells per 0.1mL were injected subcutaneously in the right shoulder of nude mice for tumor xenograft. When tumors reached 200 mm^3 in size, mice were used for imaging studies. All animal procedures were approved by the University of North Carolina Institutional Animal Care and Use Committee.

Static PET and CT images were acquired at 0.5h, 1.5h, and 3h post-injection for [^{18}**F**]**3a** under isoflurane anesthesia for 15 min. About 1 MBq of [^{18}**F**]**3a** were injected to the PC3-PSMA xenograft mouse (n=4) intravenously. The mice were awakened between imaging times. List-mode data were collected and reconstructed with the algorithm described before.[5] The regions

of interest (ROIs) were drawn using AMIDE software on coronal PET/CT images. List-mode data were collected and reconstructed with the algorithm described before.[5] The regions of interest (ROIs) were drawn using AMIDE software on coronal PET/CT images. Organ and tumor uptake are expressed as mean ± standard deviation (SD) percentage injected dose per gram (%ID/g) after correcting for radioactivity decay.

Appendix: NMR

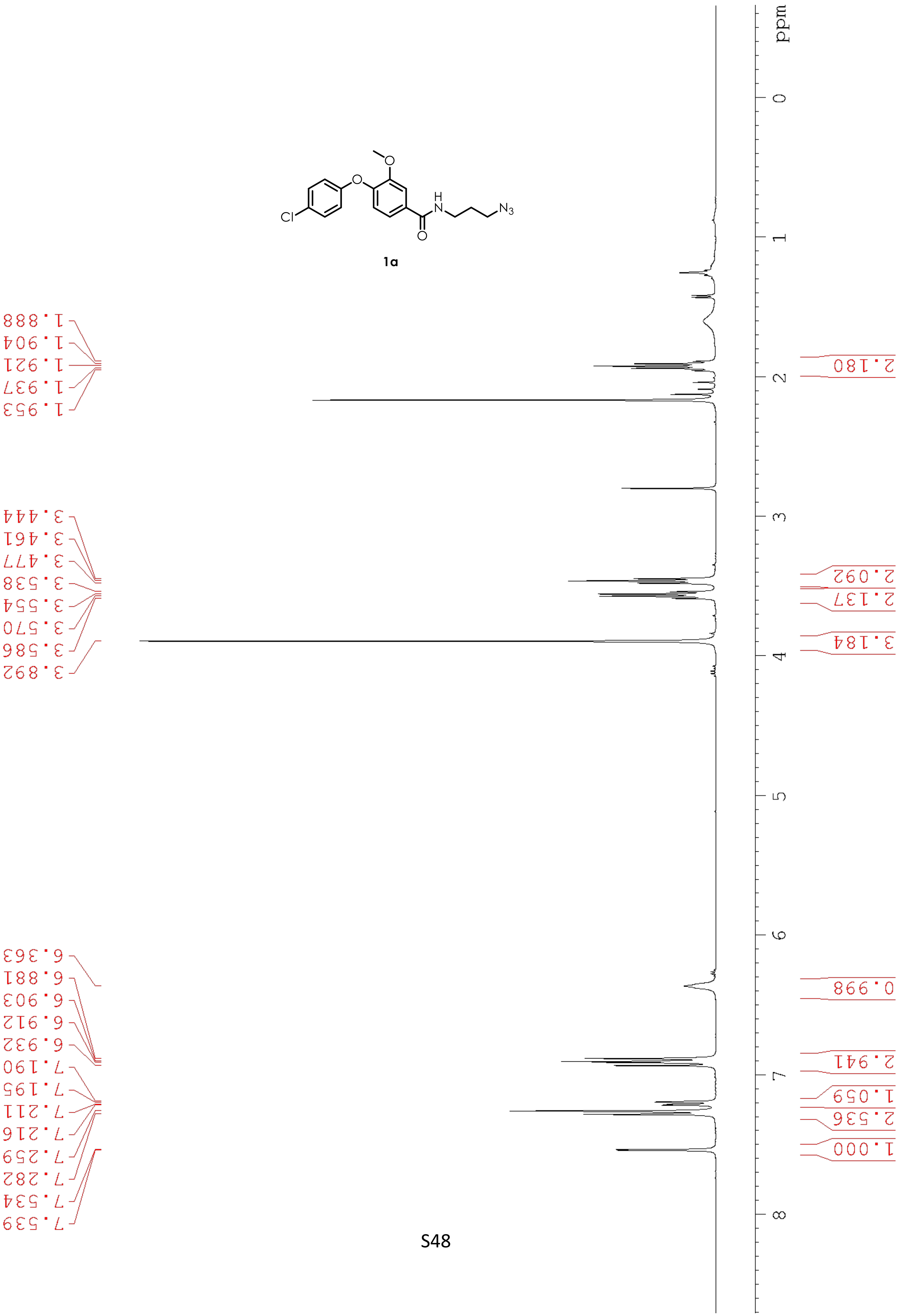
1a
Cl
O
H
N
N3
ppm
1.888
1.904
1.921
1.937
1.953
3.444
3.461
3.477
3.538
3.554
3.570
3.586
3.892
6.363
6.881
6.903
6.912
6.932
7.190
7.195
7.211
7.216
7.259
7.282
7.534
7.539
2.180
2.092
2.137
3.184
0.998
2.941
1.059
2.536
1.000

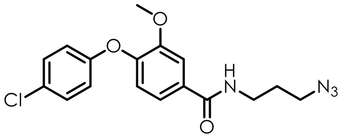
Cl
O
O
H
N
N3

1a

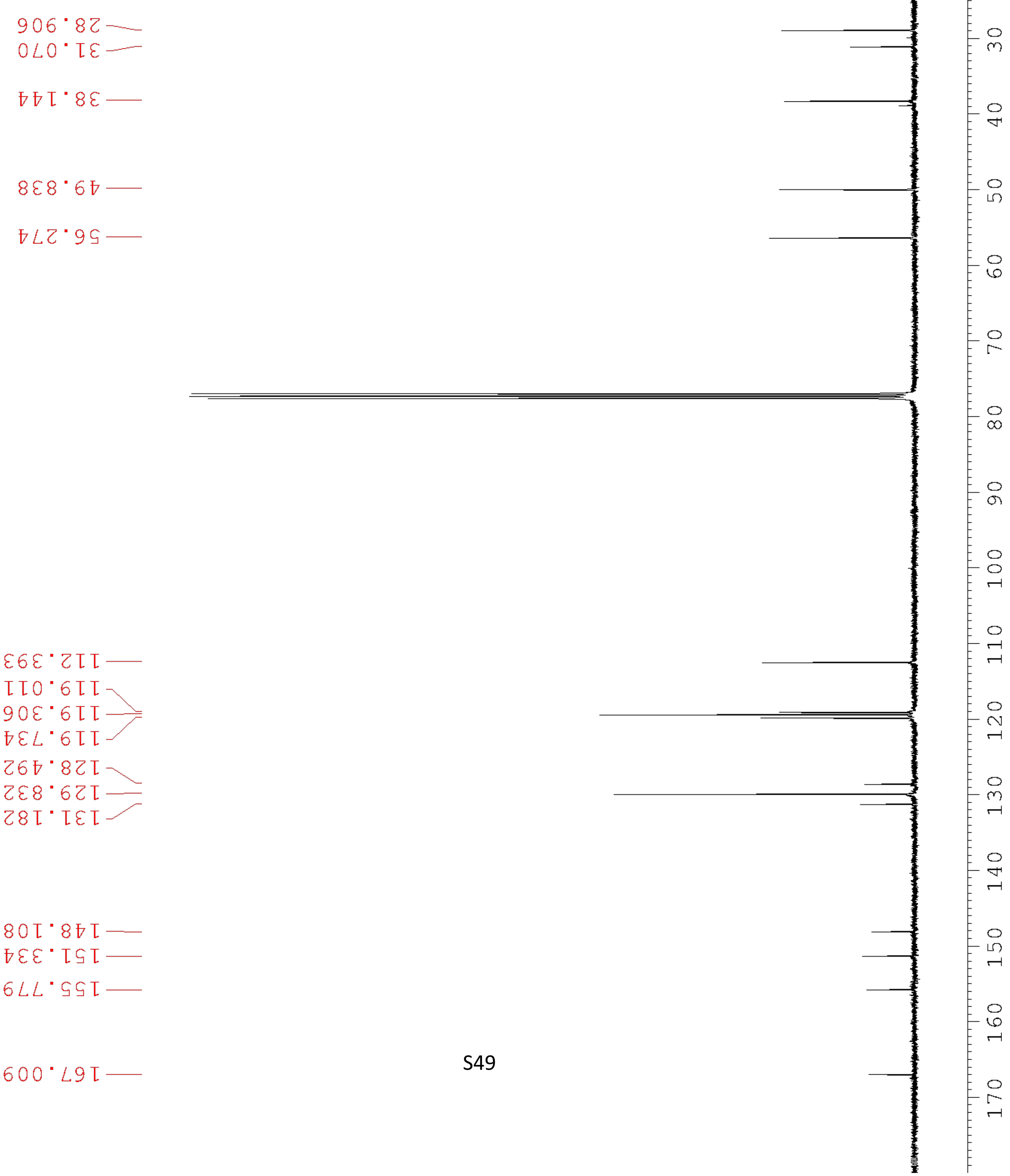
28.906
31.070
38.144
49.838
56.274
112.393
119.011
119.306
119.734
128.492
129.832
131.182
148.108
151.334
155.779
167.009
ppm

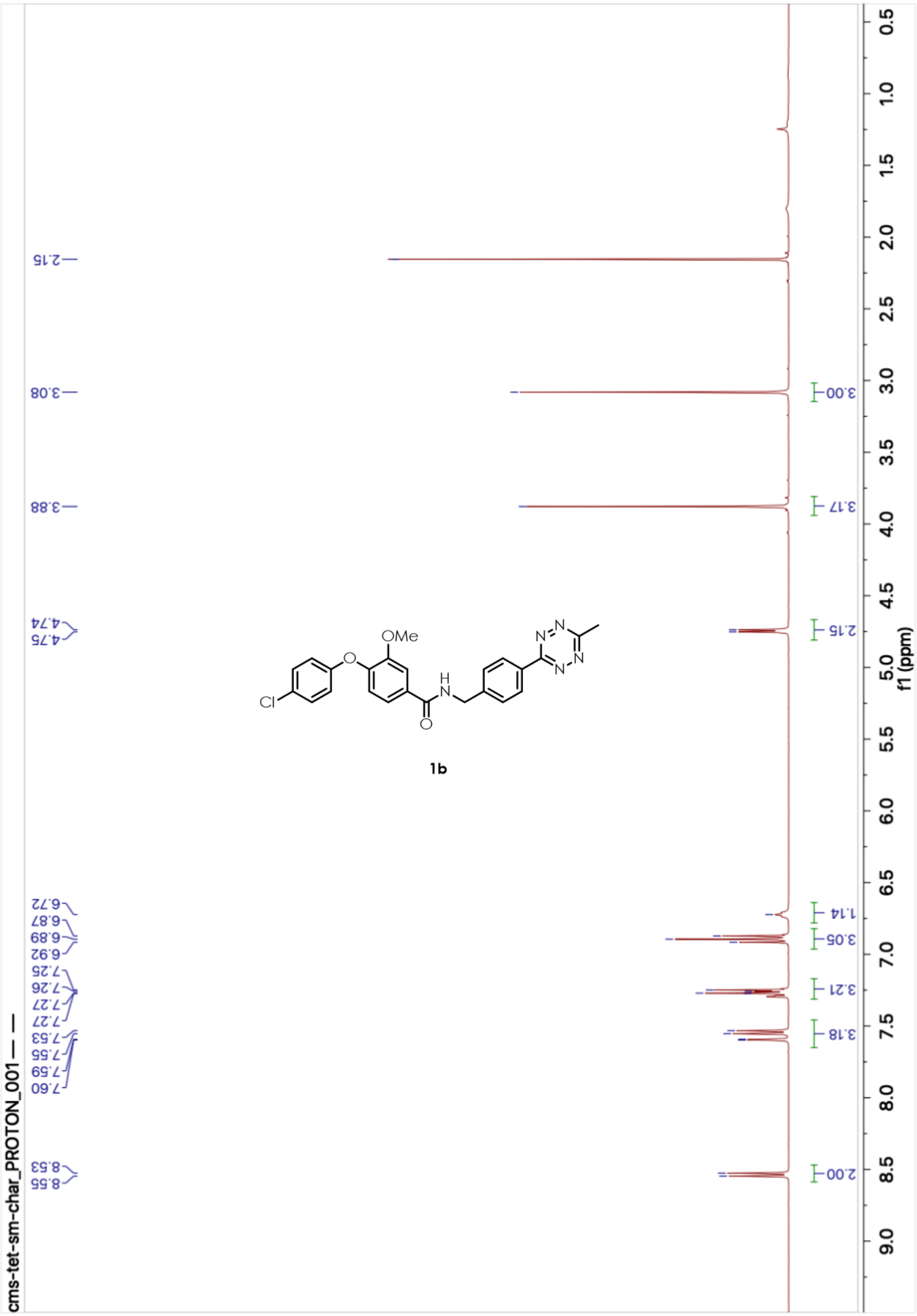
cms-tet-sm-char_PROTON_001
2.15
3.08
3.88
4.74
4.75
6.72
6.87
6.89
6.92
7.25
7.26
7.27
7.27
7.53
7.55
7.59
7.60
8.53
8.55
3.00
3.17
2.15
1.14
3.05
3.21
3.18
2.00
OMe
O
Cl
H
N
O
N
N
N
N
1b
f1 (ppm)

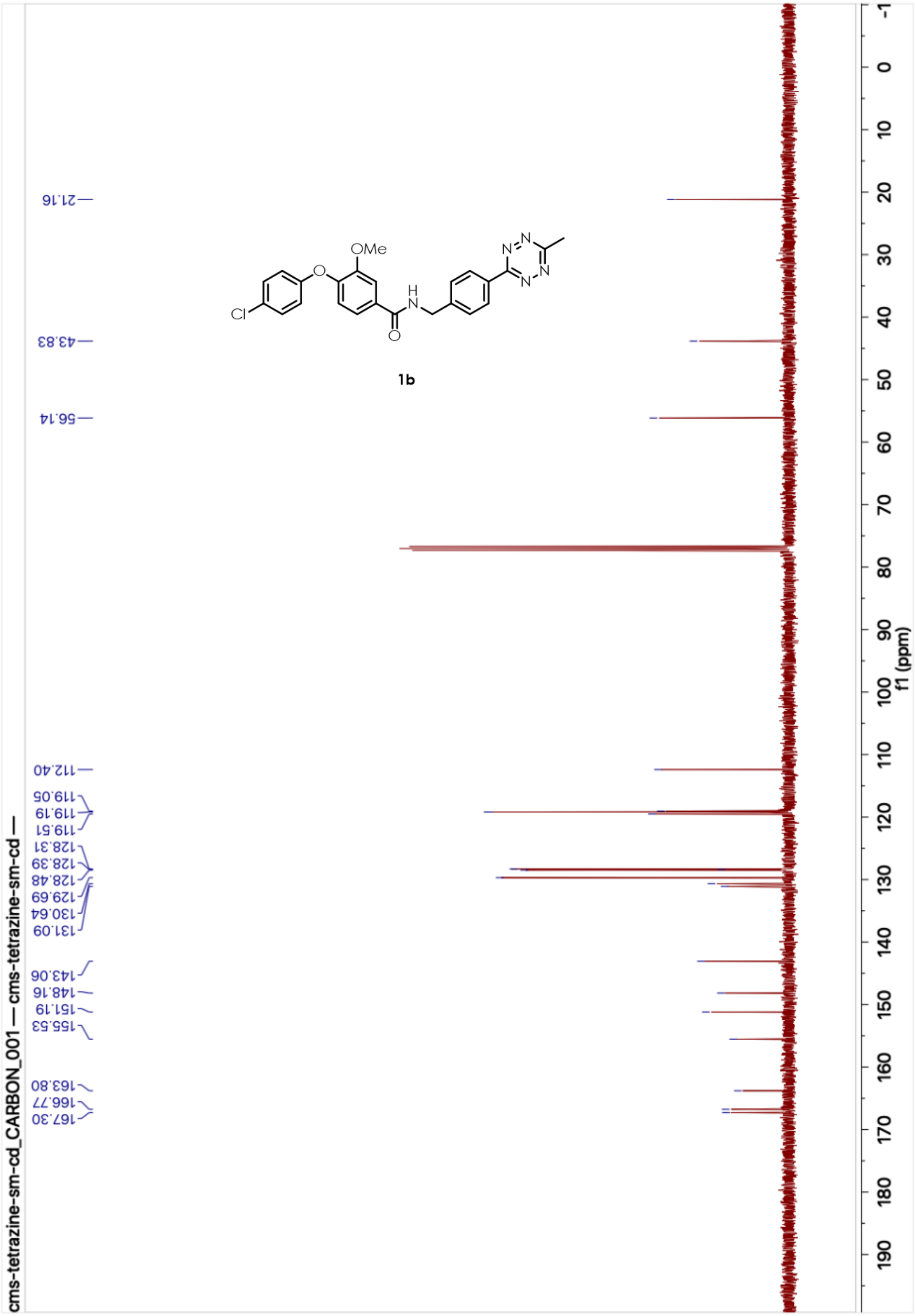
cms-tetrazine-sm-cd_CARBON_001 — cms-tetrazine-sm-cd —
21.16
43.83
56.14
112.40
119.05
119.19
119.51
128.31
128.39
128.48
129.69
130.64
131.09
143.06
148.16
151.19
155.53
163.80
166.77
167.30
OMe
Cl
O
H
N
N
1b
f1 (ppm)

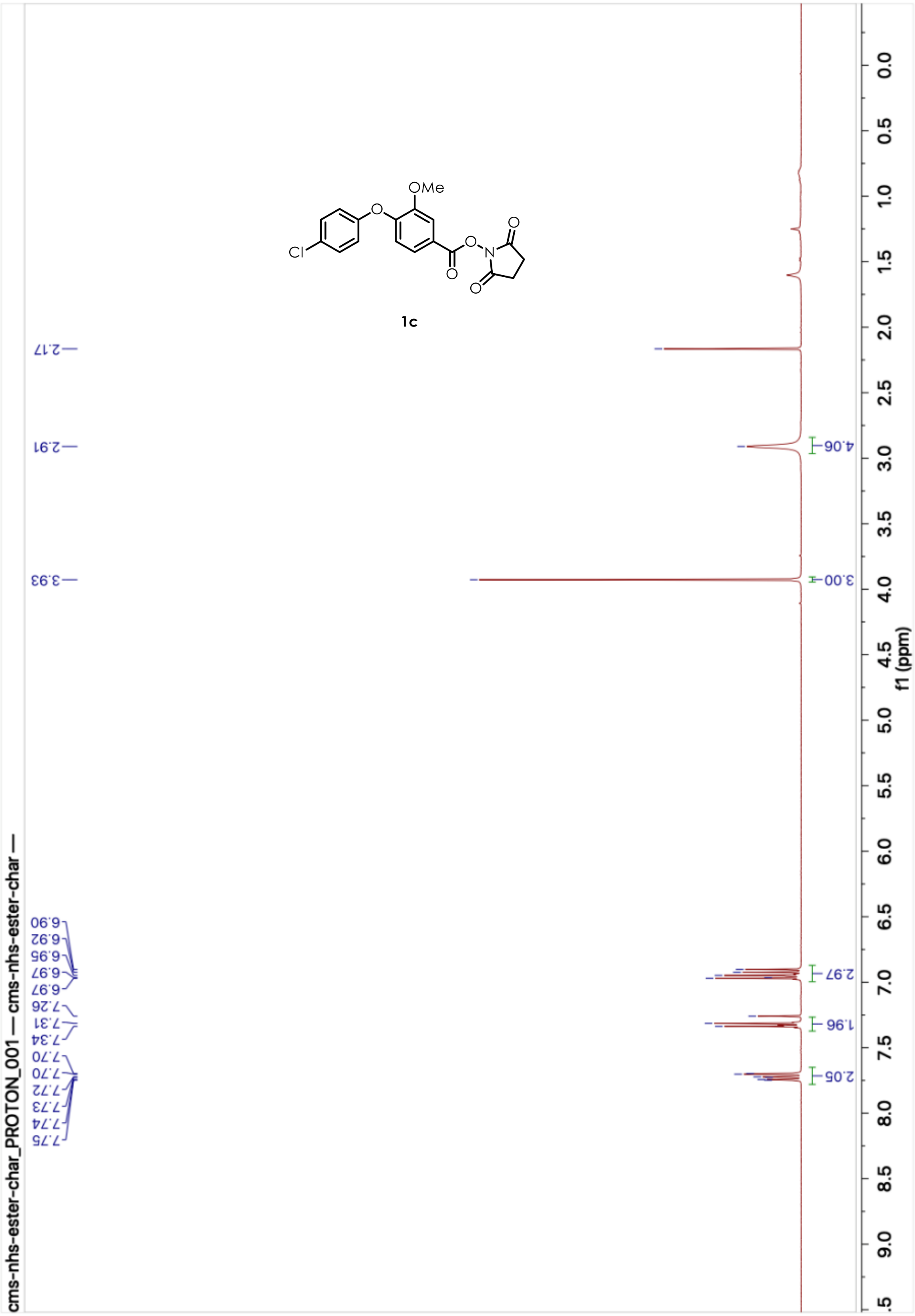
cms-nhs-ester-char_PROTON_001 — cms-nhs-ester-char —
7.75
7.74
7.73
7.72
7.70
7.70
7.34
7.31
7.26
6.97
6.97
6.95
6.92
6.90
3.93
2.91
2.17
OMe
Cl
O
N
1c
2.05
1.96
2.97
3.00
4.06
f1 (ppm)

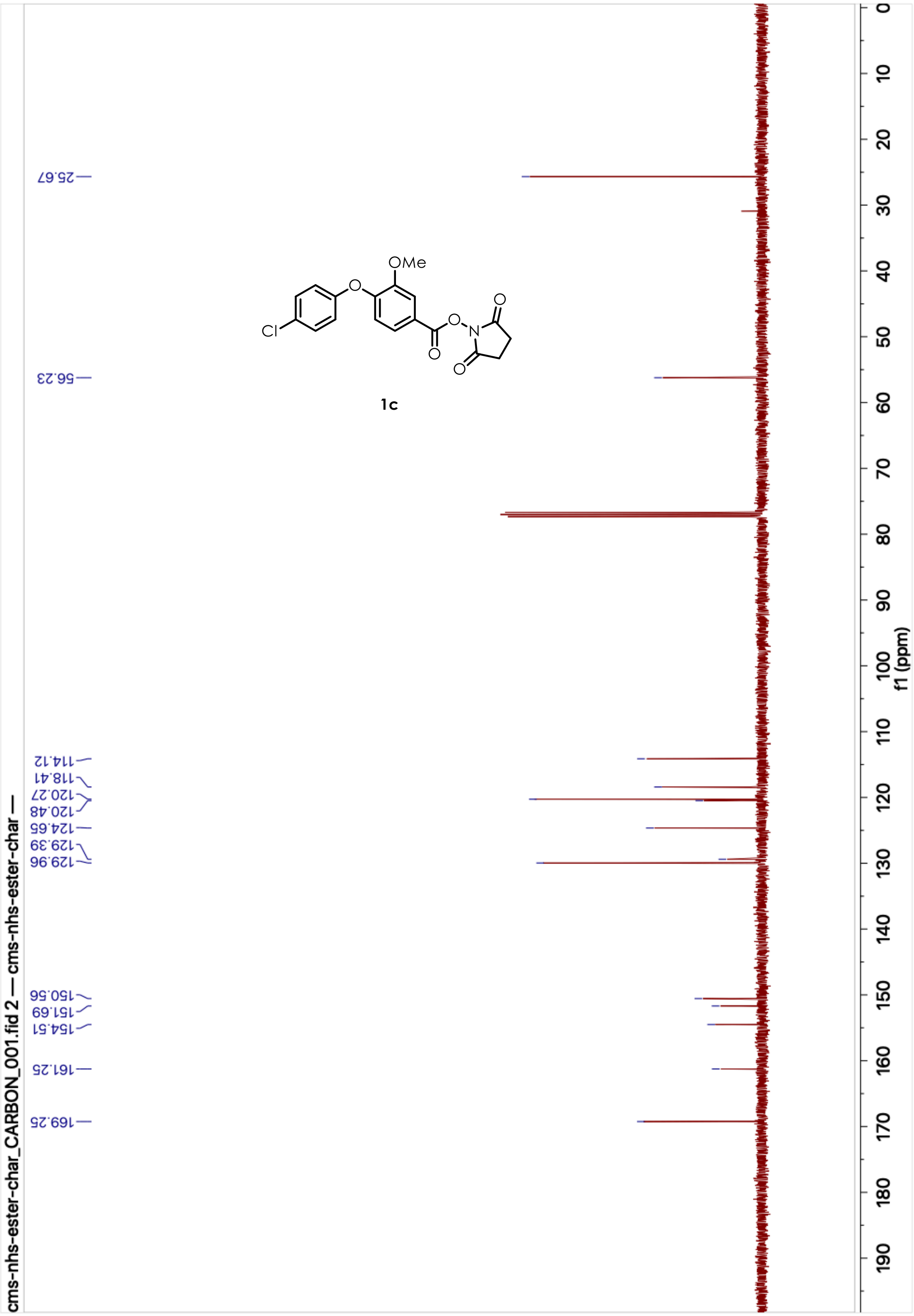
cms-nhs-ester-char_CARBON_001.fid 2 — cms-nhs-ester-char —
25.67
56.23
114.12
118.41
120.27
120.48
124.65
129.39
129.96
150.56
151.69
154.51
161.25
169.25
OMe
Cl
O
N
1c
f1 (ppm)

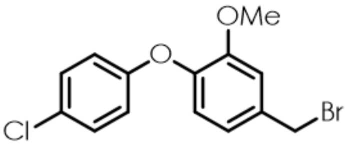

OMe
O
Cl
Br

1d

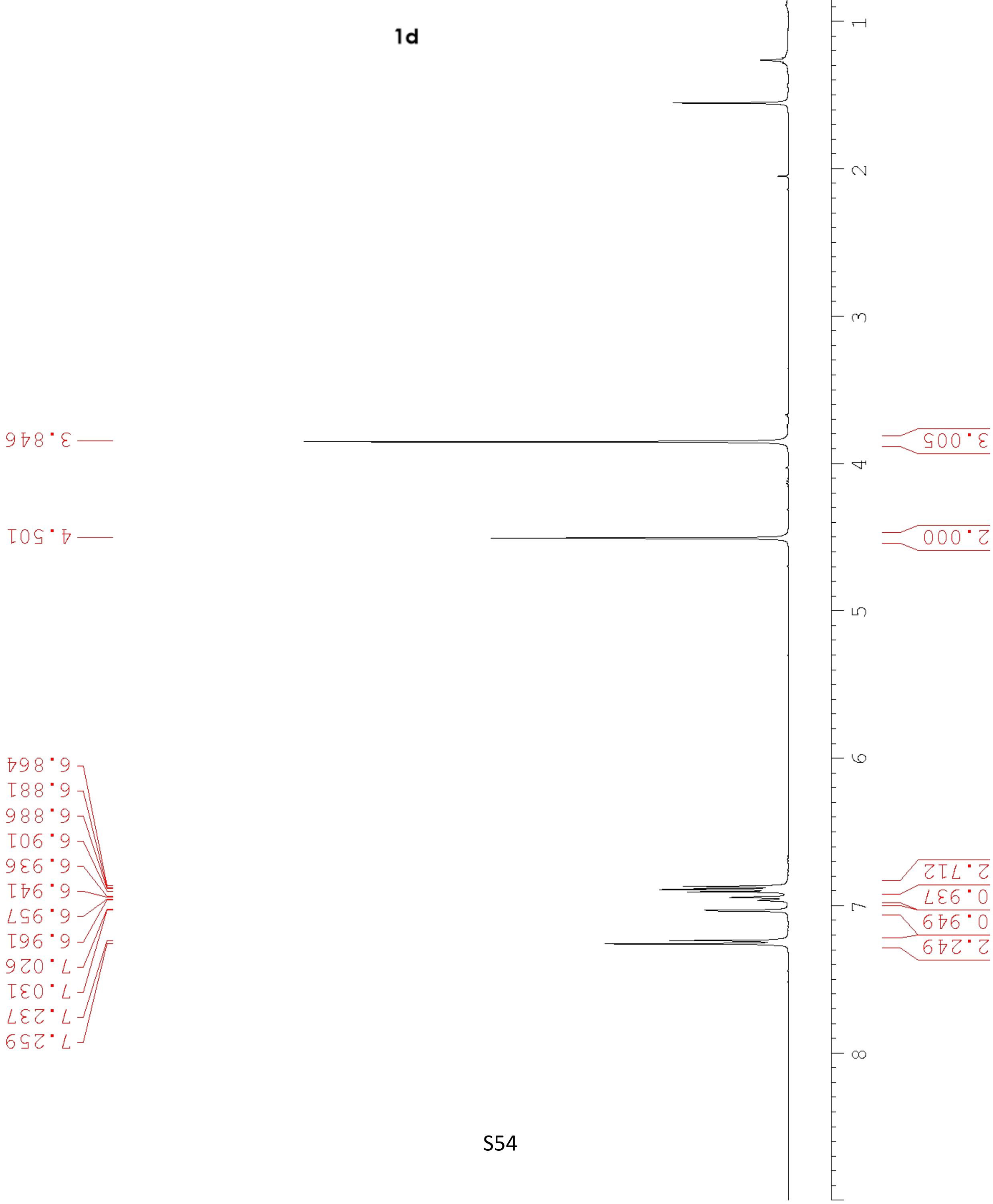

3.846
4.501
6.864
6.881
6.886
6.901
6.936
6.941
6.957
6.961
7.026
7.031
7.237
7.259
3.005
2.000
2.712
0.937
0.949
2.249
ppm
0
1
2
3
4
5
6
7
8

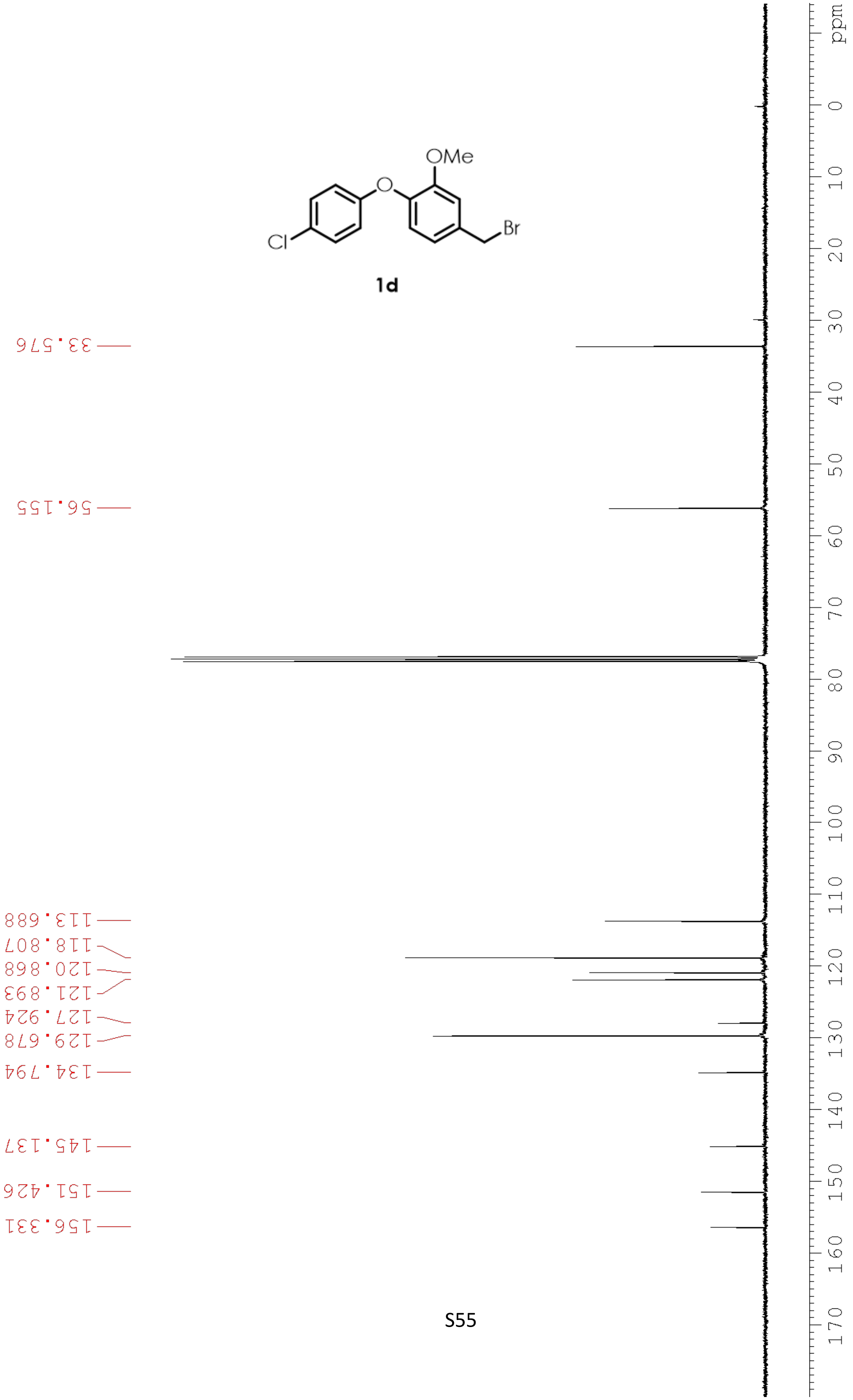
OMe
O
Cl
Br
1d
33.576
56.155
113.688
118.807
120.868
121.893
127.924
129.678
134.794
145.137
151.426
156.331
ppm

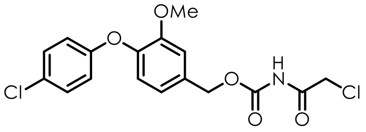
OMe
O
H
N
Cl
Cl
O
O
O

1e

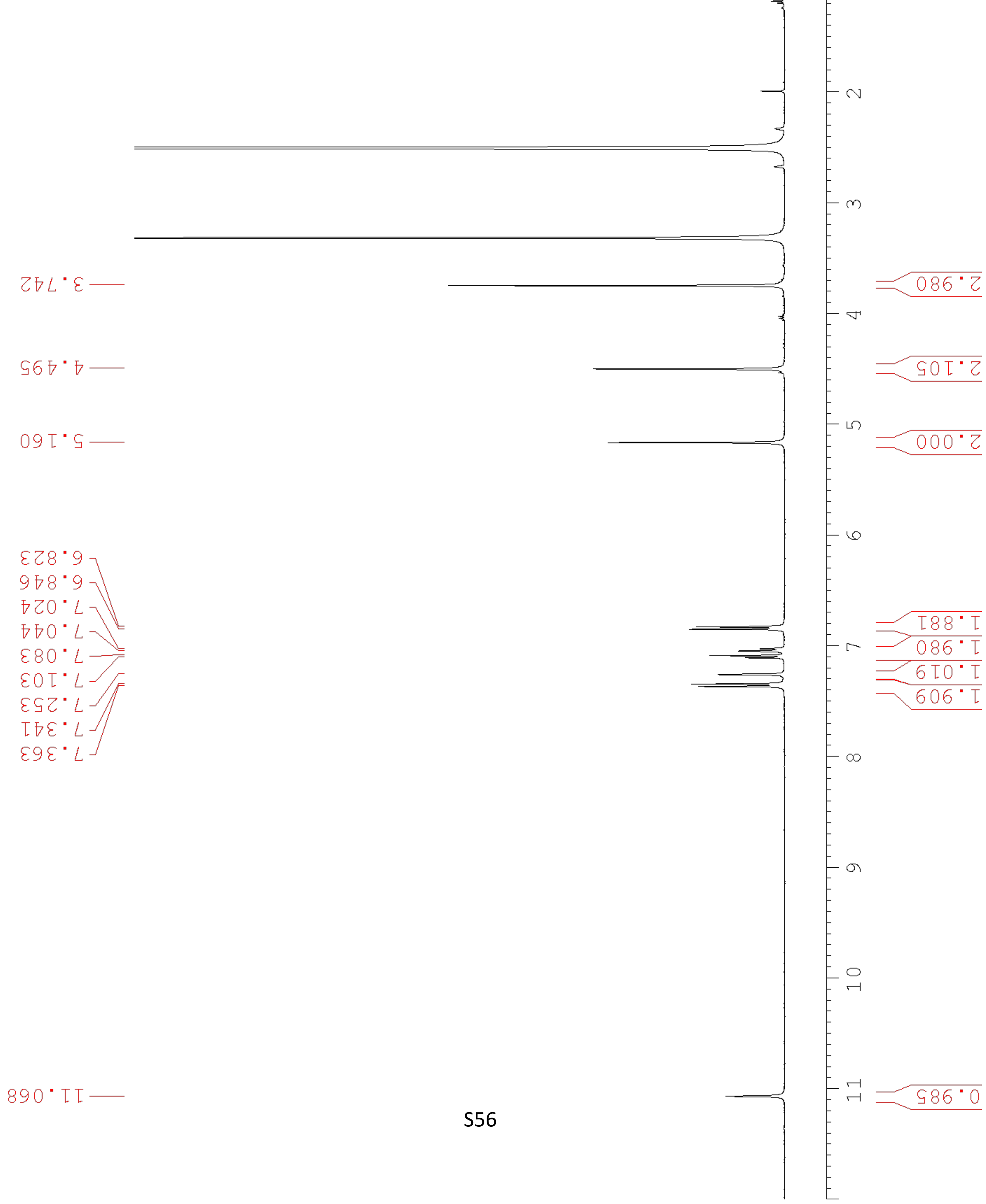
ppm
0
1
2
3
4
5
6
7
8
9
10
11
3.742
4.495
5.160
6.823
6.846
7.024
7.044
7.083
7.103
7.253
7.341
7.363
11.068
2.980
2.105
2.000
1.881
1.980
1.019
1.909
0.985

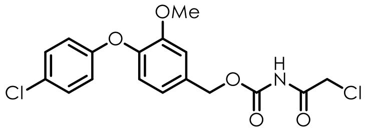
OMe
Cl
O
H
N
Cl
O
O
O

1e

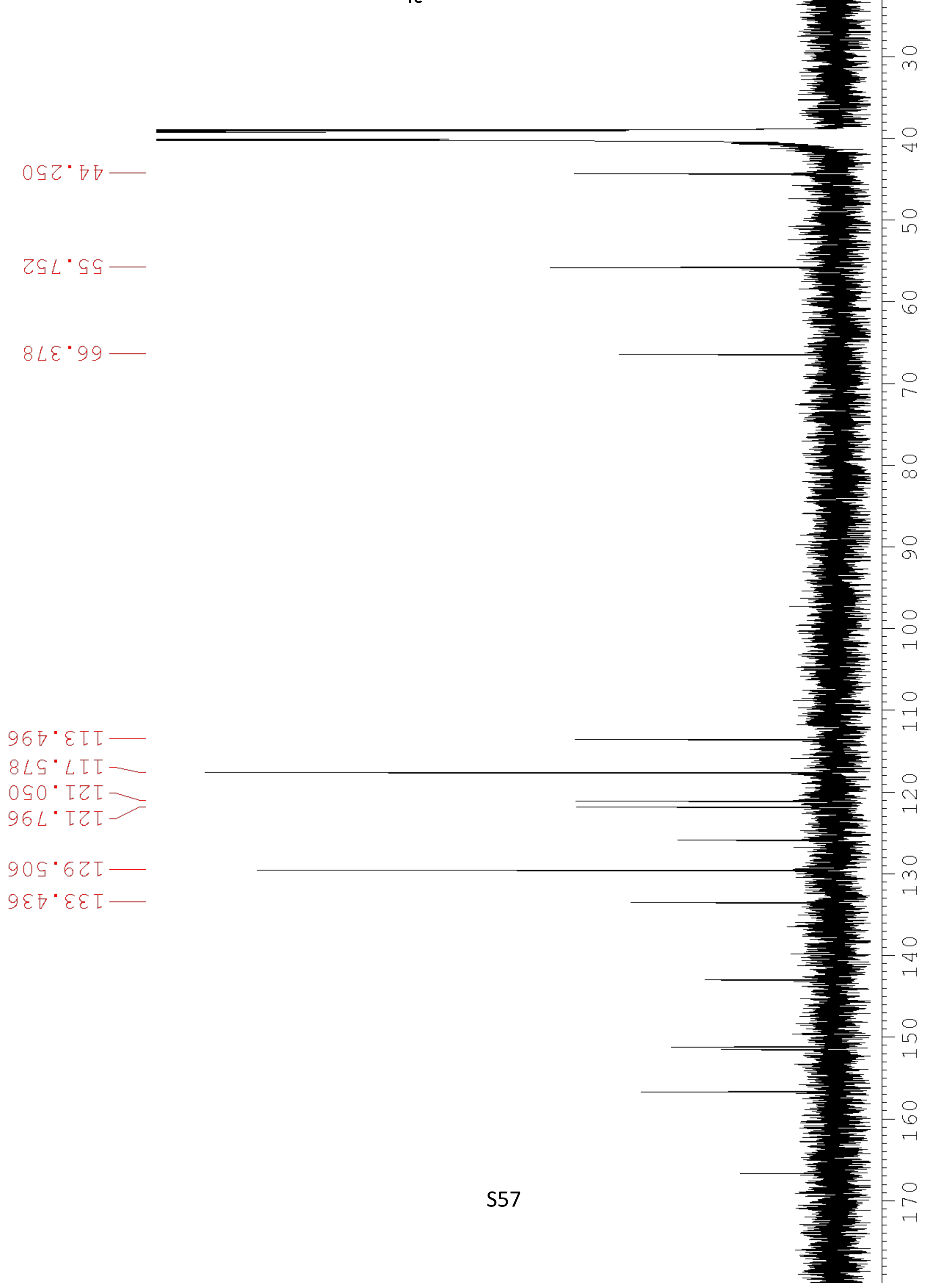
44.250
55.752
66.378
113.496
117.578
121.050
121.796
129.506
133.436
ppm
10
20
30
40
50
60
70
80
90
100
110
120
130
140
150
160
170

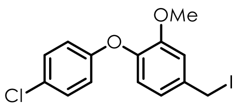
OMe
O
Cl
I

1f

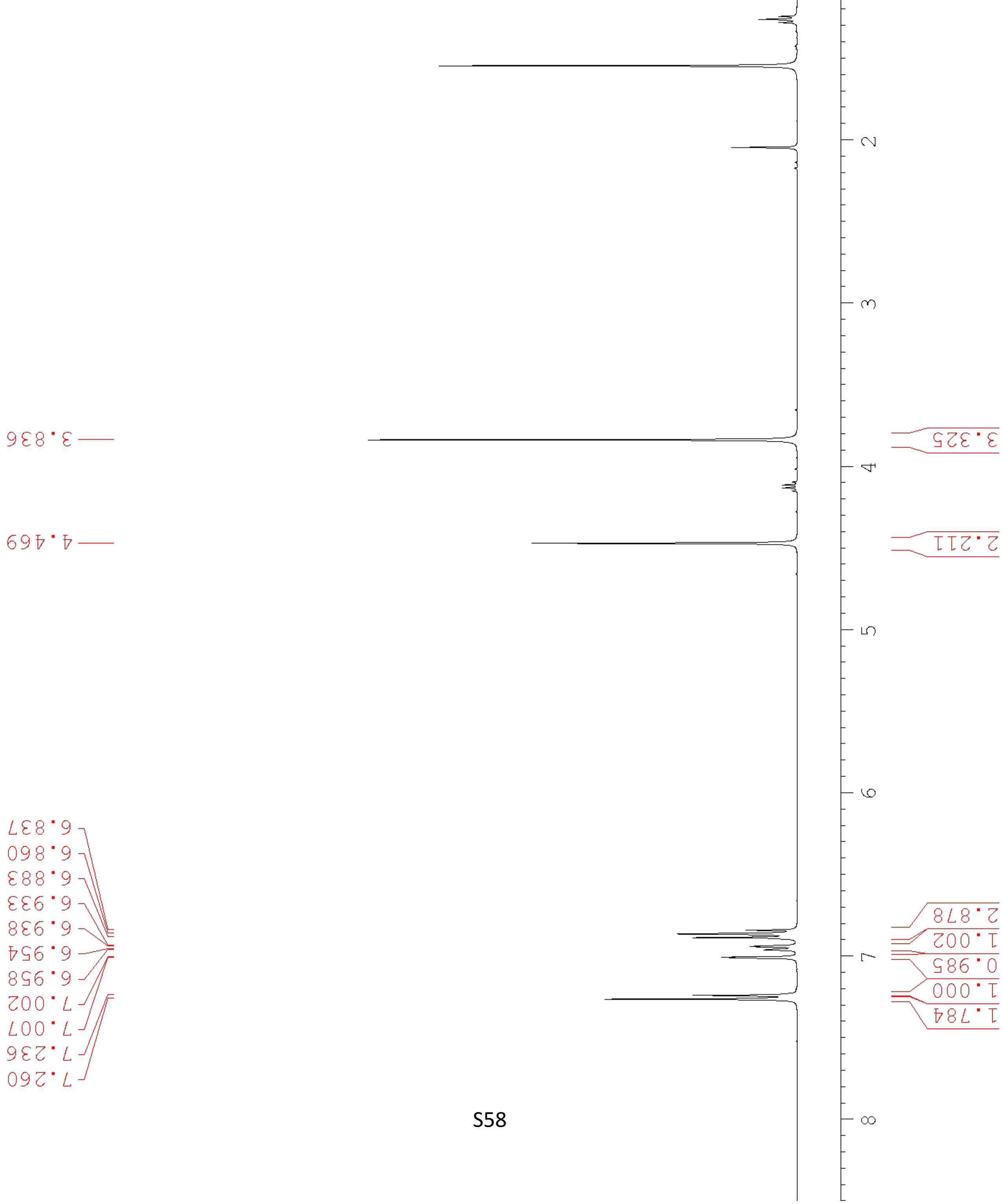
ppm
0
1
2
3
4
5
6
7
8
3.836
4.469
6.837
6.860
6.883
6.933
6.938
6.954
6.958
7.002
7.007
7.236
7.260
3.325
2.211
2.878
1.002
0.985
1.000
1.784

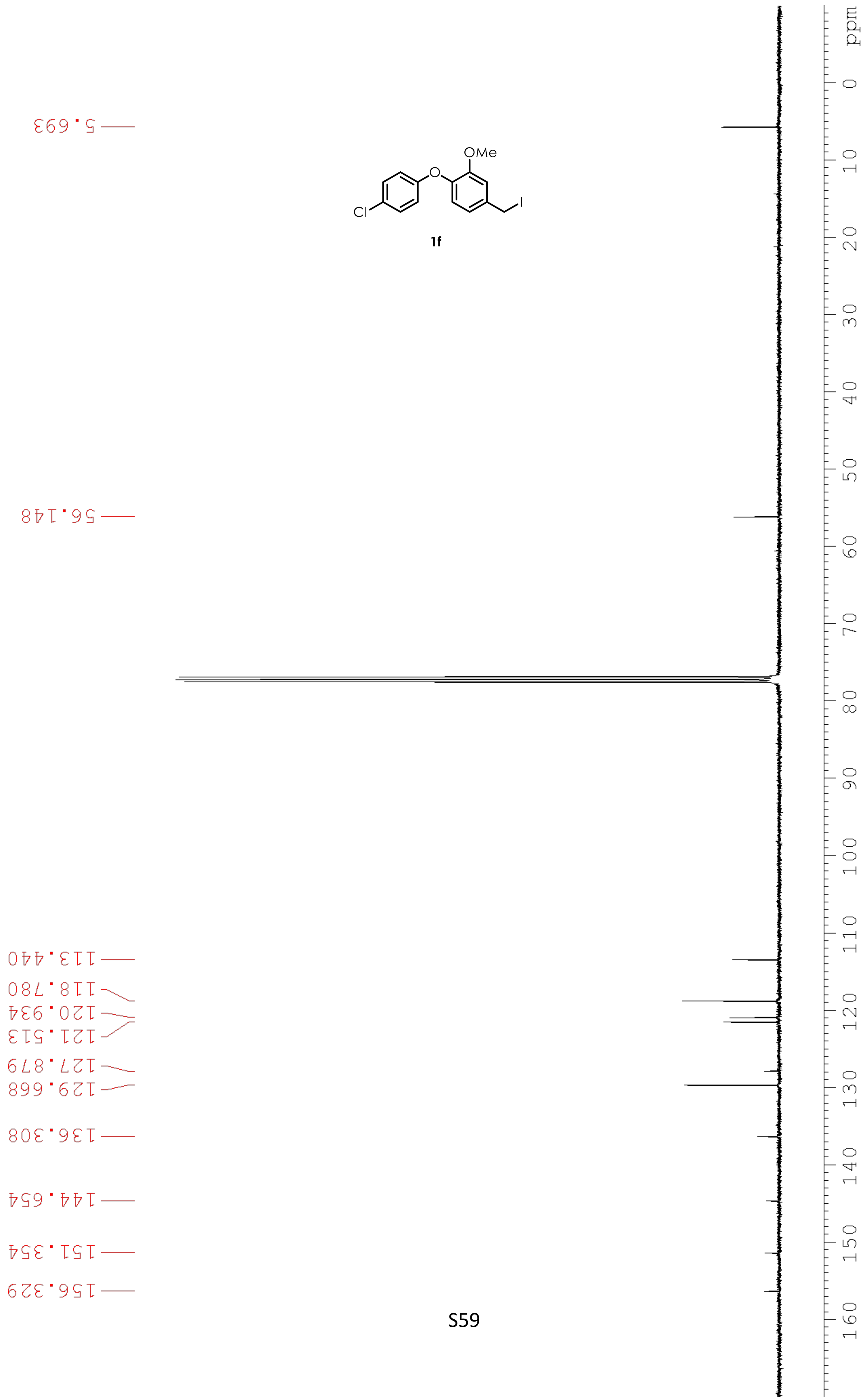

OMe
O
Cl
I
1f
5.693
56.148
113.440
118.780
120.934
121.513
127.879
129.668
136.308
144.654
151.354
156.329
ppm
0
10
20
30
40
50
60
70
80
90
100
110
120
130
140
150
160

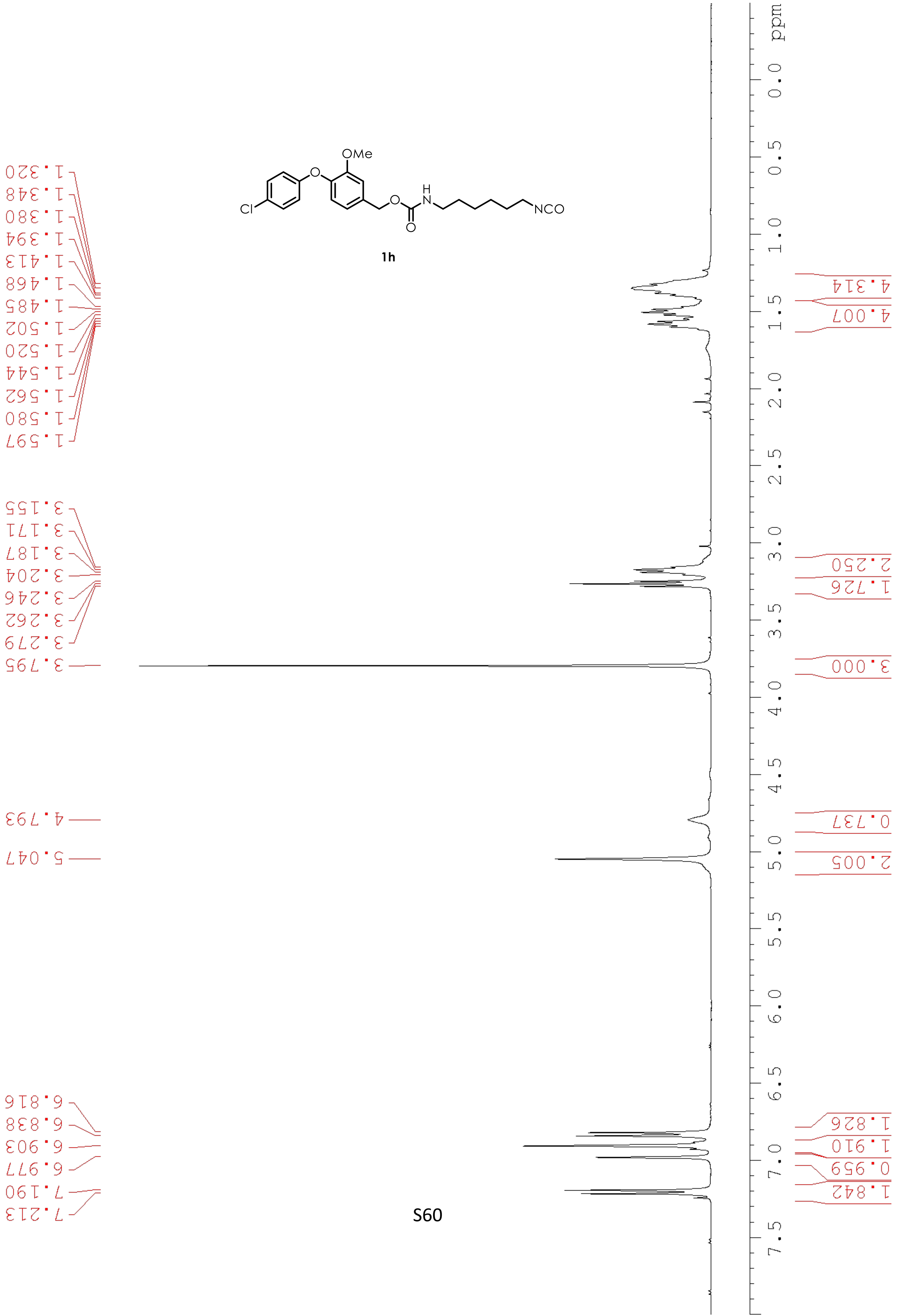
OMe
Cl
O
H
N
NCO
1h
ppm
1.320
1.348
1.380
1.394
1.413
1.468
1.485
1.502
1.520
1.544
1.562
1.580
1.597
3.155
3.171
3.187
3.204
3.246
3.262
3.279
3.795
4.793
5.047
6.816
6.838
6.903
6.977
7.190
7.213
4.314
4.007
2.250
1.726
3.000
0.737
2.005
1.826
1.910
0.959
1.842

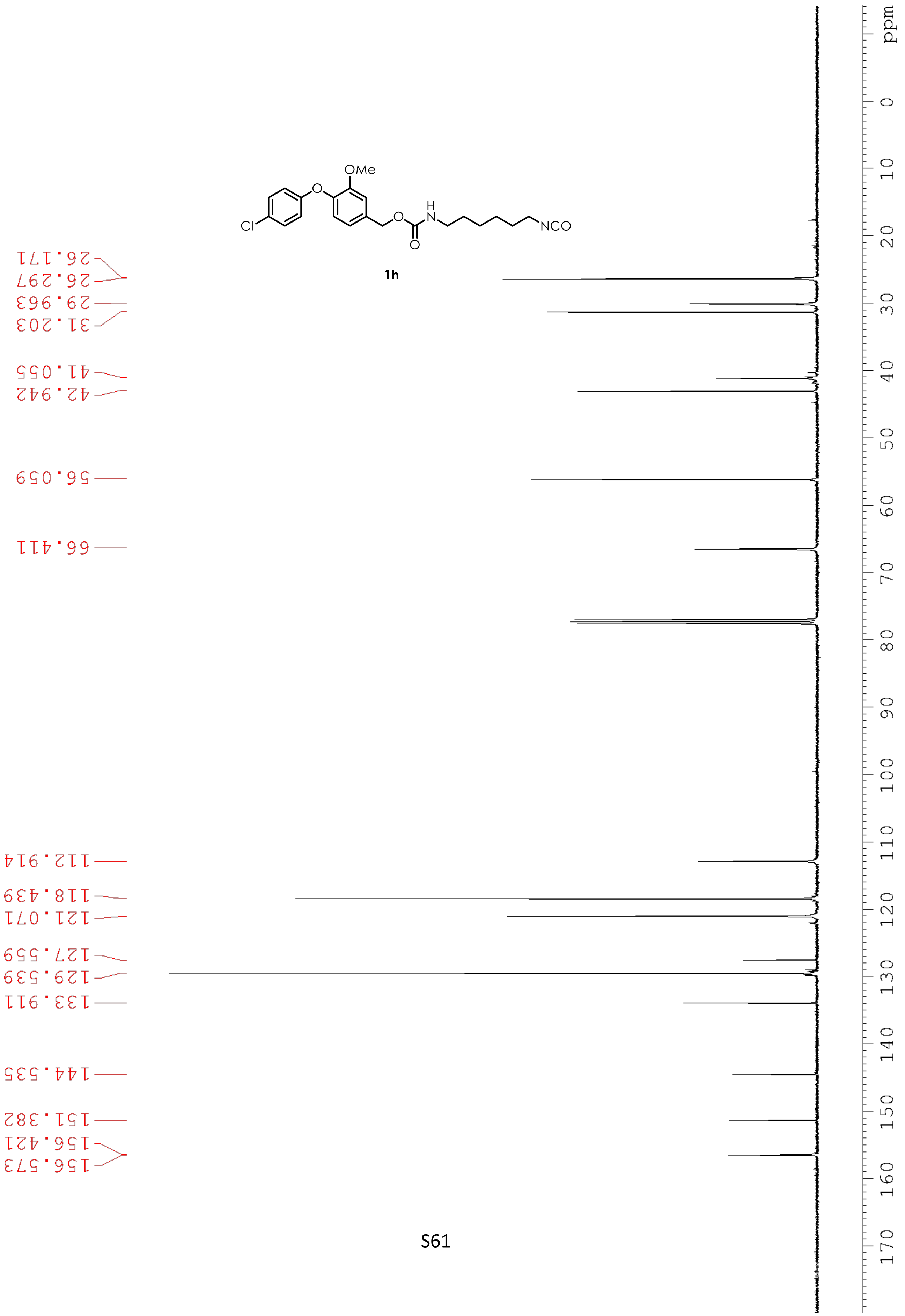
OMe
O
Cl
O
H
N
O
NCO
1h
26.171
26.297
29.963
31.203
41.055
42.942
56.059
66.411
112.914
118.439
121.071
127.559
129.539
133.911
144.535
151.382
156.421
156.573
ppm

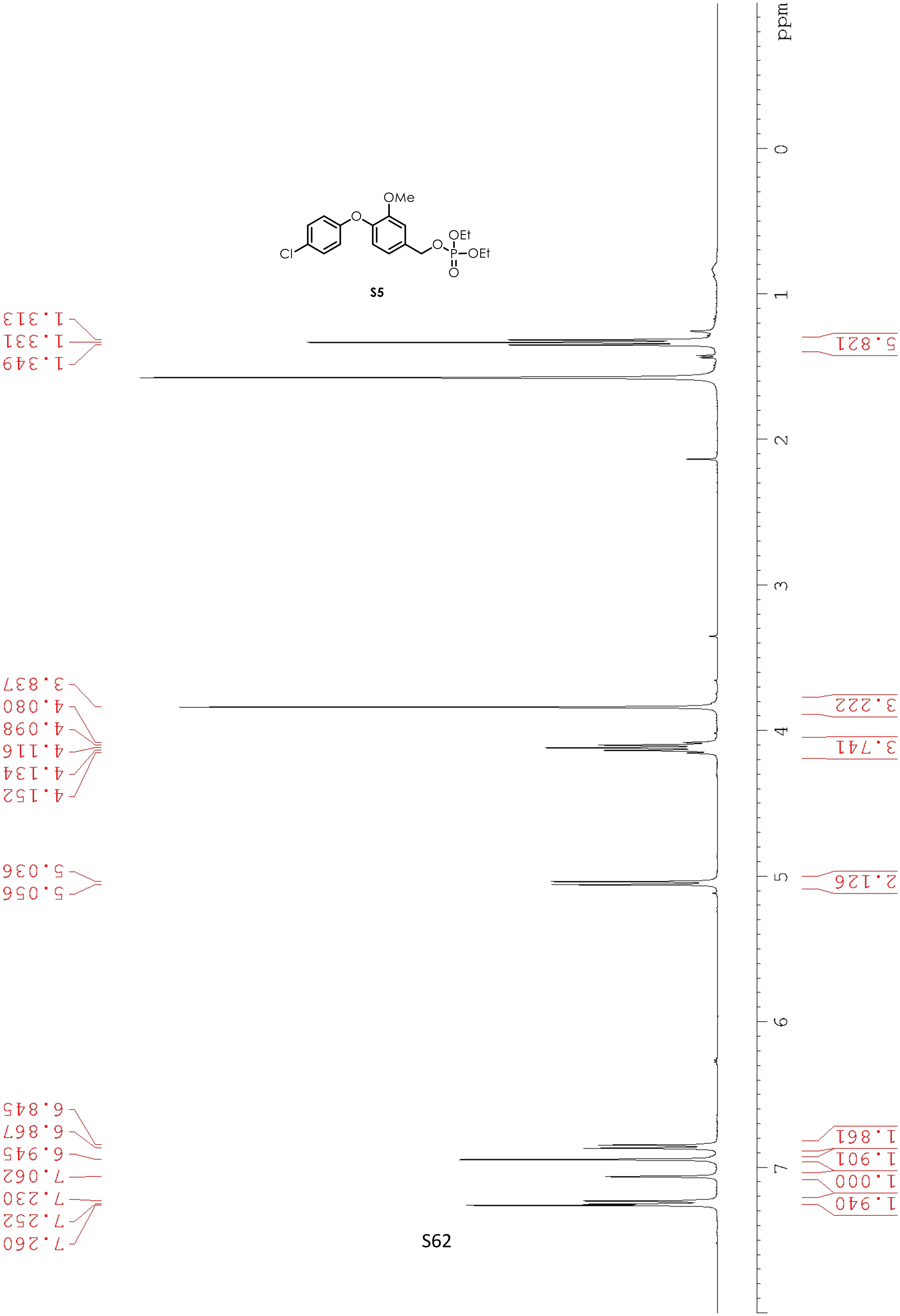
OMe
OEt
OEt
Cl
O
P
S5
1.313
1.331
1.349
3.837
4.080
4.098
4.116
4.134
4.152
5.036
5.056
6.845
6.867
6.945
7.062
7.230
7.252
7.260
5.821
3.222
3.741
2.126
1.861
1.901
1.000
1.940
ppm
0
1
2
3
4
5
6
7

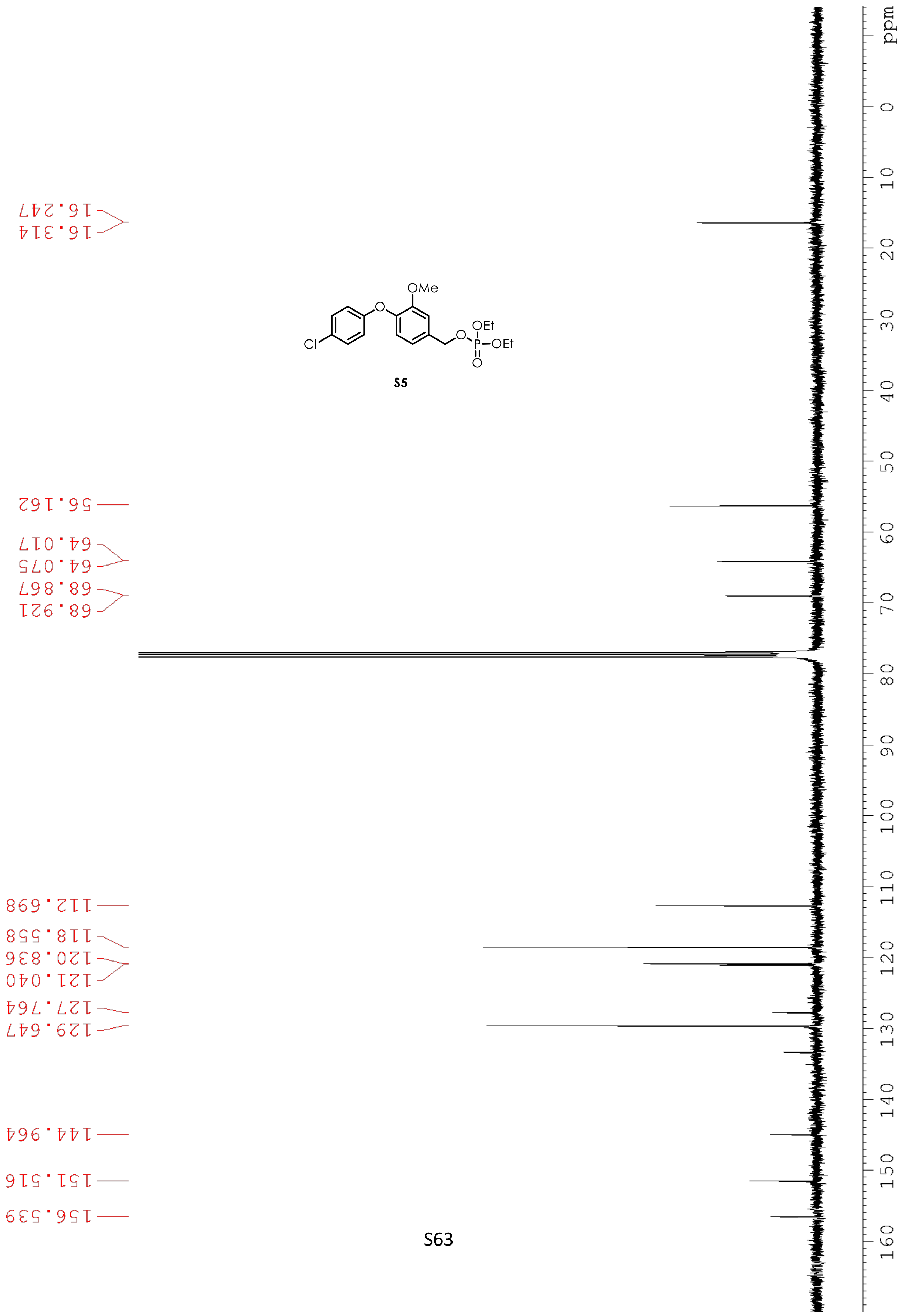
16.247
16.314
56.162
64.017
64.075
68.867
68.921
112.698
118.558
120.836
121.040
127.764
129.647
144.964
151.516
156.539
ppm
OMe
O
Cl
O
OEt
P
OEt
O
S5

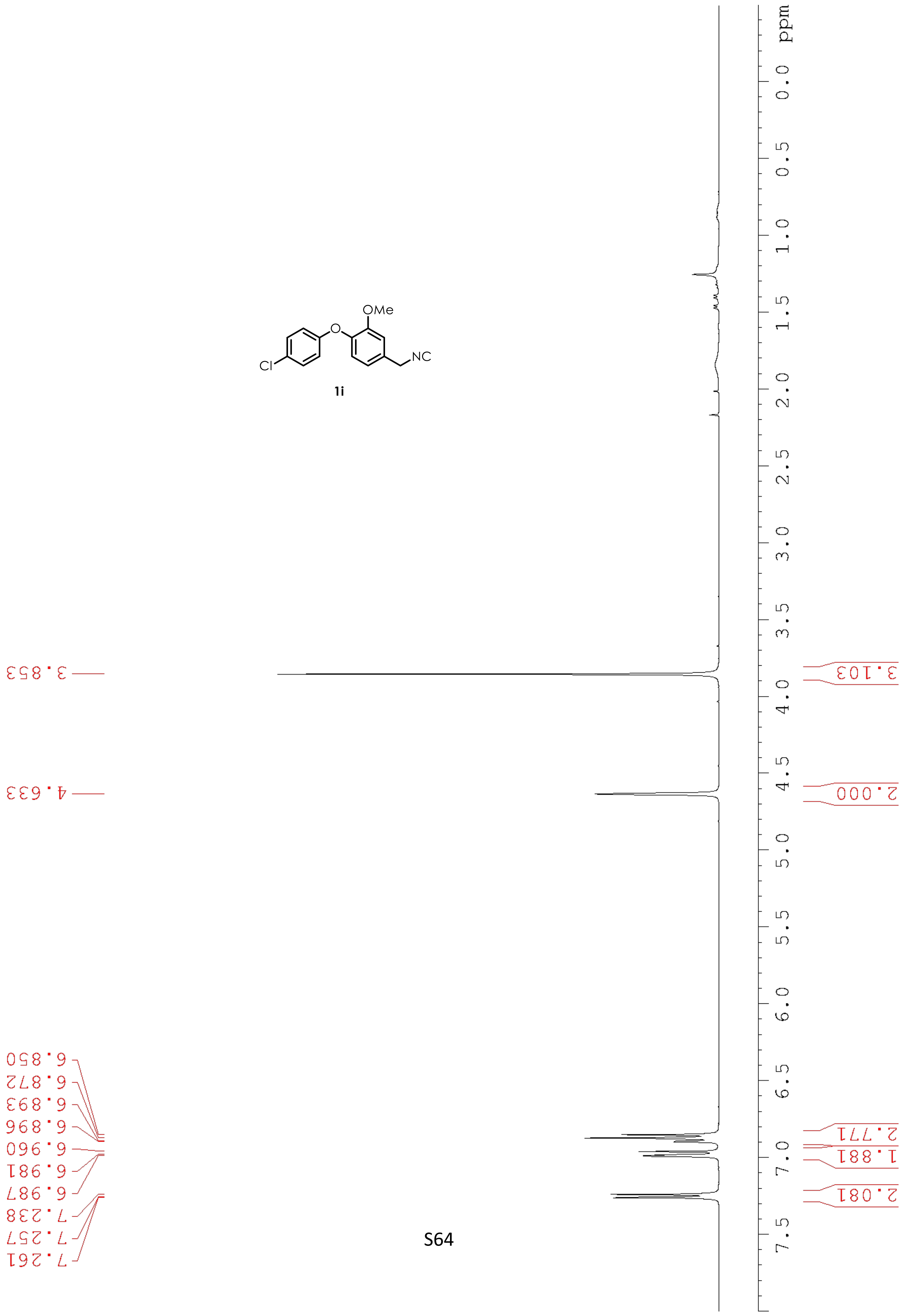
OMe
O
Cl
NC
1i
3.853
4.633
6.850
6.872
6.893
6.896
6.960
6.981
6.987
7.238
7.257
7.261
3.103
2.000
2.771
1.881
2.081
ppm

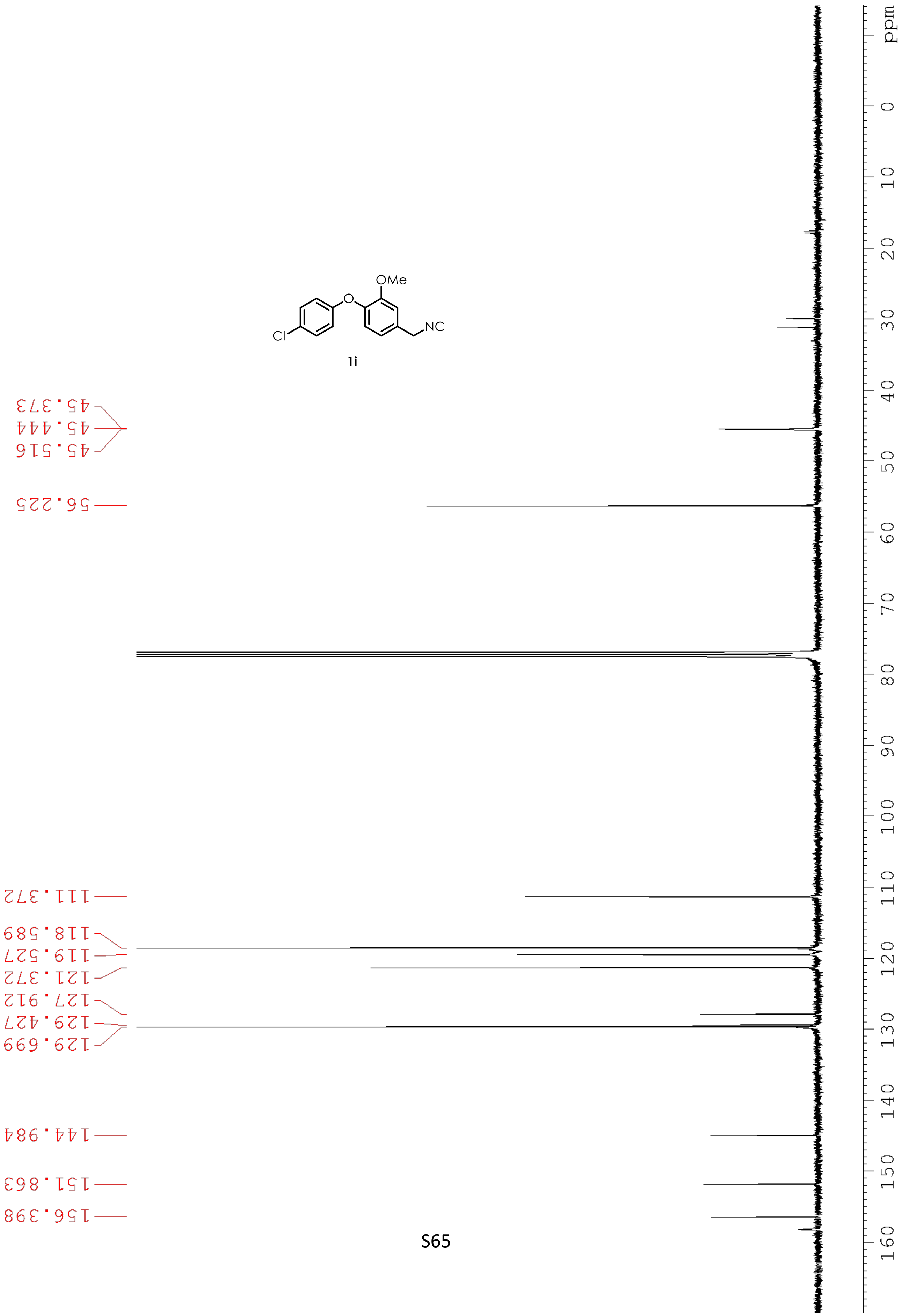
OMe
Cl
O
NC
1i
45.373
45.444
45.516
56.225
111.372
118.589
119.527
121.372
127.912
129.427
129.699
144.984
151.863
156.398
ppm

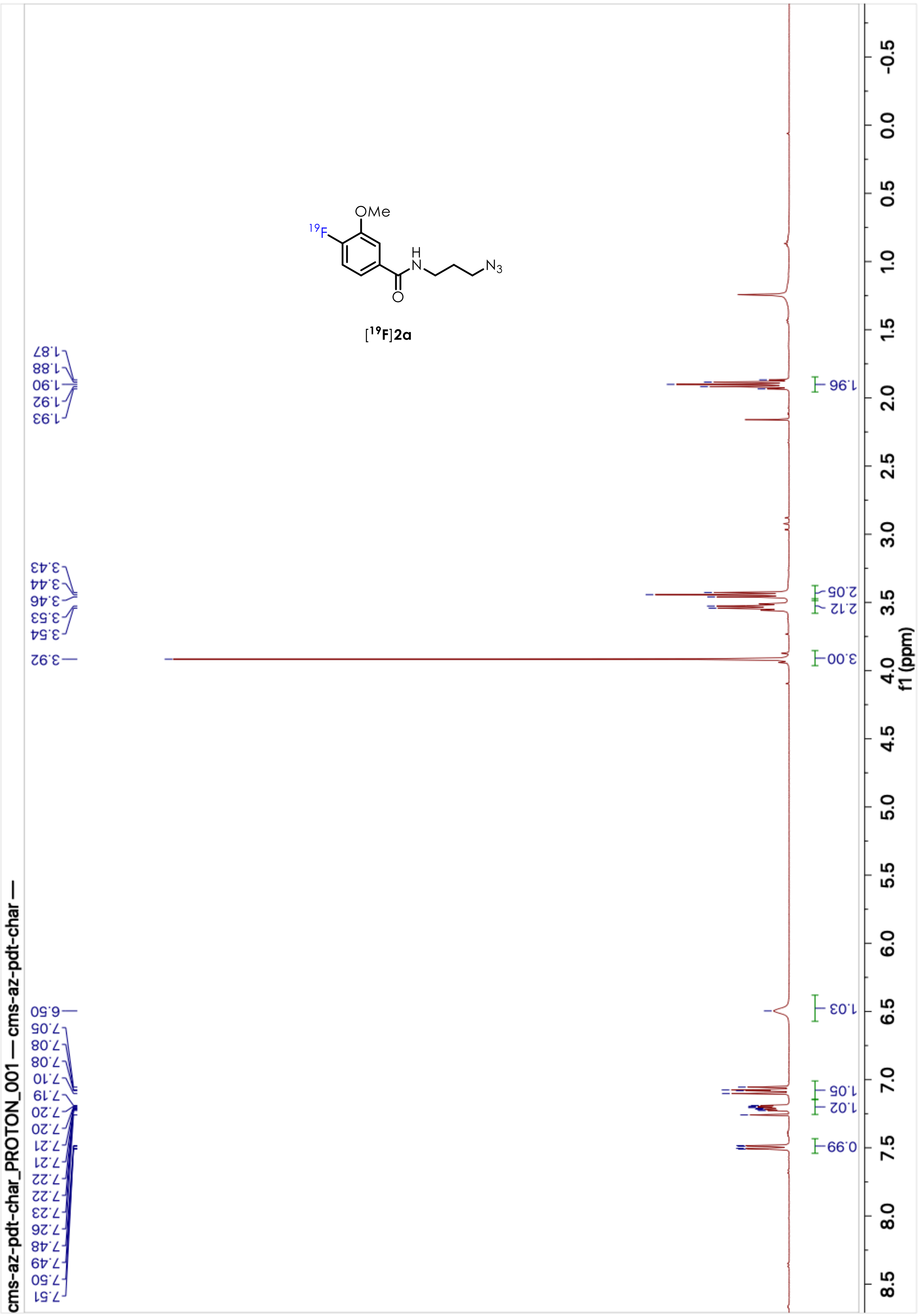
cms-az-pdt-char_PROTON_001 — cms-az-pdt-char —
7.51
7.50
7.49
7.48
7.26
7.23
7.22
7.22
7.21
7.21
7.20
7.20
7.19
7.10
7.08
7.08
7.05
6.50
3.92
3.54
3.53
3.46
3.44
3.43
1.93
1.92
1.90
1.88
1.87
OMe
19F
H
N
N3
O
[19F]2a
0.99
1.02
1.05
1.03
3.00
2.12
2.05
1.96
f1 (ppm)

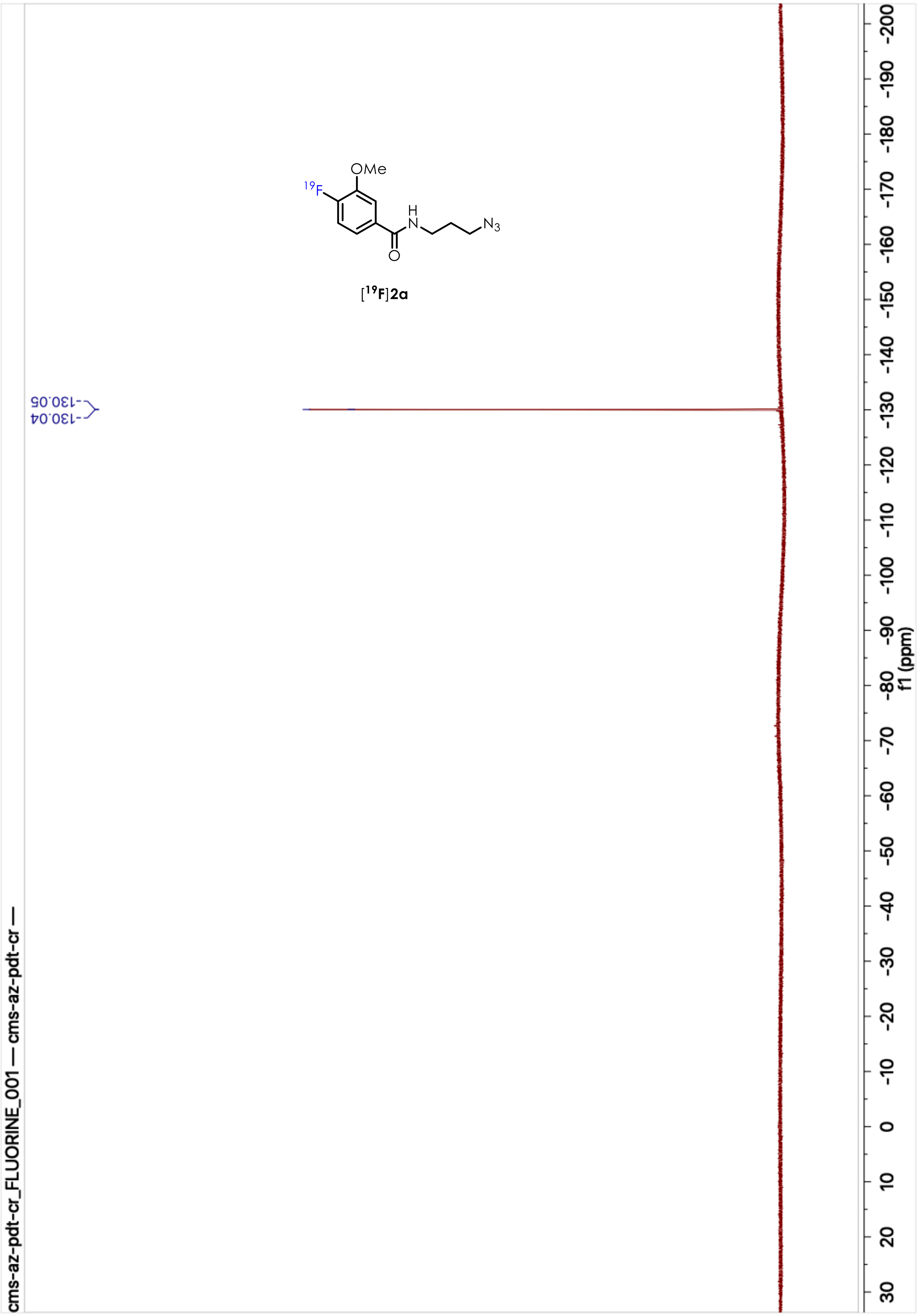
cms-az-pdt-cr_FLUORINE_001 — cms-az-pdt-cr —
-130.05
-130.04
OMe
19F
H
N
N3
O
[19F]2a
f1 (ppm)

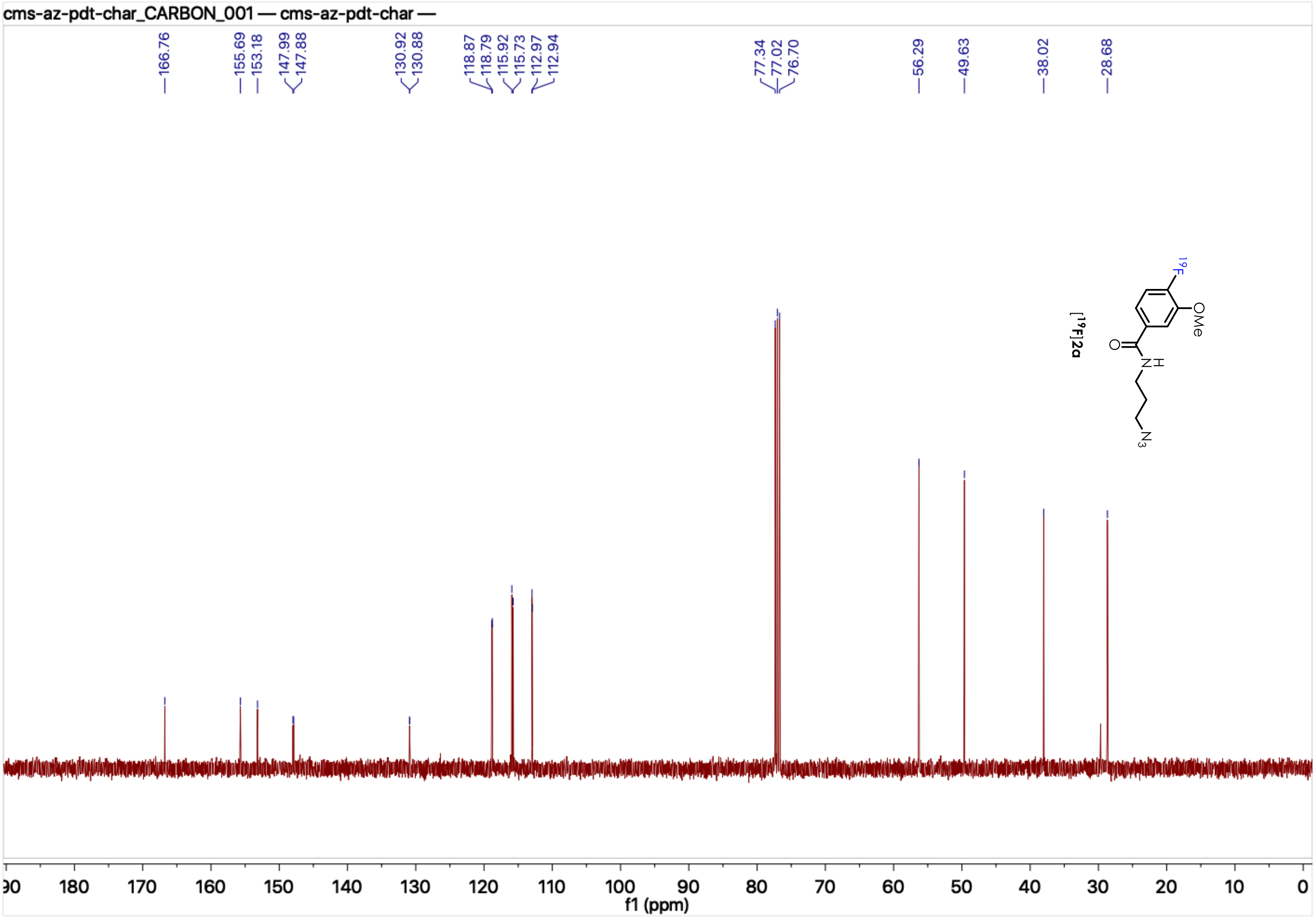
cms-az-pdt-char_CARBON_001 — cms-az-pdt-char —
166.76
155.69
153.18
147.99
147.88
130.92
130.88
118.87
118.79
115.92
115.73
112.97
112.94
77.34
77.02
76.70
56.29
49.63
38.02
28.68
OMe
19F
H
N
N3
O
[19F]2a
f1 (ppm)

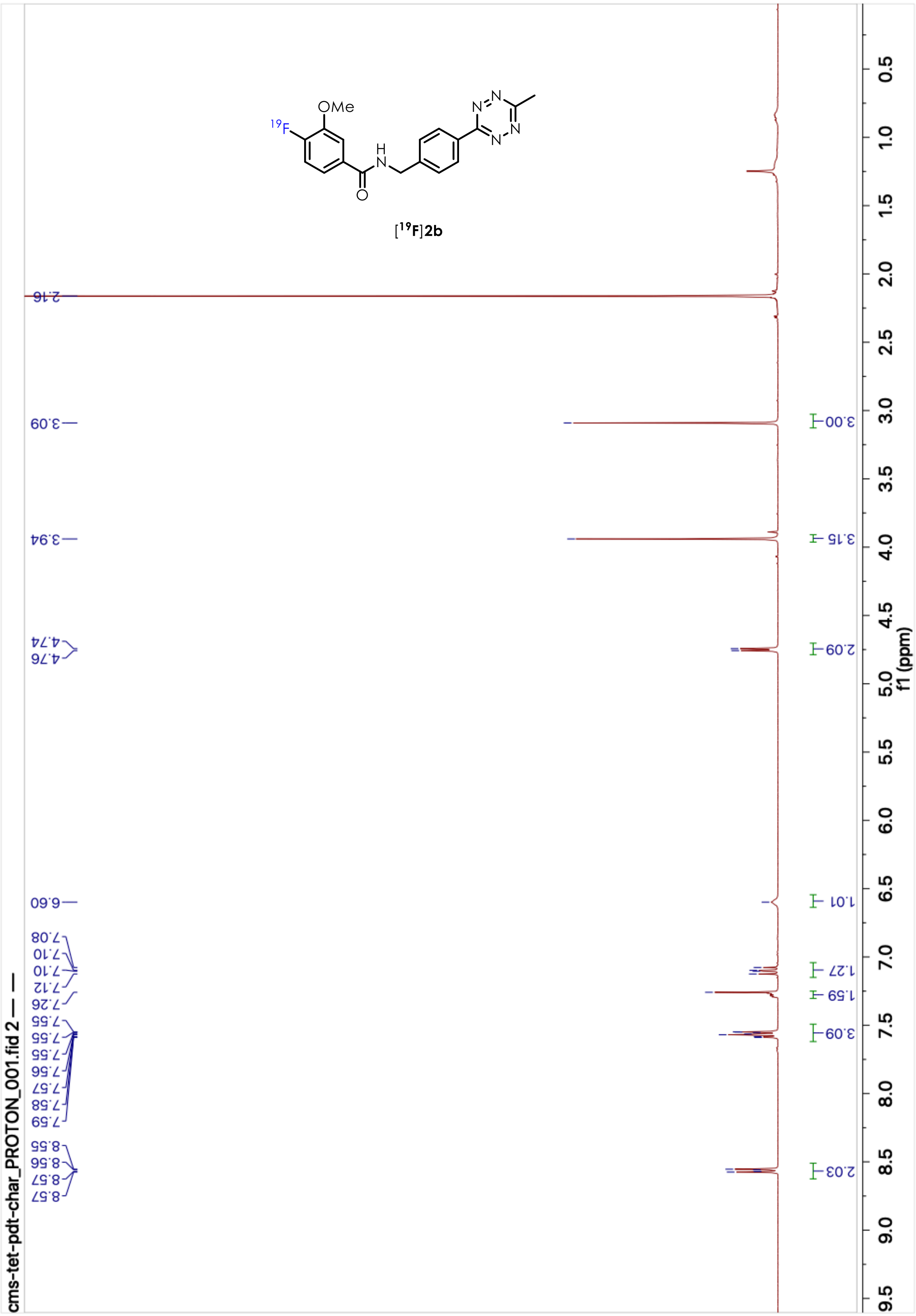
cms-tet-pdt-char_PROTON_001.fid 2
[19F]2b
OMe
19F
H
N
O
N
N
N
N
2.16
3.09
3.94
4.74
4.76
6.60
7.08
7.10
7.10
7.12
7.26
7.55
7.55
7.55
7.56
7.57
7.58
7.59
8.55
8.56
8.57
8.57
3.00
3.15
2.09
1.01
1.27
1.59
3.09
2.03
f1 (ppm)
0.5
1.0
1.5
2.0
2.5
3.0
3.5
4.0
4.5
5.0
5.5
6.0
6.5
7.0
7.5
8.0
8.5
9.0
9.5

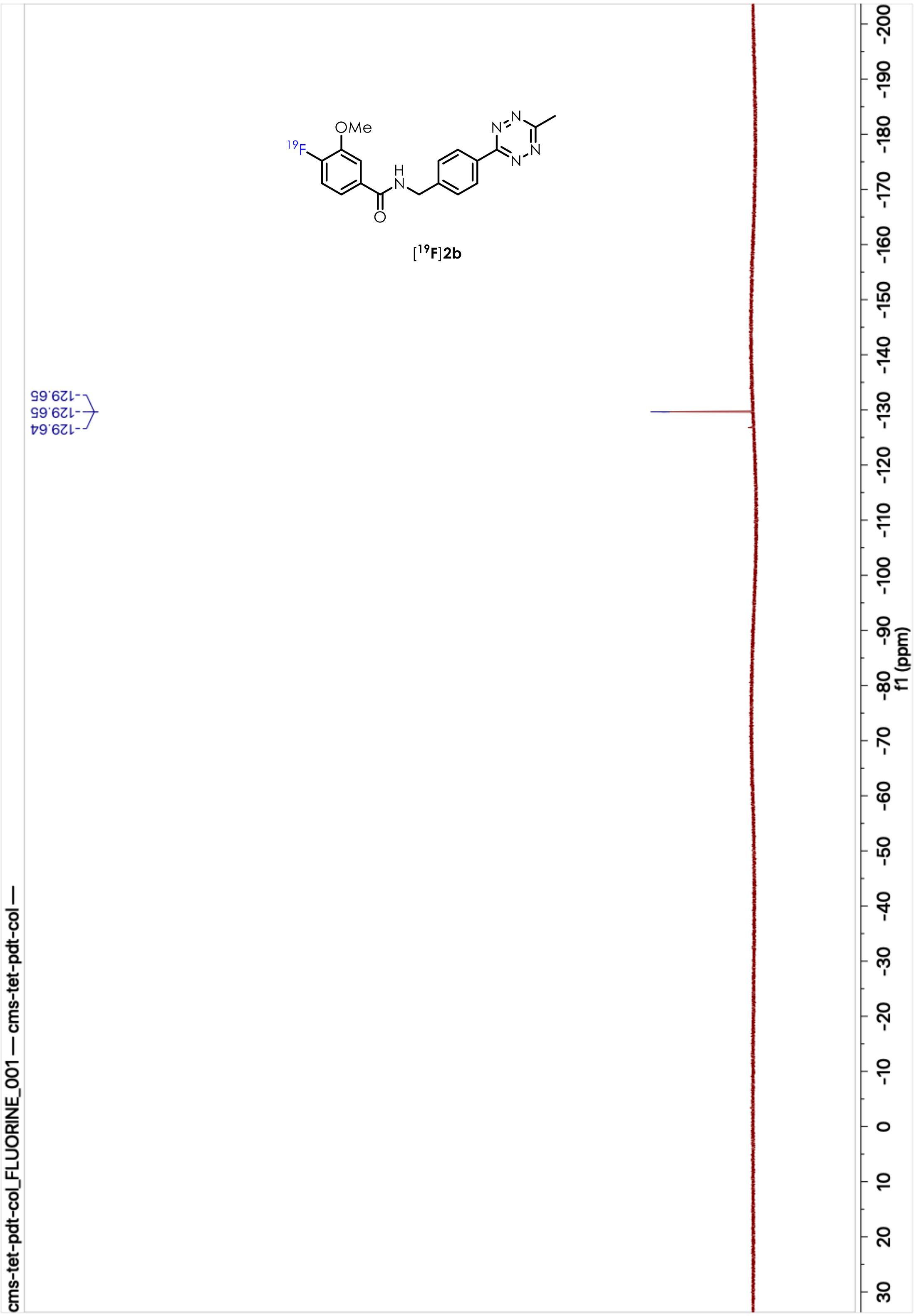
cms-tet-pdt-col_FLUORINE_001 — cms-tet-pdt-col —
-129.65
-129.65
-129.64
OMe
19F
H
N
O
[19F]2b
f1 (ppm)
30 20 10 0 -10 -20 -30 -40 -50 -60 -70 -80 -90 -100 -110 -120 -130 -140 -150 -160 -170 -180 -190 -200

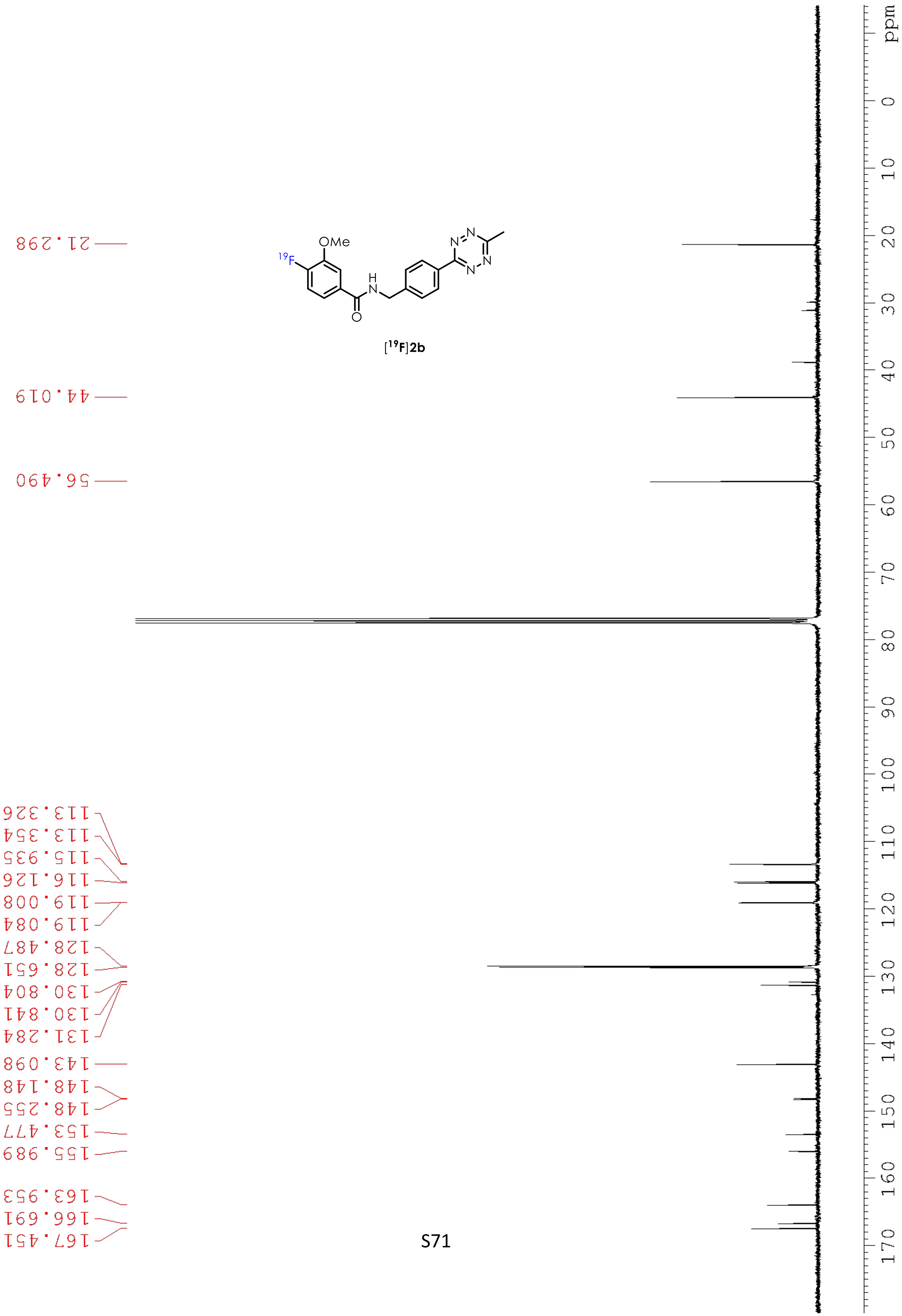
OMe
19F
H
N
O
N
21.298
44.019
56.490
113.326
113.354
115.935
116.126
119.008
119.084
128.487
128.651
130.804
130.841
131.284
143.098
148.148
148.255
153.477
155.989
163.953
166.691
167.451
ppm
0
10
20
30
40
50
60
70
80
90
100
110
120
130
140
150
160
170

[19F]2b

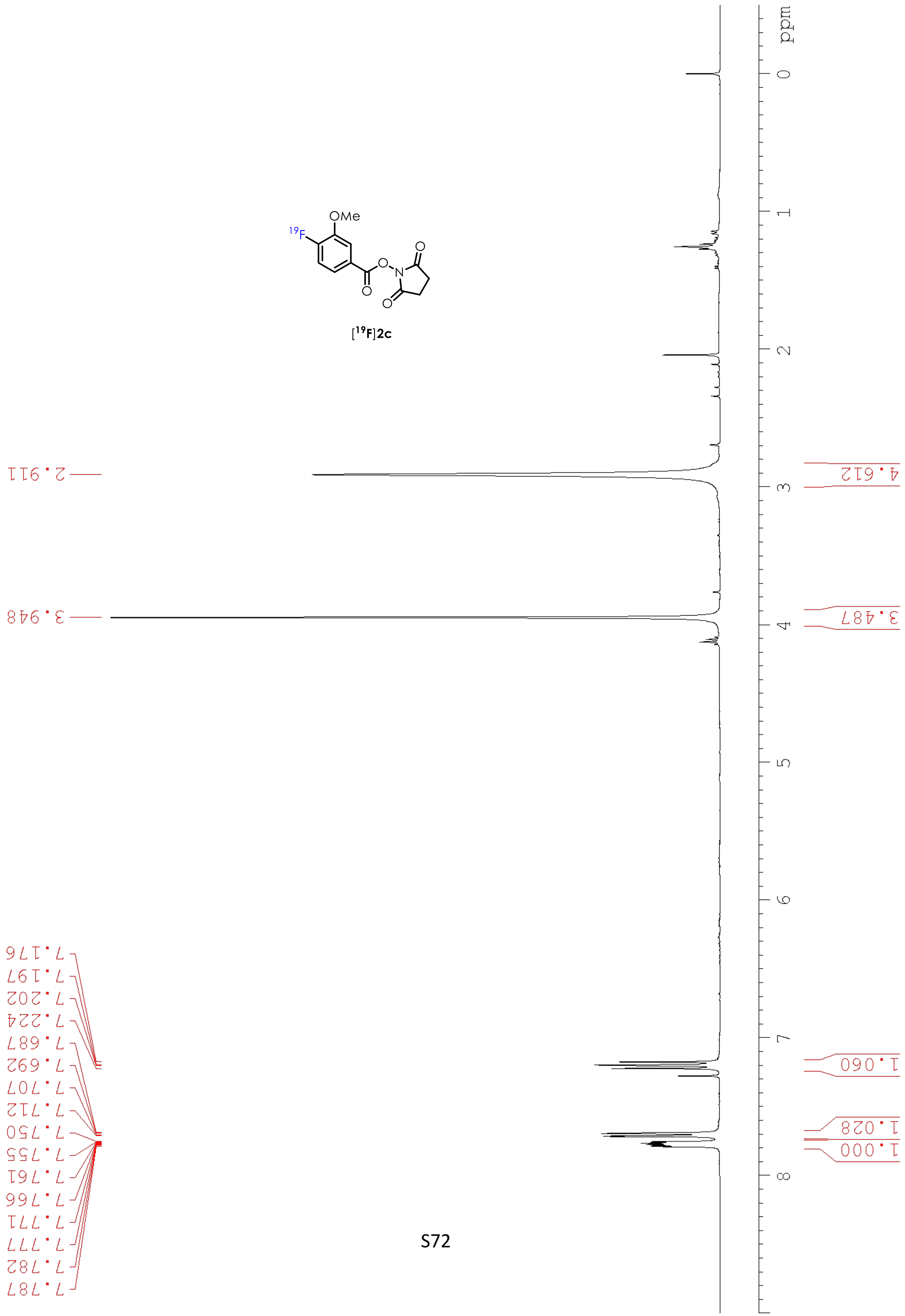
OMe
19F
O
N
O
O
O
[19F]2c
2.911
3.948
7.176
7.197
7.202
7.224
7.687
7.692
7.707
7.712
7.750
7.755
7.761
7.766
7.771
7.777
7.782
7.787
4.612
3.487
1.060
1.028
1.000
ppm
0
1
2
3
4
5
6
7
8

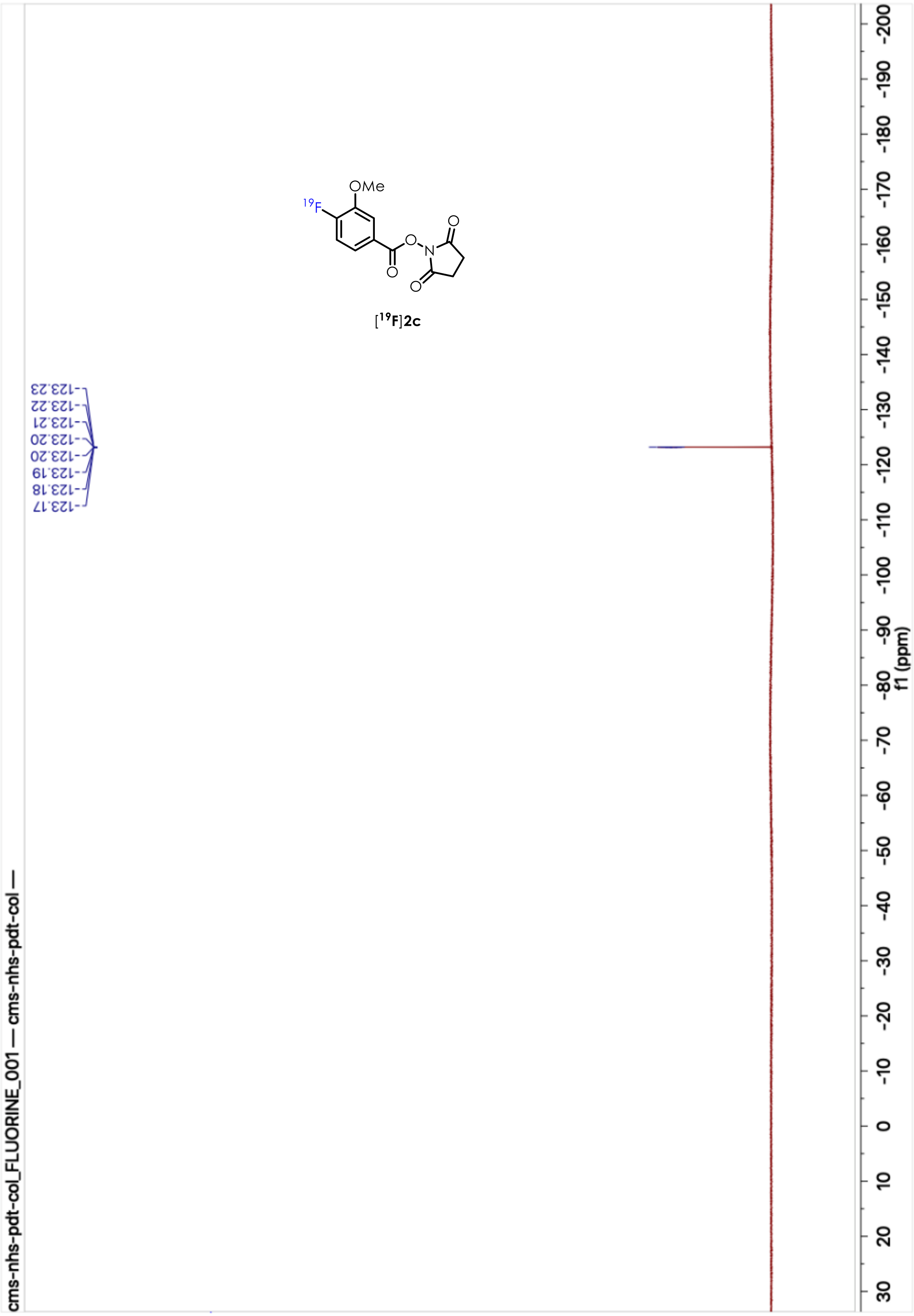
cms-nhs-pdt-col_FLUORINE_001 — cms-nhs-pdt-col —
OMe
19F
O
N
O
O
O
[19F]2c
-123.23
-123.22
-123.21
-123.20
-123.20
-123.19
-123.18
-123.17
30 20 10 0 -10 -20 -30 -40 -50 -60 -70 -80 -90 -100 -110 -120 -130 -140 -150 -160 -170 -180 -190 -200
f1 (ppm)

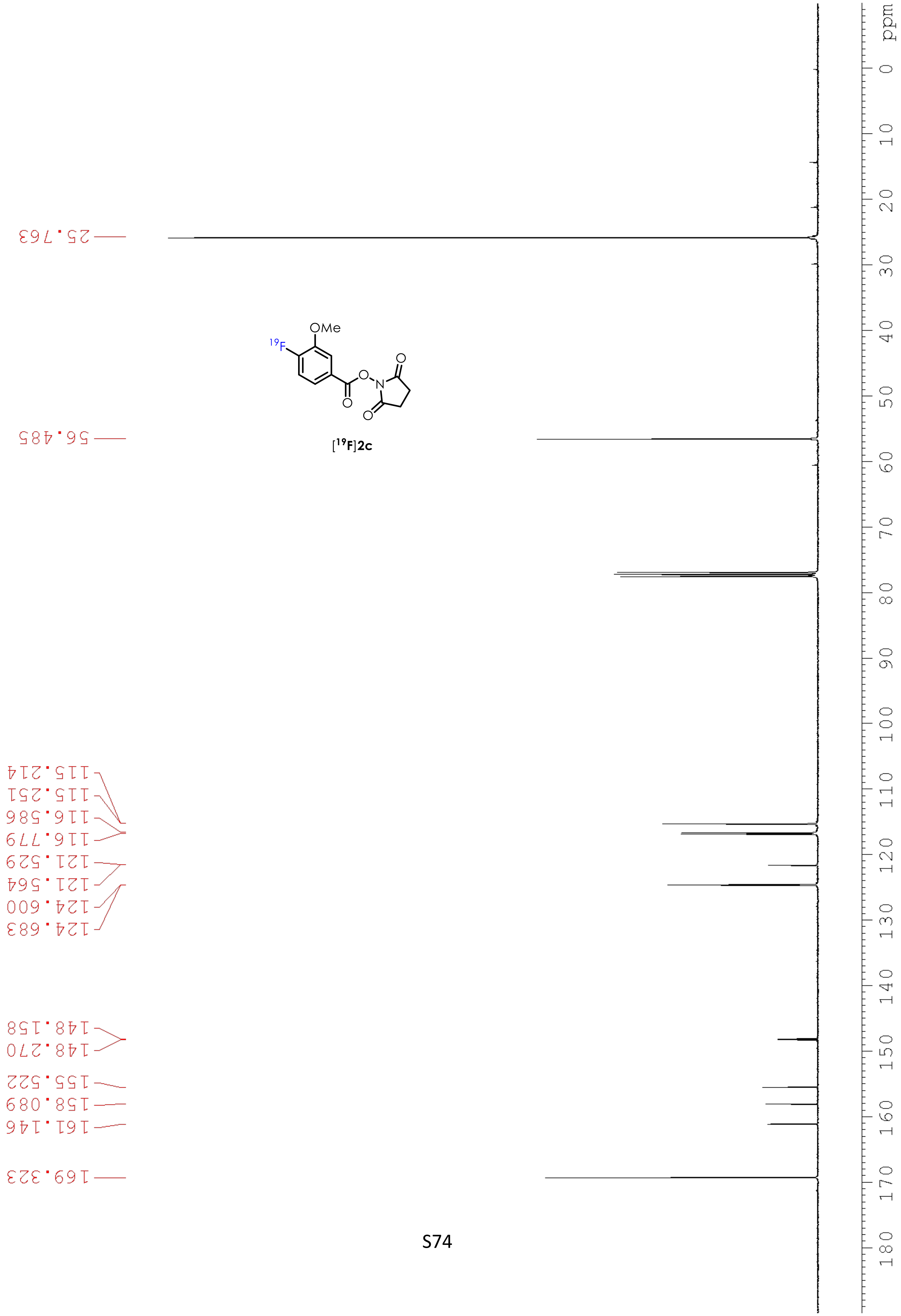
25.763
56.485
115.214
115.251
116.586
116.779
121.529
121.564
124.600
124.683
148.158
148.270
155.522
158.089
161.146
169.323
OMe
19F
O
N
[19F]2c
ppm
0
10
20
30
40
50
60
70
80
90
100
110
120
130
140
150
160
170
180

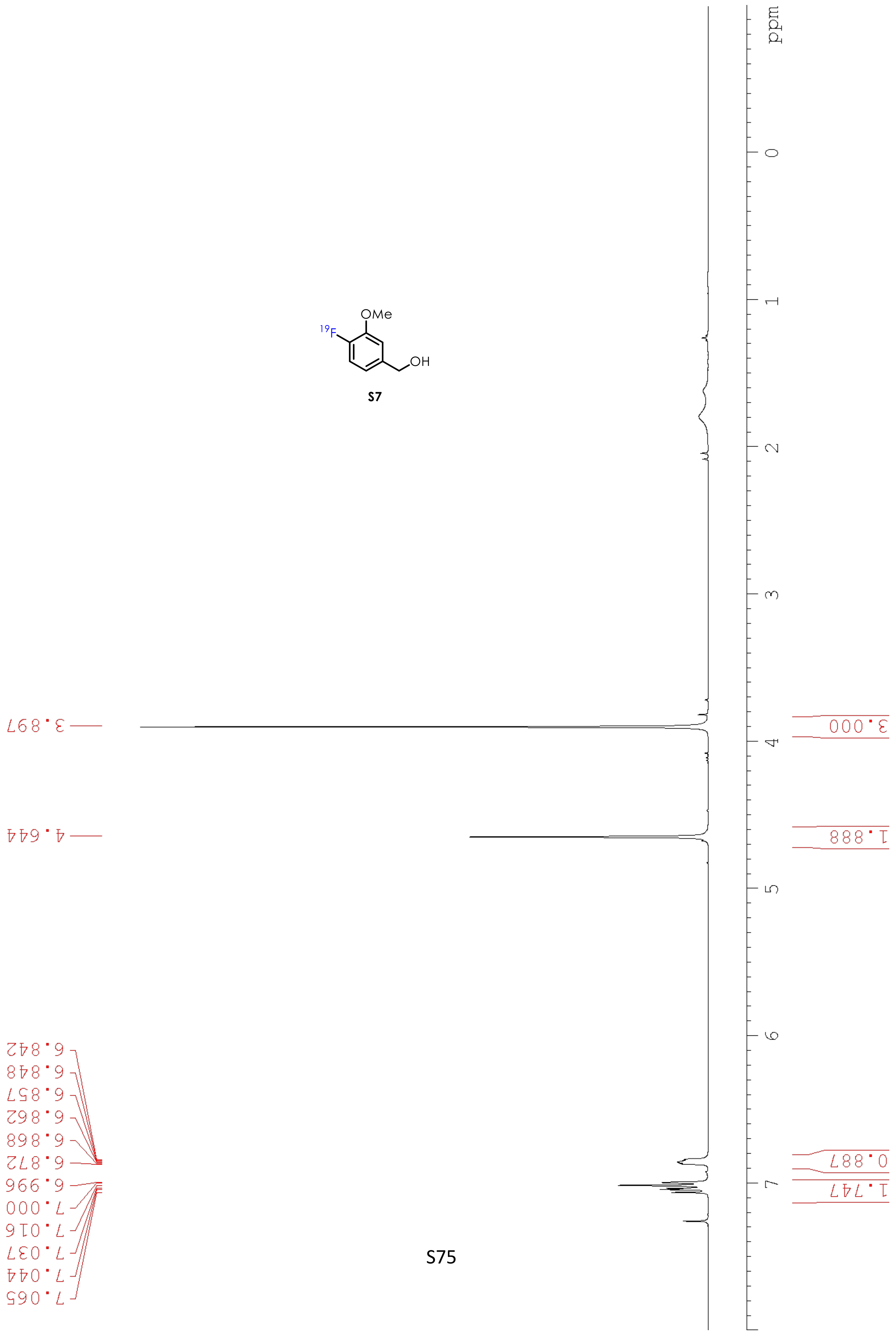
OMe
19F
OH
S7
3.897
4.644
6.842
6.848
6.857
6.862
6.868
6.872
6.996
7.000
7.016
7.037
7.044
7.065
3.000
1.888
0.887
1.747
ppm

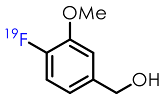
OMe
19F
OH

S7

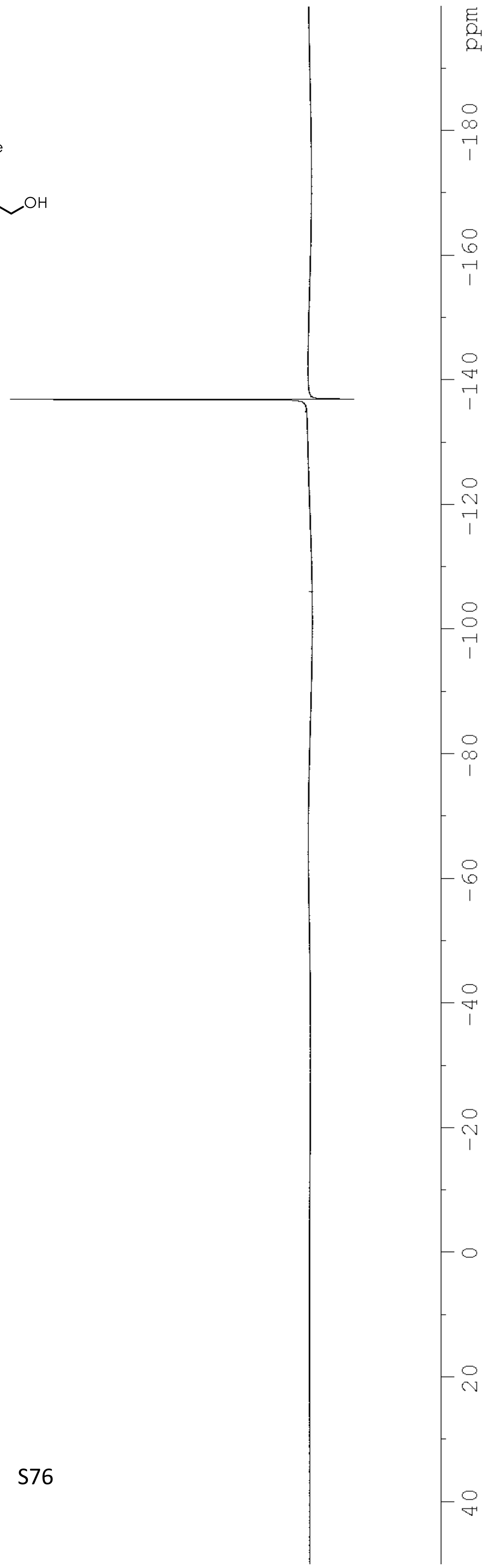
-136.963
-136.952
-136.940
-136.931
-136.923
-136.912
-136.900
ppm
-180
-160
-140
-120
-100
-80
-60
-40
-20
0
20
40

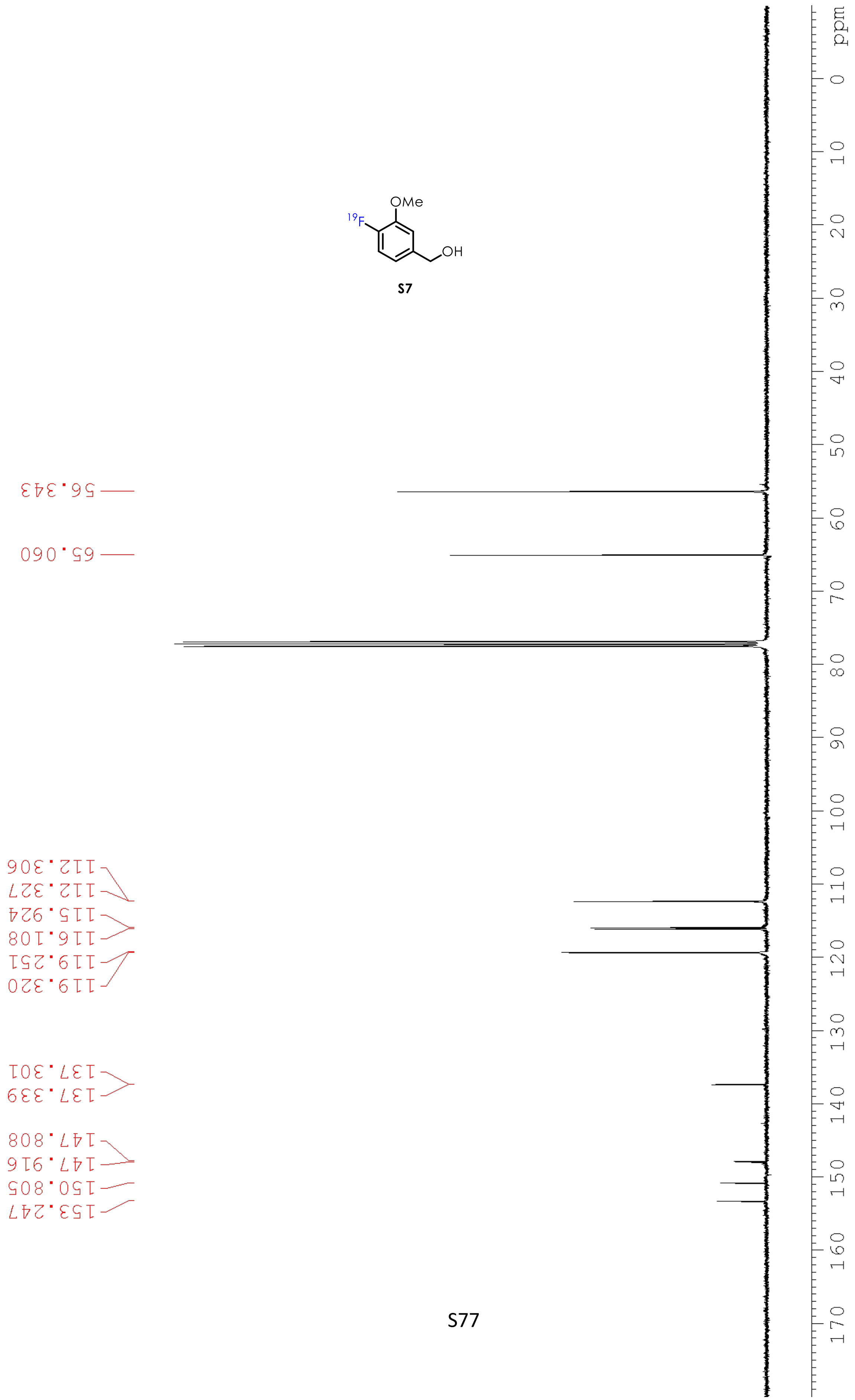

OMe
19F
OH
S7
56.343
65.060
112.306
112.327
115.924
116.108
119.251
119.320
137.301
137.339
147.808
147.916
150.805
153.247
ppm
0
10
20
30
40
50
60
70
80
90
100
110
120
130
140
150
160
170

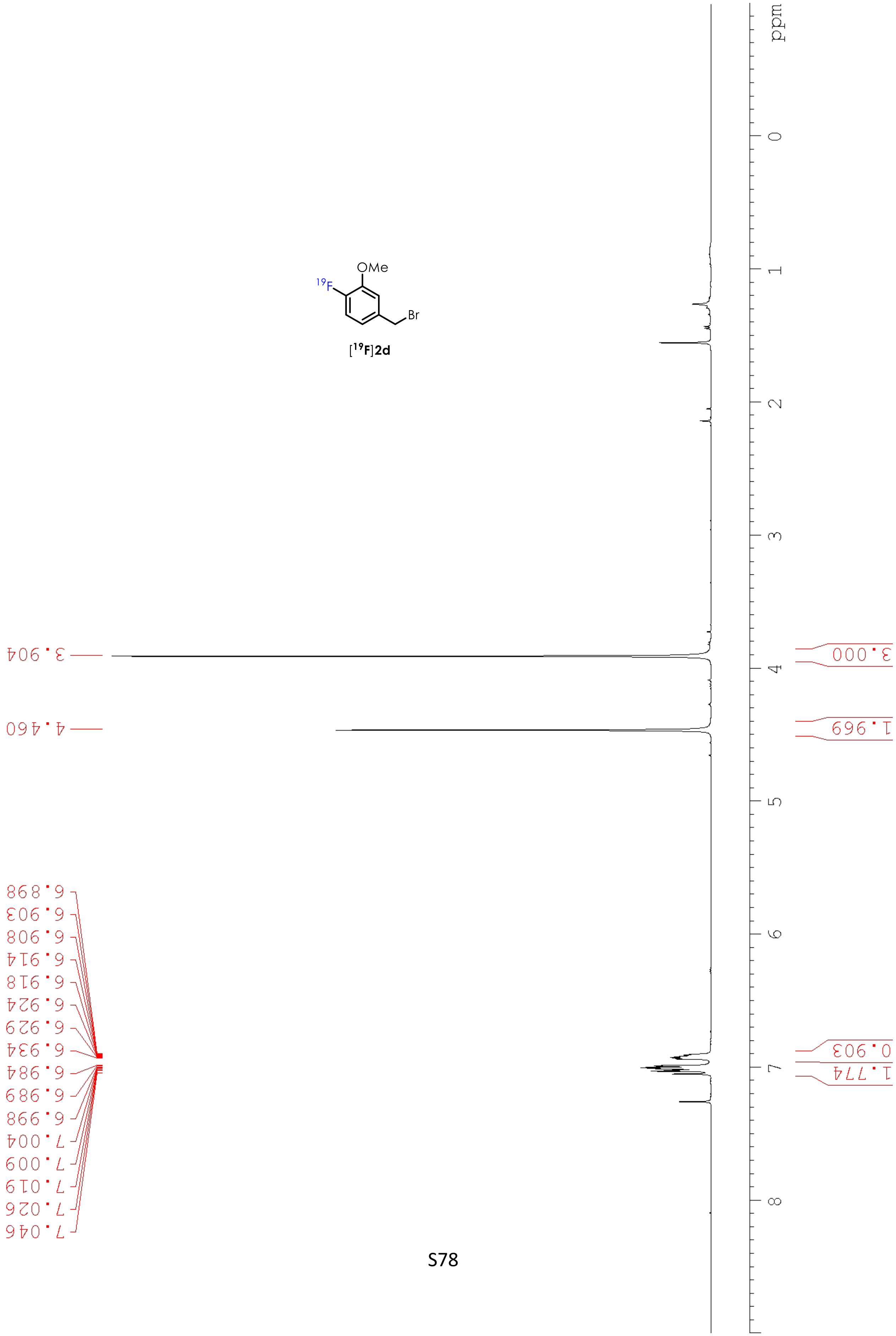

OMe
19F
Br
[19F]2d
7.046
7.026
7.019
7.009
7.004
6.998
6.989
6.984
6.934
6.929
6.924
6.918
6.914
6.908
6.903
6.898
4.460
3.904
1.774
0.903
1.969
3.000
ppm

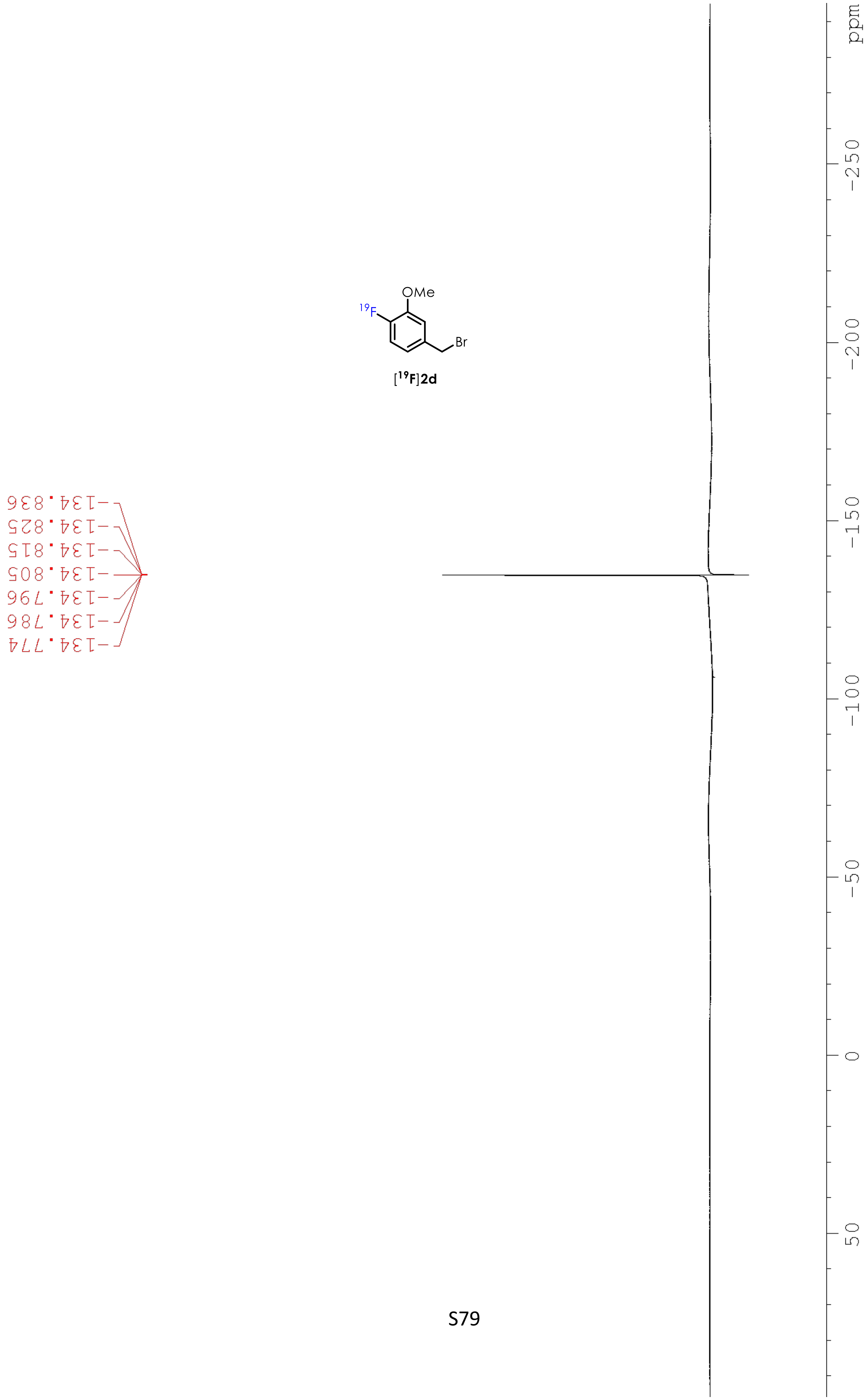
OMe
19F
Br
[19F]2d
-134.836
-134.825
-134.815
-134.805
-134.796
-134.786
-134.774
ppm
-250
-200
-150
-100
-50
0
50

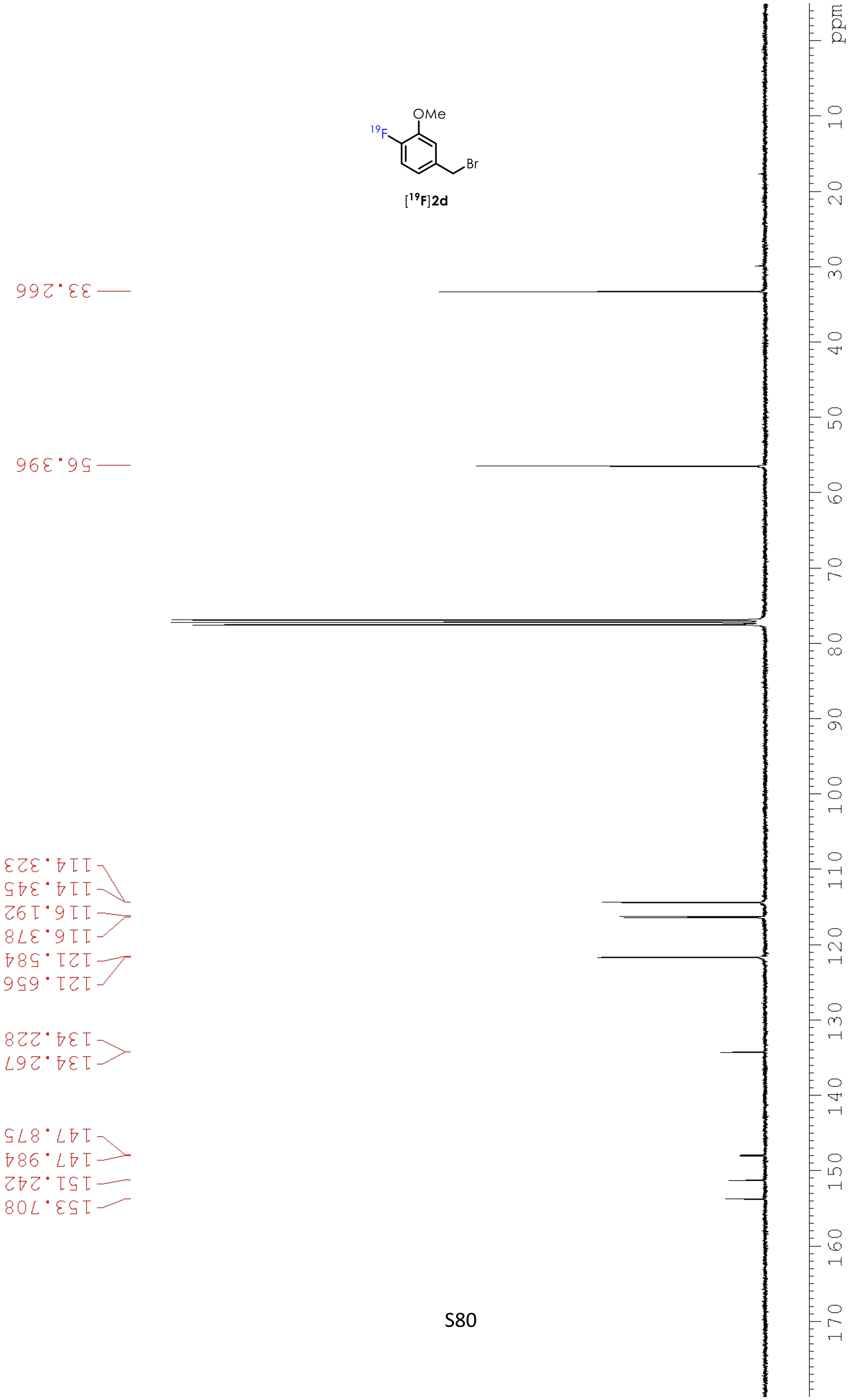
OMe
19F
Br
[19F]2d
33.266
56.396
114.323
114.345
116.192
116.378
121.584
121.656
134.228
134.267
147.875
147.984
151.242
153.708
ppm
10
20
30
40
50
60
70
80
90
100
110
120
130
140
150
160
170

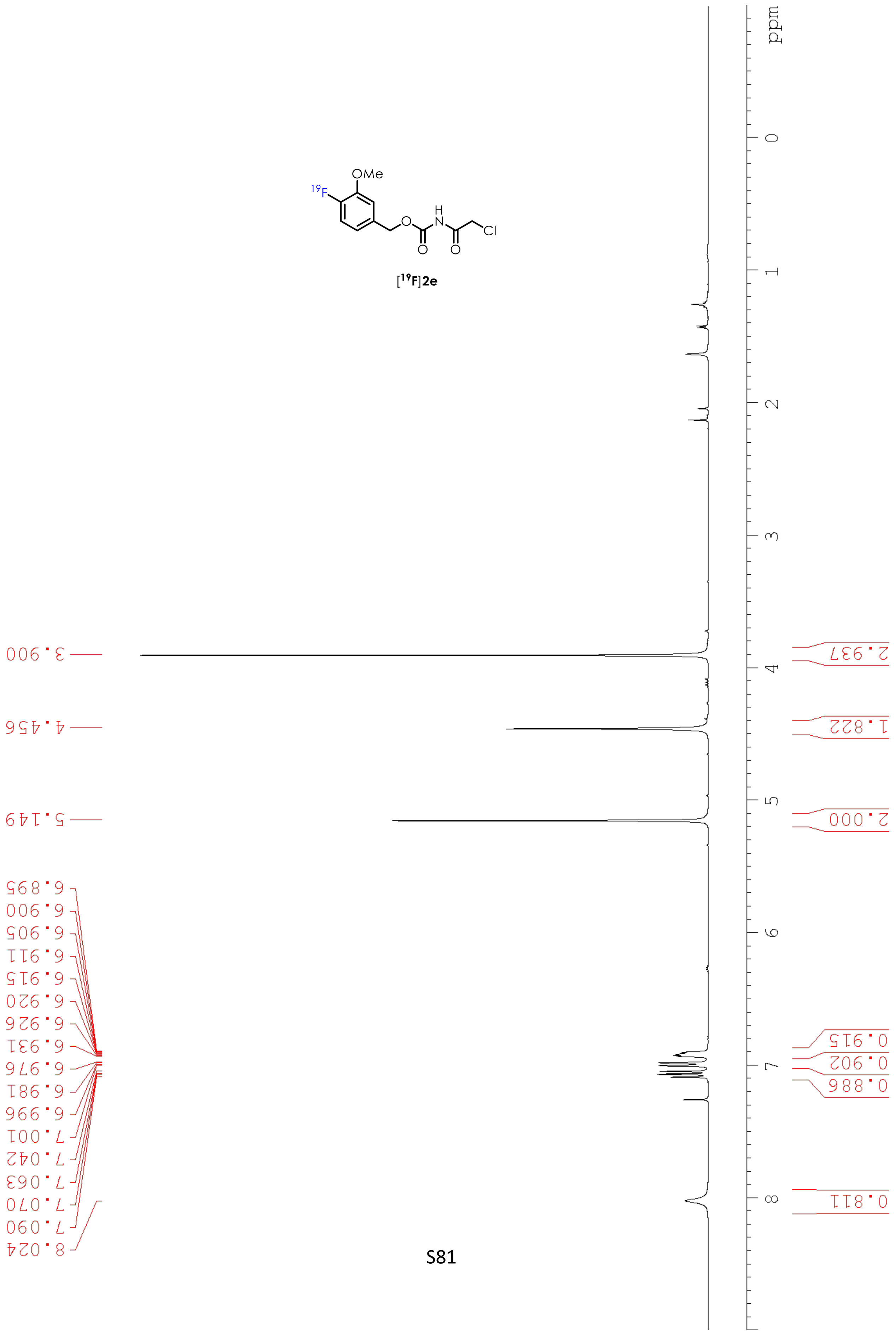
OMe
19F
H
N
O
Cl
[19F]2e
3.900
4.456
5.149
6.895
6.900
6.905
6.911
6.915
6.920
6.926
6.931
6.976
6.981
6.996
7.001
7.042
7.063
7.070
7.090
8.024
2.937
1.822
2.000
0.915
0.902
0.886
0.811
ppm
0
1
2
3
4
5
6
7
8

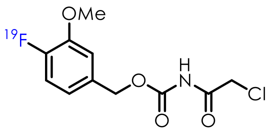
OMe
19F
H
N
O
Cl

[19F]2e

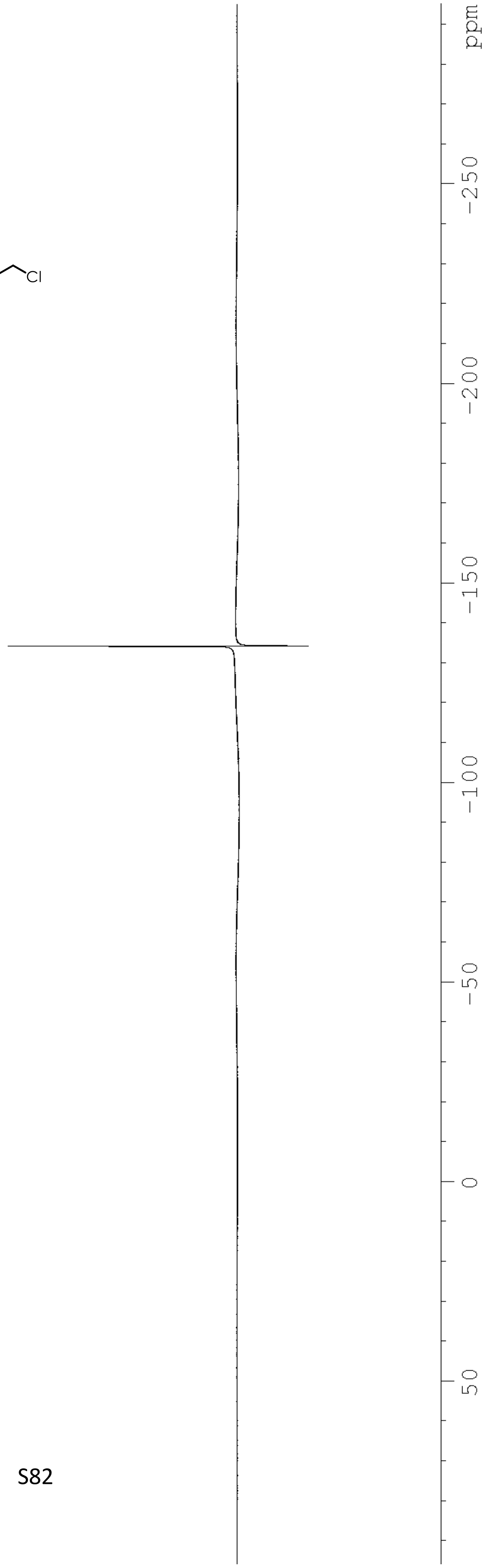
-134.326
-134.314
-134.306
-134.297
-134.287
-134.275
ppm
-250
-200
-150
-100
-50
0
50

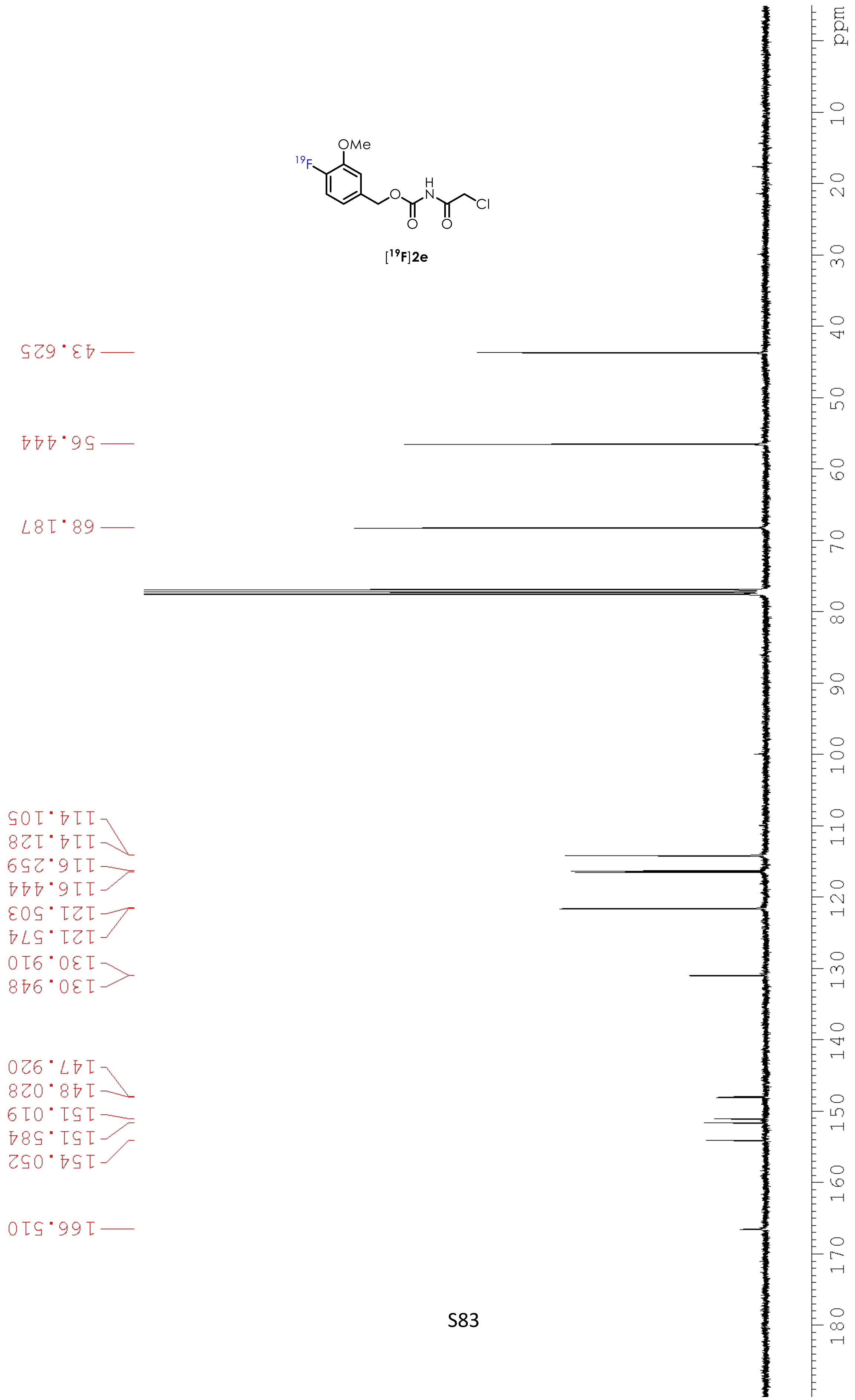
OMe
19F
H
N
O
O
O
Cl
[19F]2e
43.625
56.444
68.187
114.105
114.128
116.259
116.444
121.503
121.574
130.910
130.948
147.920
148.028
151.019
151.584
154.052
166.510
ppm
10
20
30
40
50
60
70
80
90
100
110
120
130
140
150
160
170
180

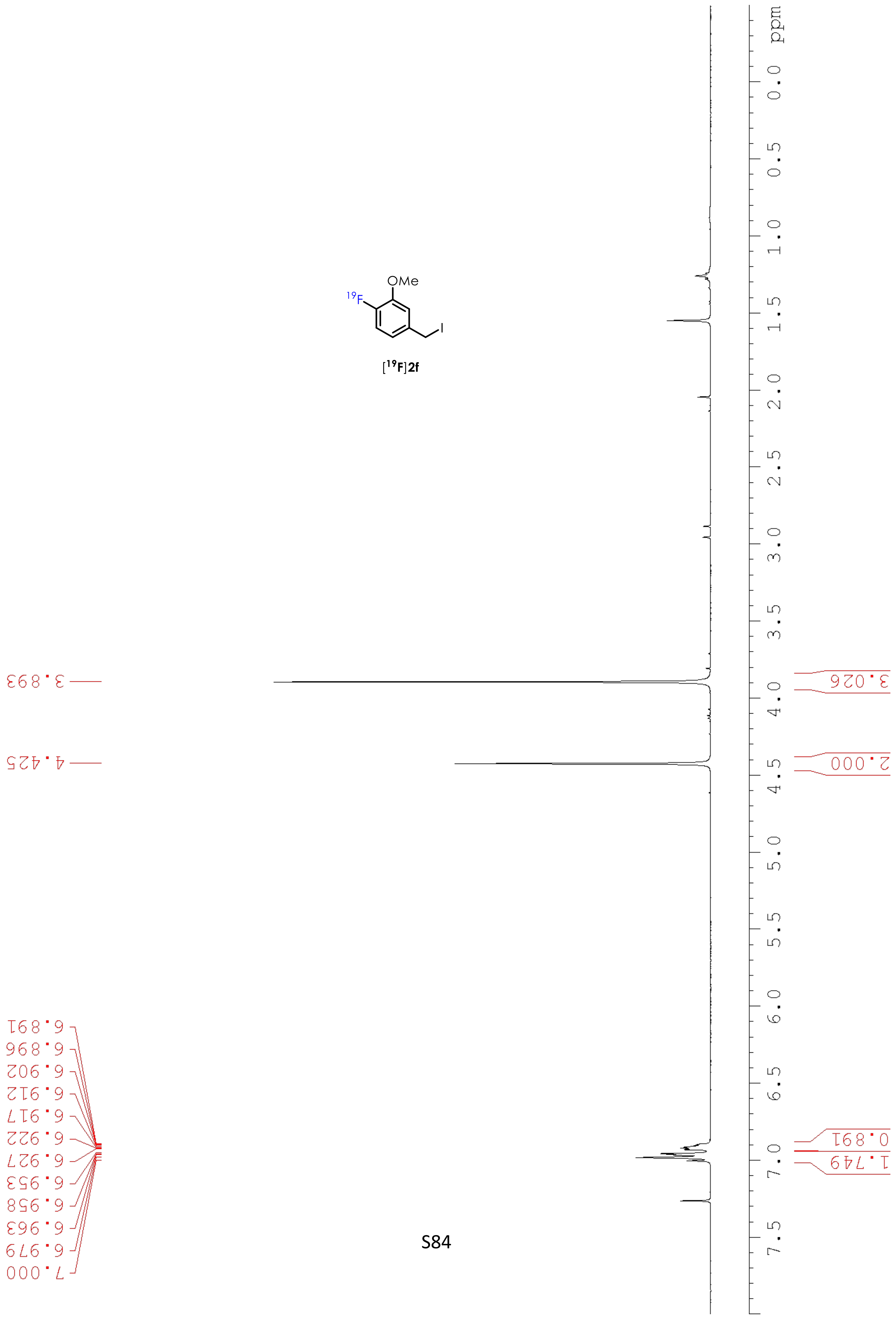

OMe
19F
I
[19F]2f
3.893
4.425
6.891
6.896
6.902
6.912
6.917
6.922
6.927
6.953
6.958
6.963
6.979
7.000
3.026
2.000
0.891
1.749
ppm
0.0
0.5
1.0
1.5
2.0
2.5
3.0
3.5
4.0
4.5
5.0
5.5
6.0
6.5
7.0
7.5

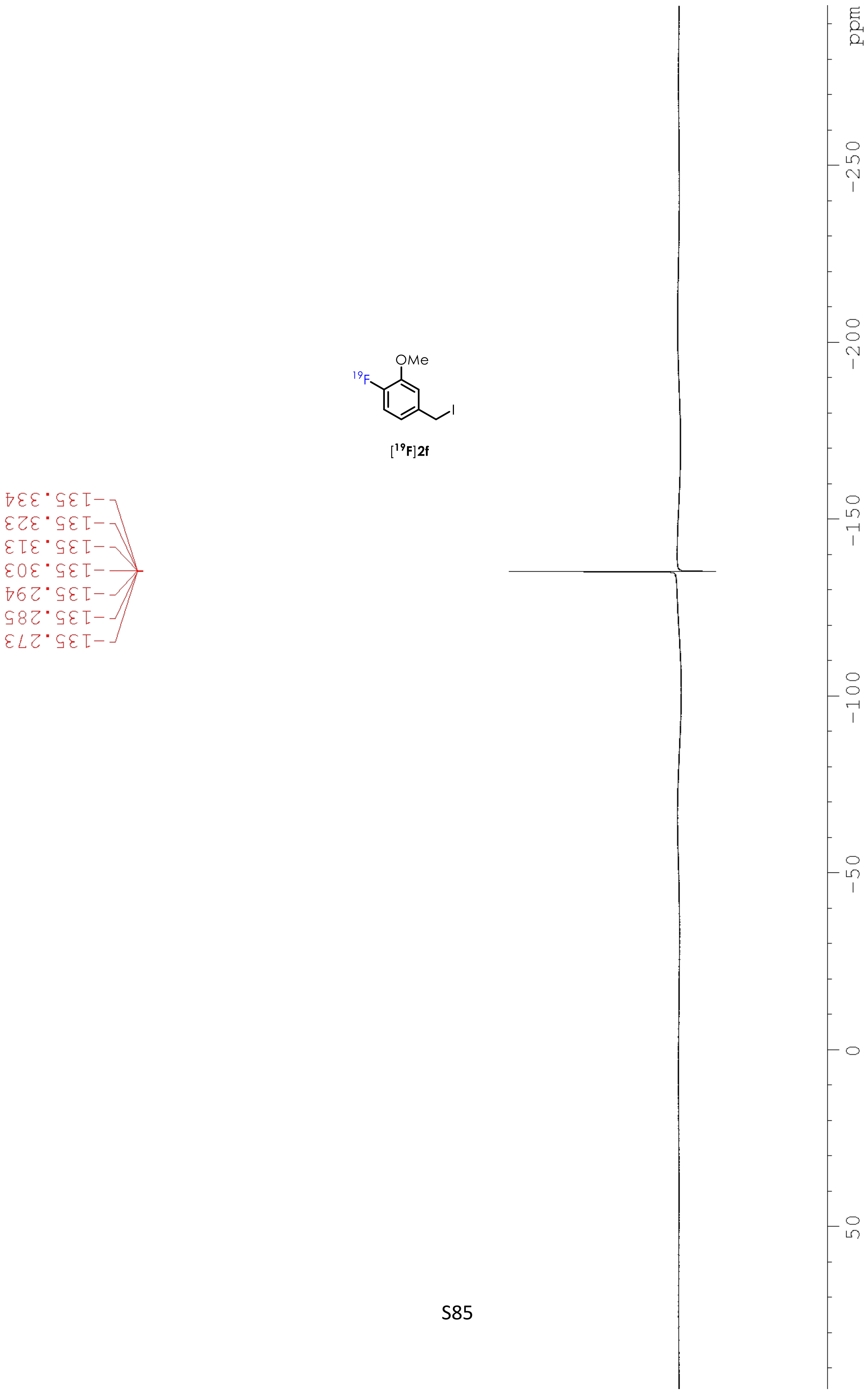
OMe
19F
I
[19F]2f
-135.334
-135.323
-135.313
-135.303
-135.294
-135.285
-135.273
ppm
-250
-200
-150
-100
-50
0
50

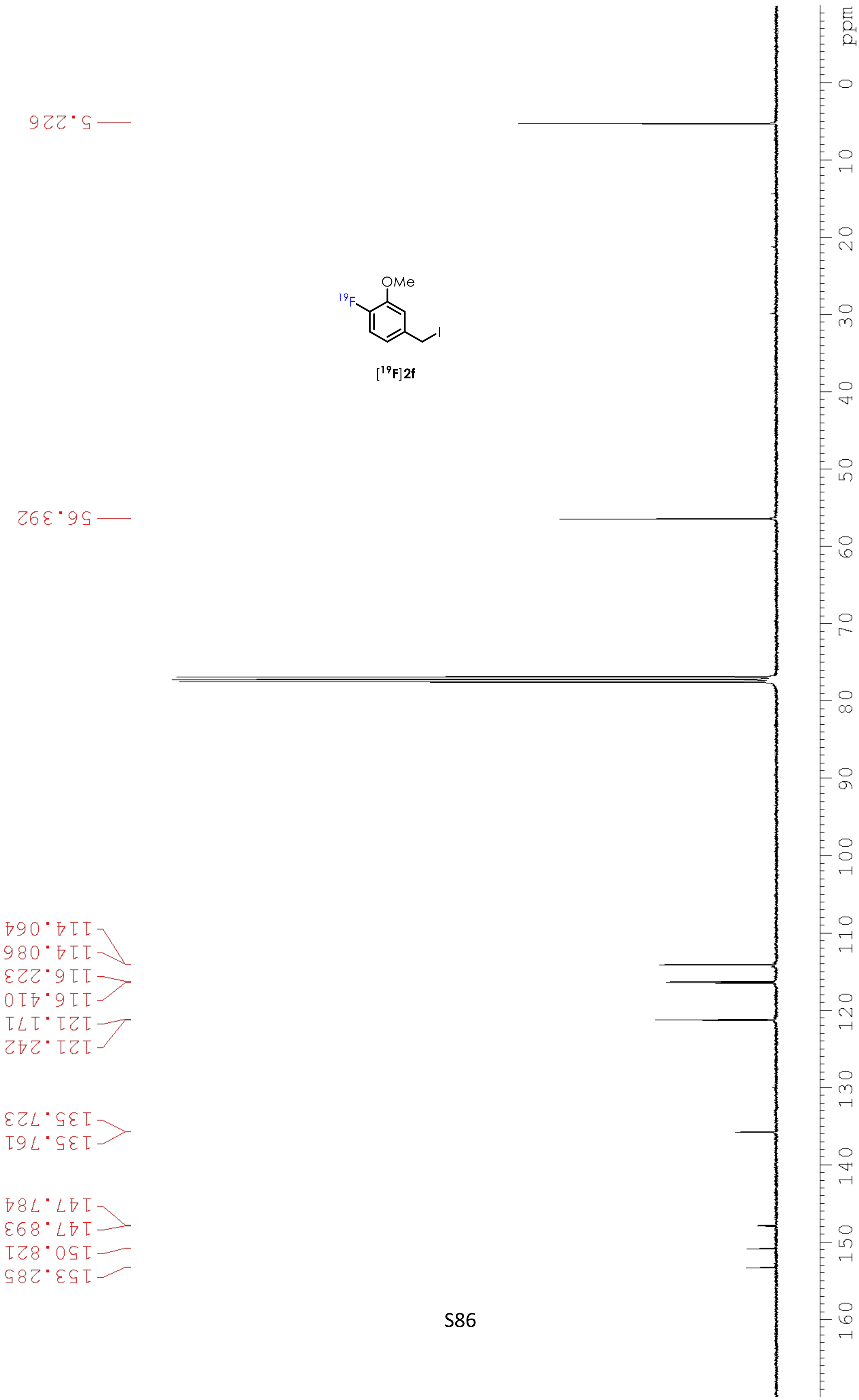

5.226
56.392
114.064
114.086
116.223
116.410
121.171
121.242
135.723
135.761
147.784
147.893
150.821
153.285
ppm
0
10
20
30
40
50
60
70
80
90
100
110
120
130
140
150
160
OMe
19F
I

[19F]2f

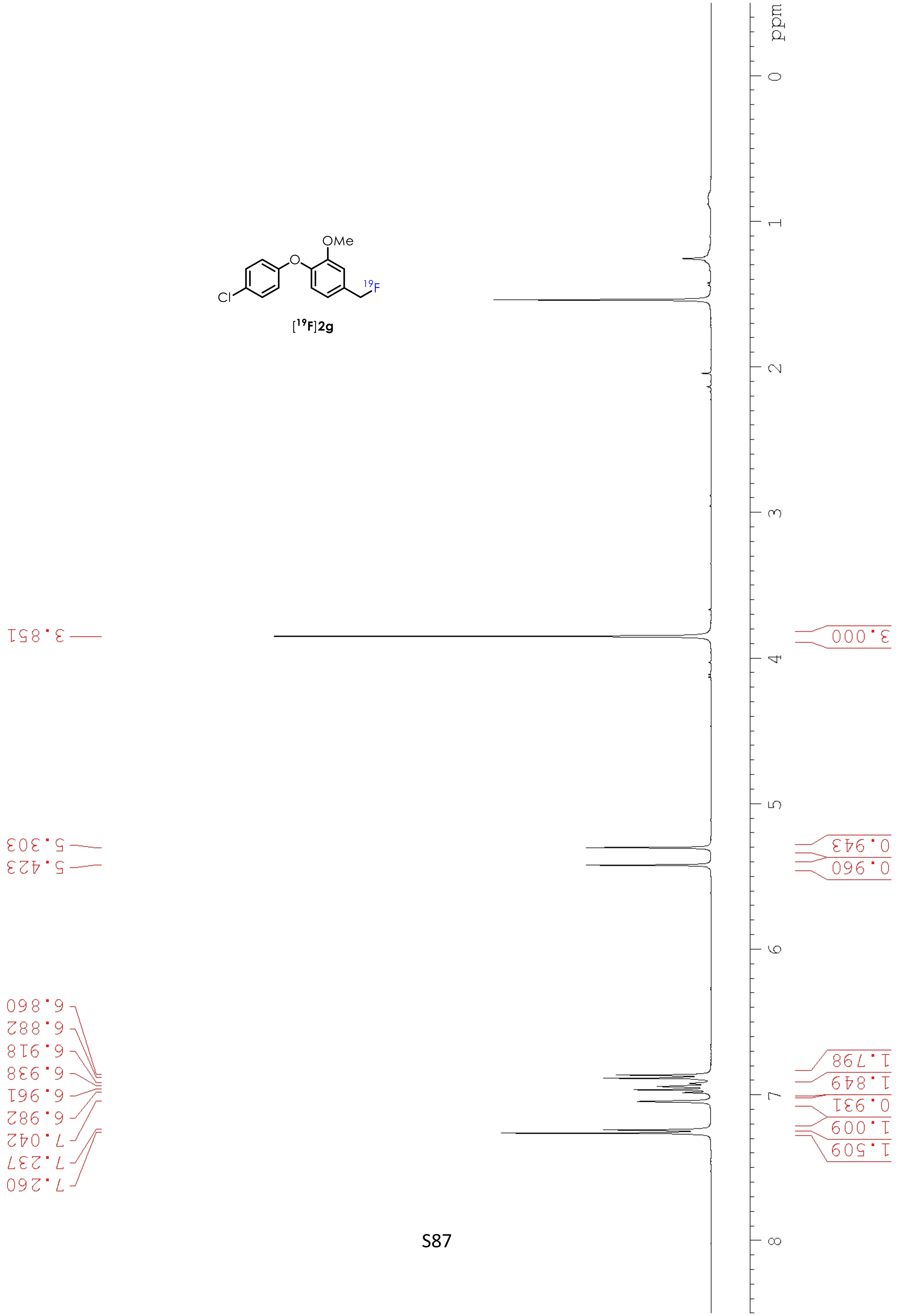

OMe
Cl
19F
[19F]2g
ppm
0
1
2
3
4
5
6
7
8
3.851
5.303
5.423
6.860
6.882
6.918
6.938
6.961
6.982
7.042
7.237
7.260
3.000
0.943
0.960
1.798
1.849
0.931
1.009
1.509

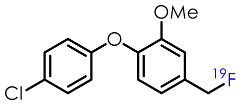

[^{19}F]**2g**

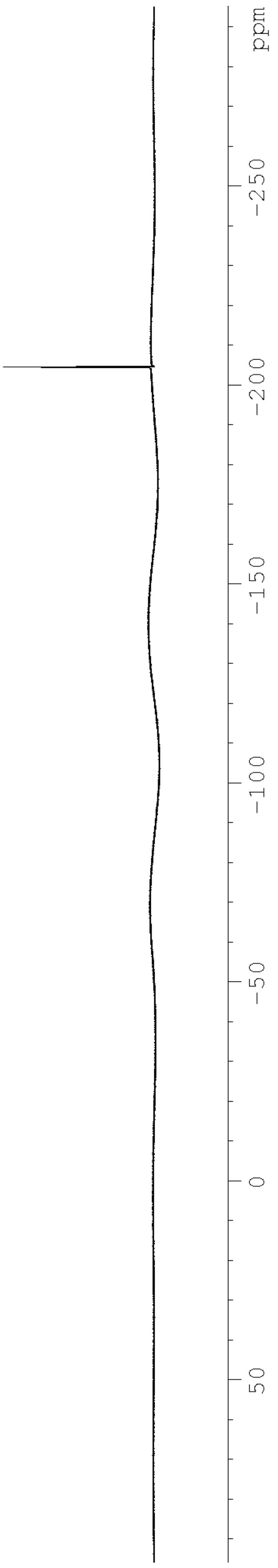

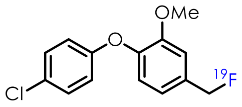
OMe
Cl
O
19F

[19F]2g

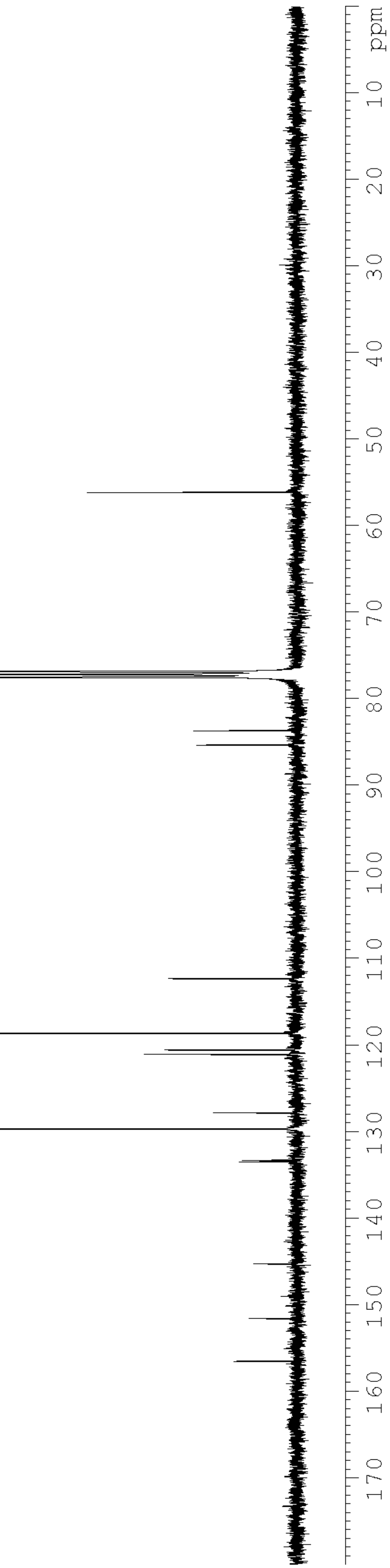
56.147
83.653
85.311
112.295
112.350
118.623
120.531
120.590
121.060
127.811
129.665
133.458
156.507
ppm
10
20
30
40
50
60
70
80
90
100
110
120
130
140
150
160
170

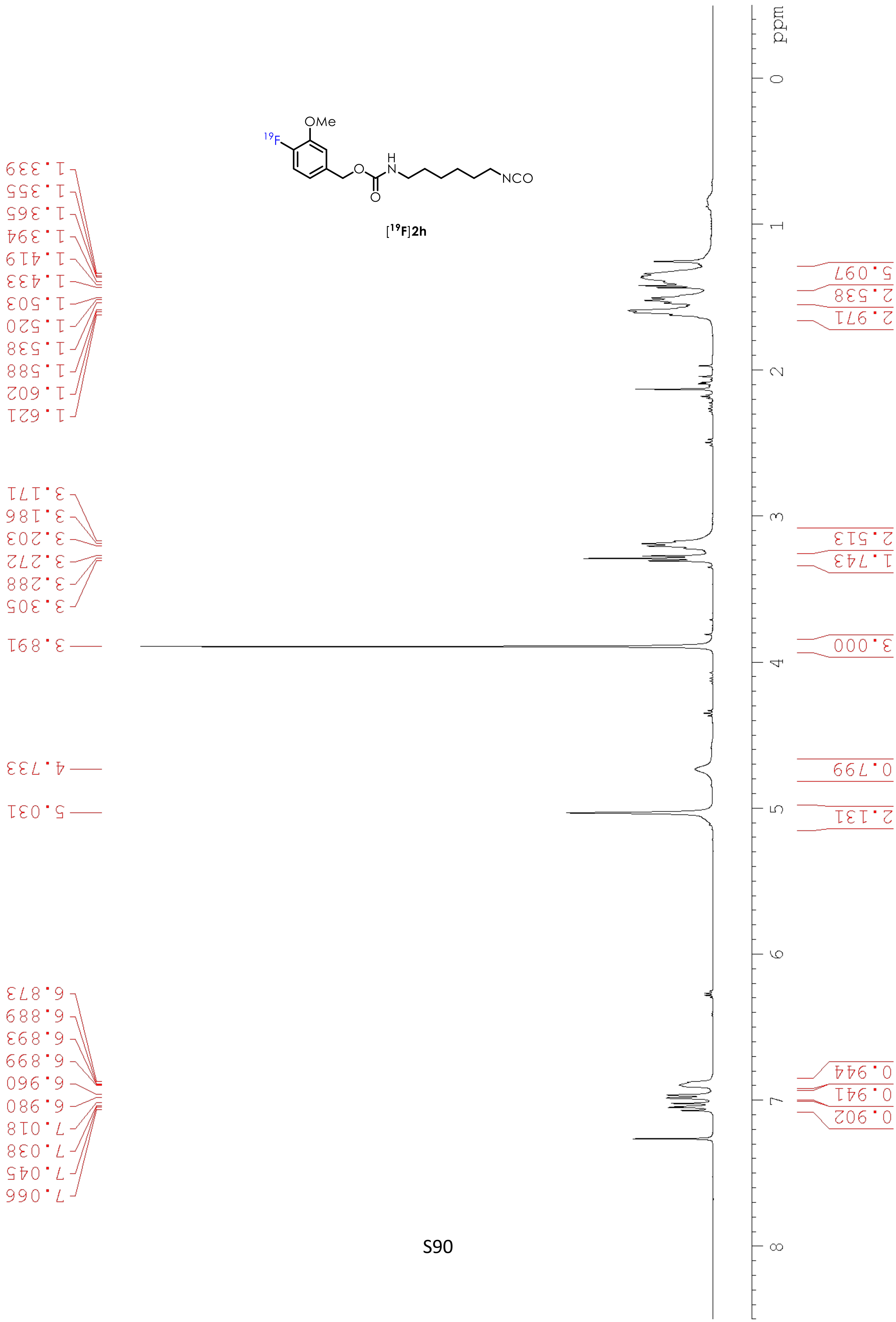

[^{19}F]**2h**

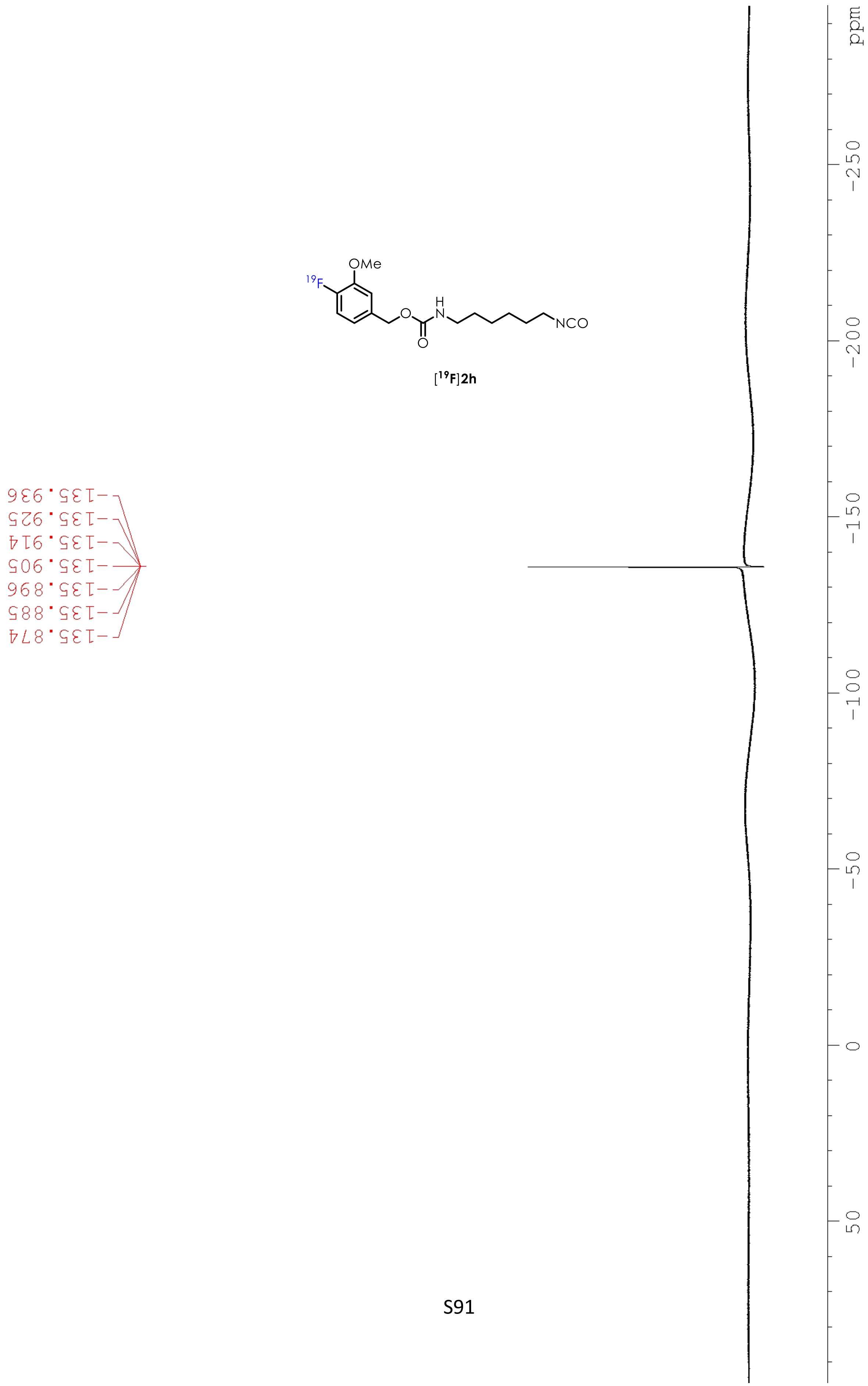
-135.936
-135.925
-135.914
-135.905
-135.896
-135.885
-135.874
ppm
-250
-200
-150
-100
-50
0
50
OMe
19F
H
N
O
NCO

[^{19}F]**2h**

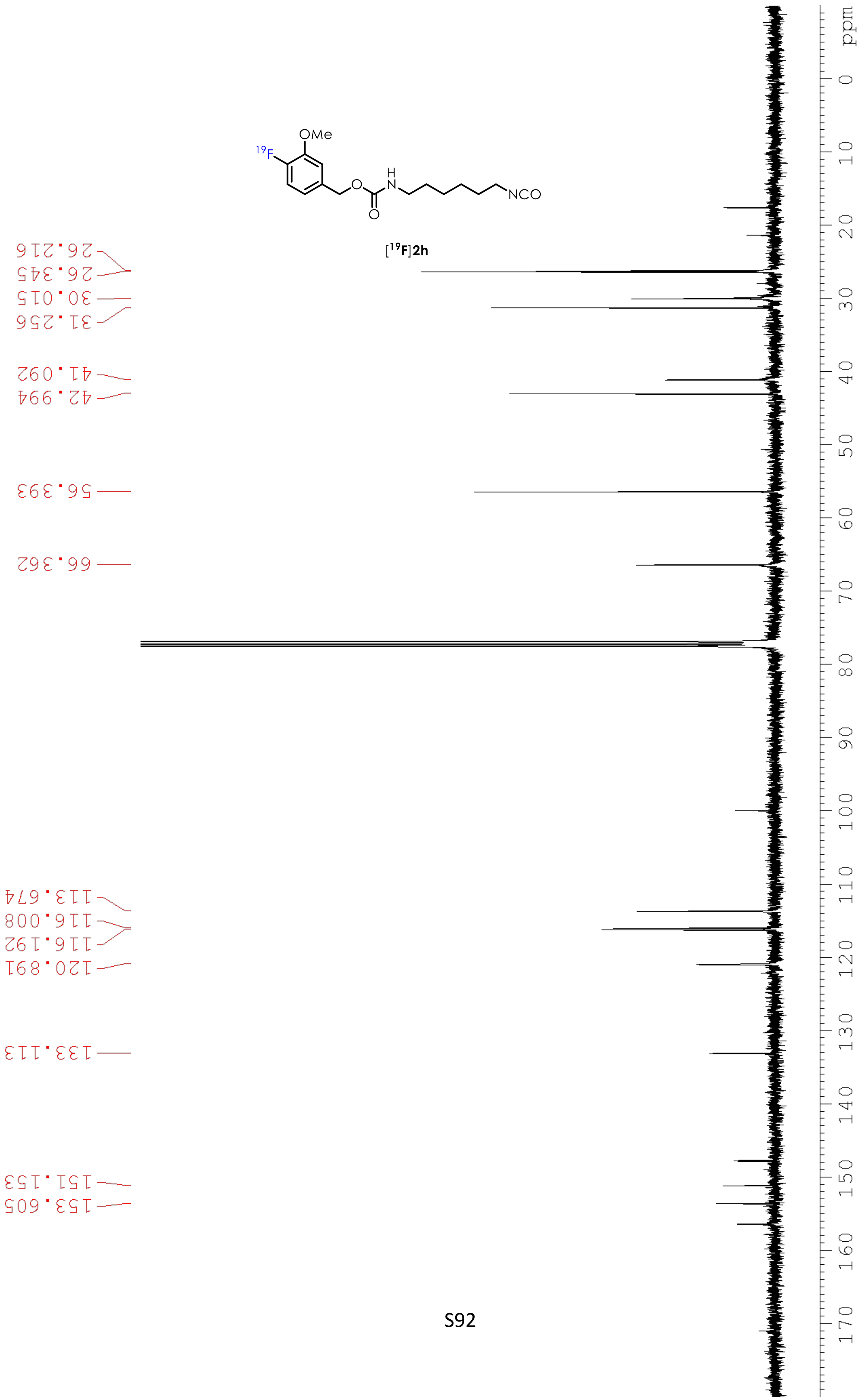
OMe
19F
H
N
O
O
O
NCO
[19F]2h
26.216
26.345
30.015
31.256
41.092
42.994
56.393
66.362
113.674
116.008
116.192
120.891
133.113
151.153
153.605
ppm
0
10
20
30
40
50
60
70
80
90
100
110
120
130
140
150
160
170

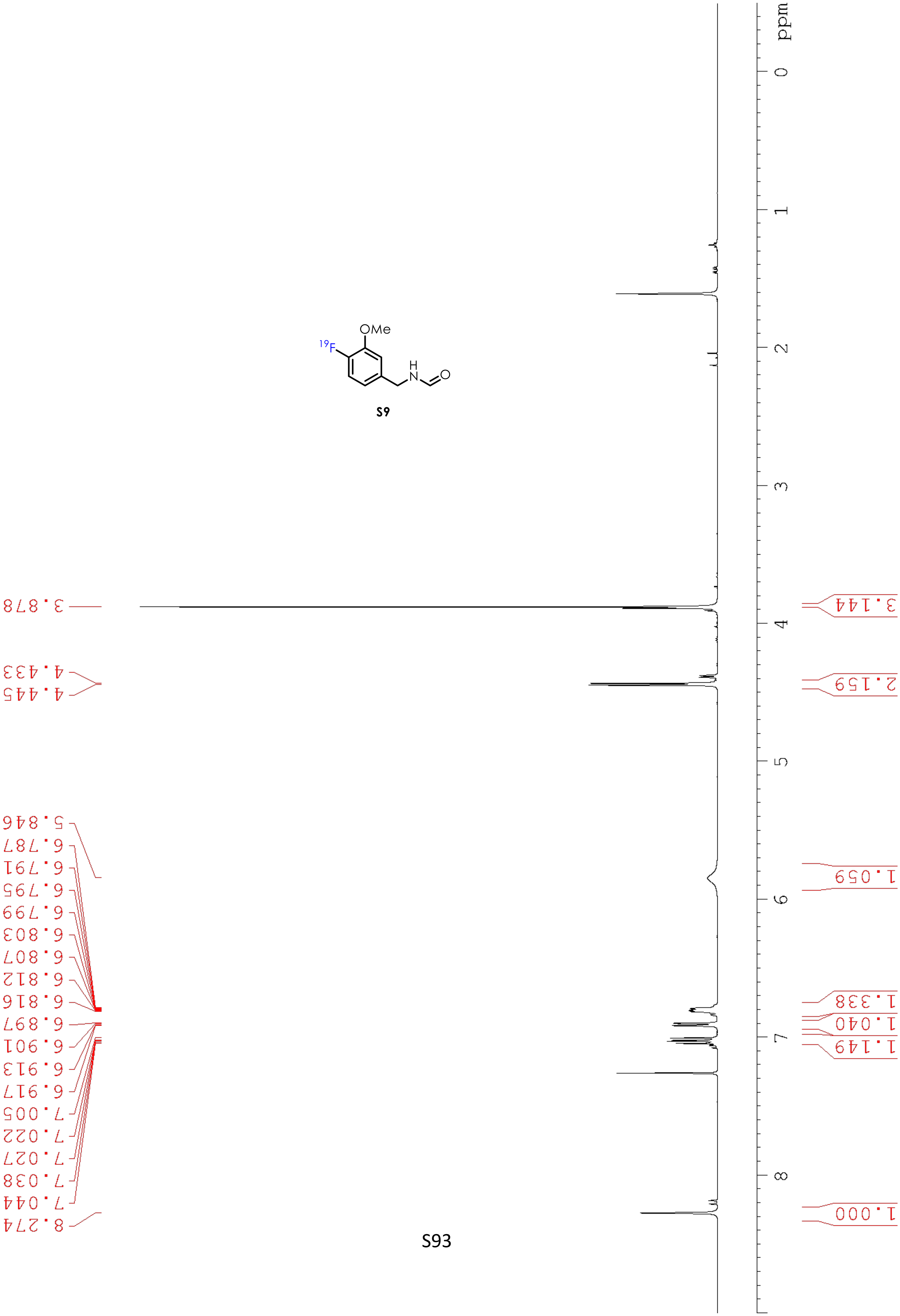
OMe
19F
H
N
O
S9
3.878
4.433
4.445
5.846
6.787
6.791
6.795
6.799
6.803
6.807
6.812
6.816
6.897
6.901
6.913
6.917
7.005
7.022
7.027
7.038
7.044
8.274
3.144
2.159
1.059
1.338
1.040
1.149
1.000
ppm

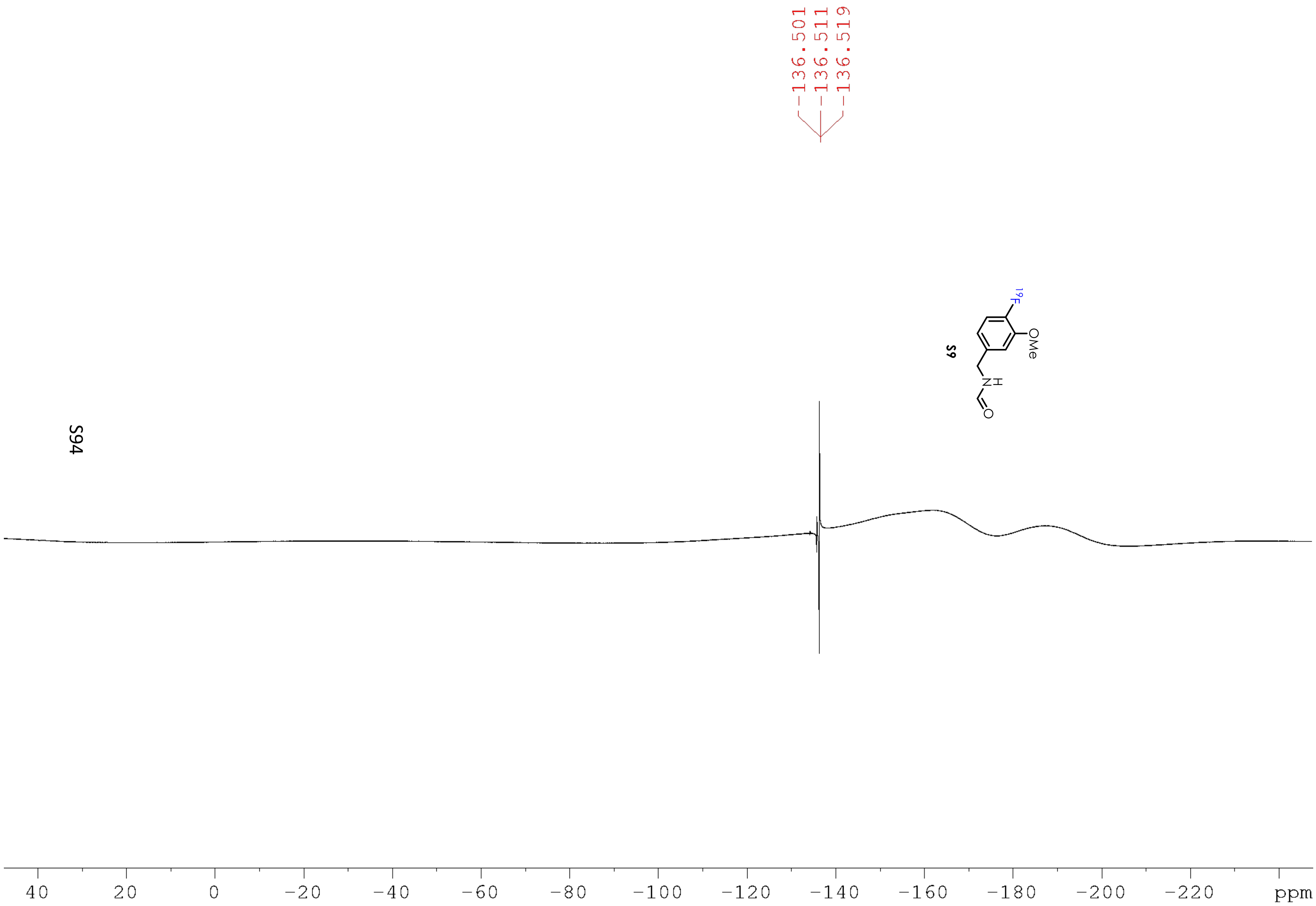

-136.501
-136.511
-136.519
19F
OMe
H
N
O
40 20 0 -20 -40 -60 -80 -100 -120 -140 -160 -180 -200 -220 ppm

S9

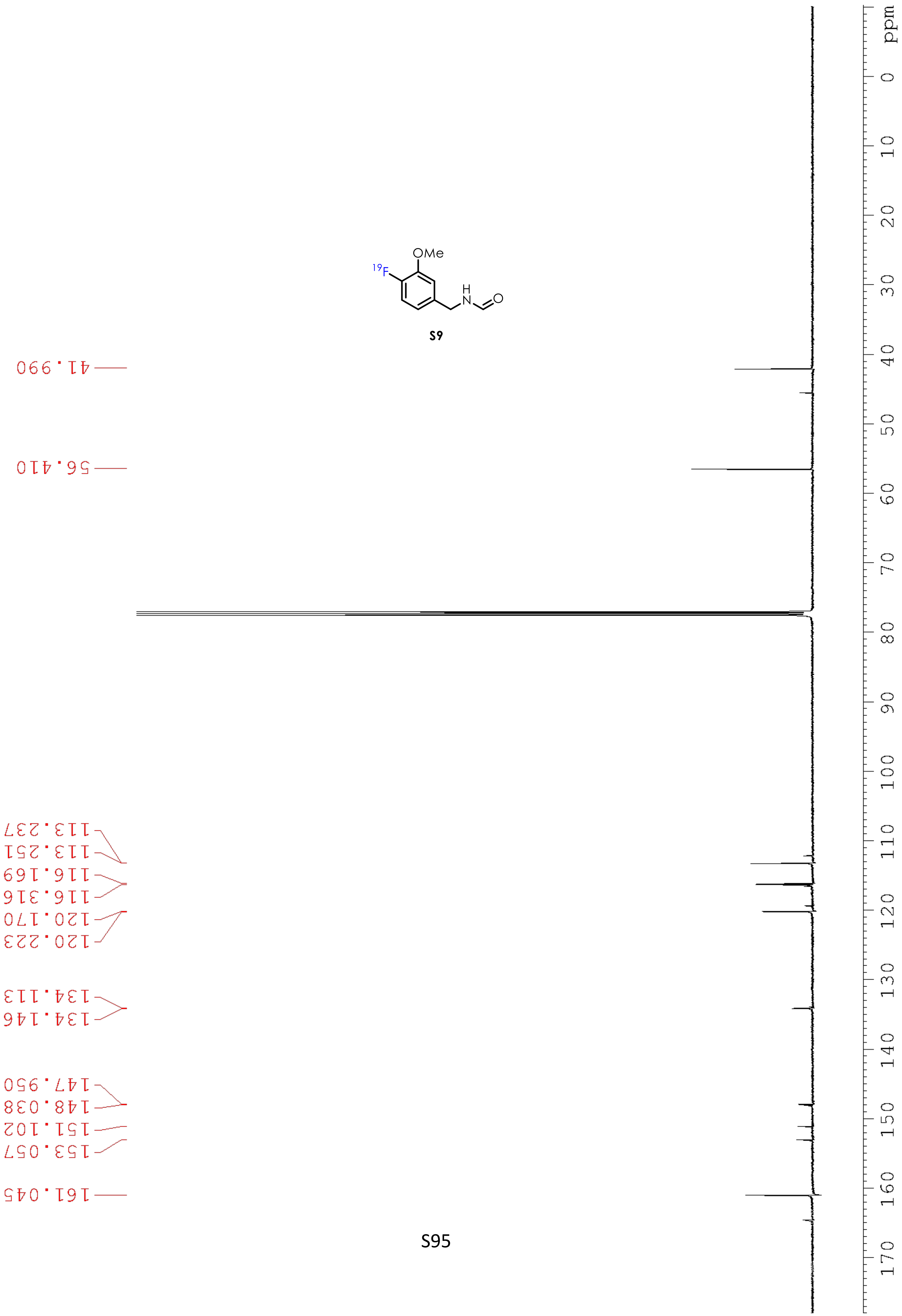
OMe
19F
H
N
O
S9
41.990
56.410
113.237
113.251
116.169
116.316
120.170
120.223
134.113
134.146
147.950
148.038
151.102
153.057
161.045
ppm

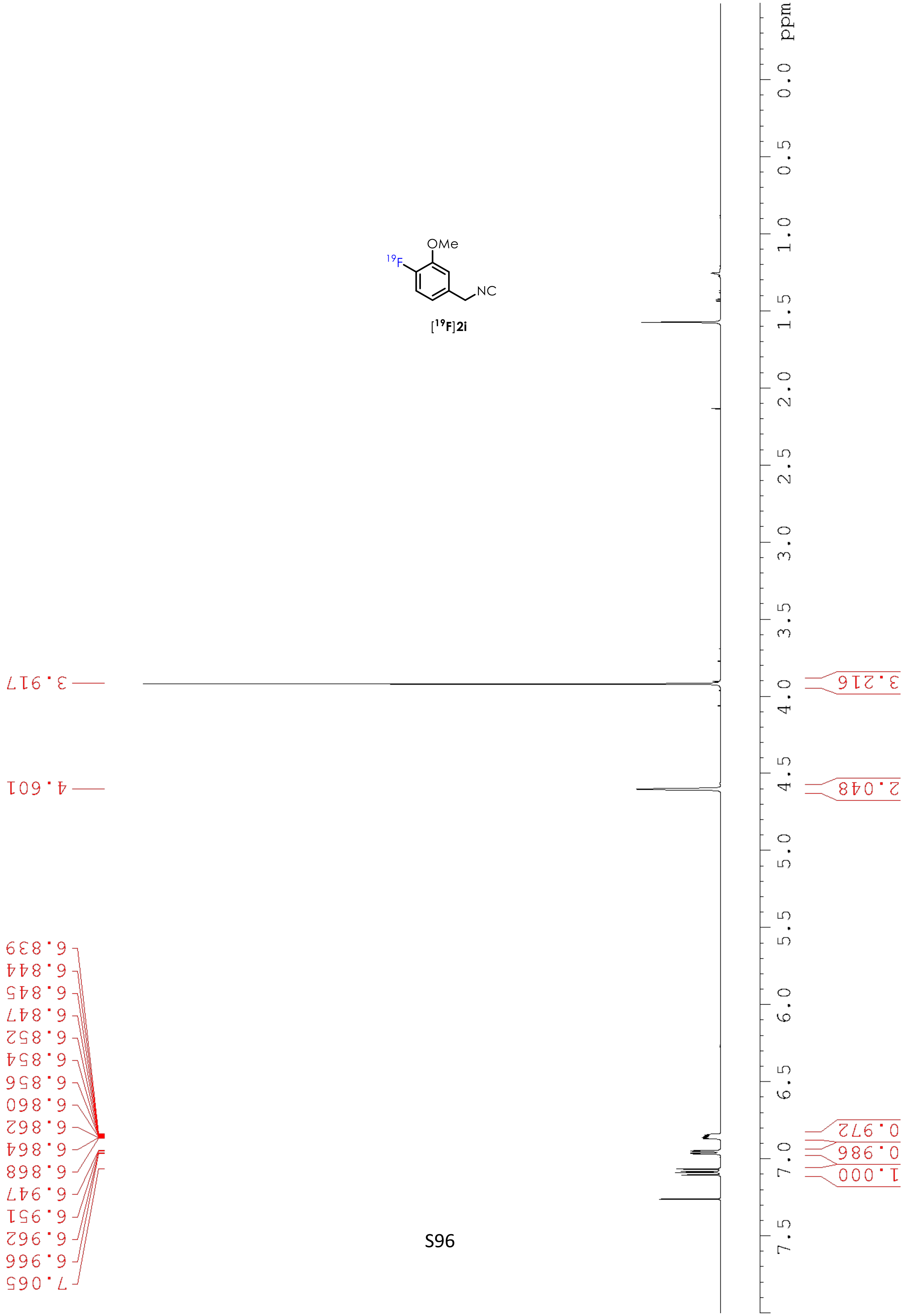
OMe
19F
NC
[19F]2i
ppm
3.917
4.601
6.839
6.844
6.845
6.847
6.852
6.854
6.856
6.860
6.862
6.864
6.868
6.947
6.951
6.962
6.966
7.065
3.216
2.048
0.972
0.986
1.000

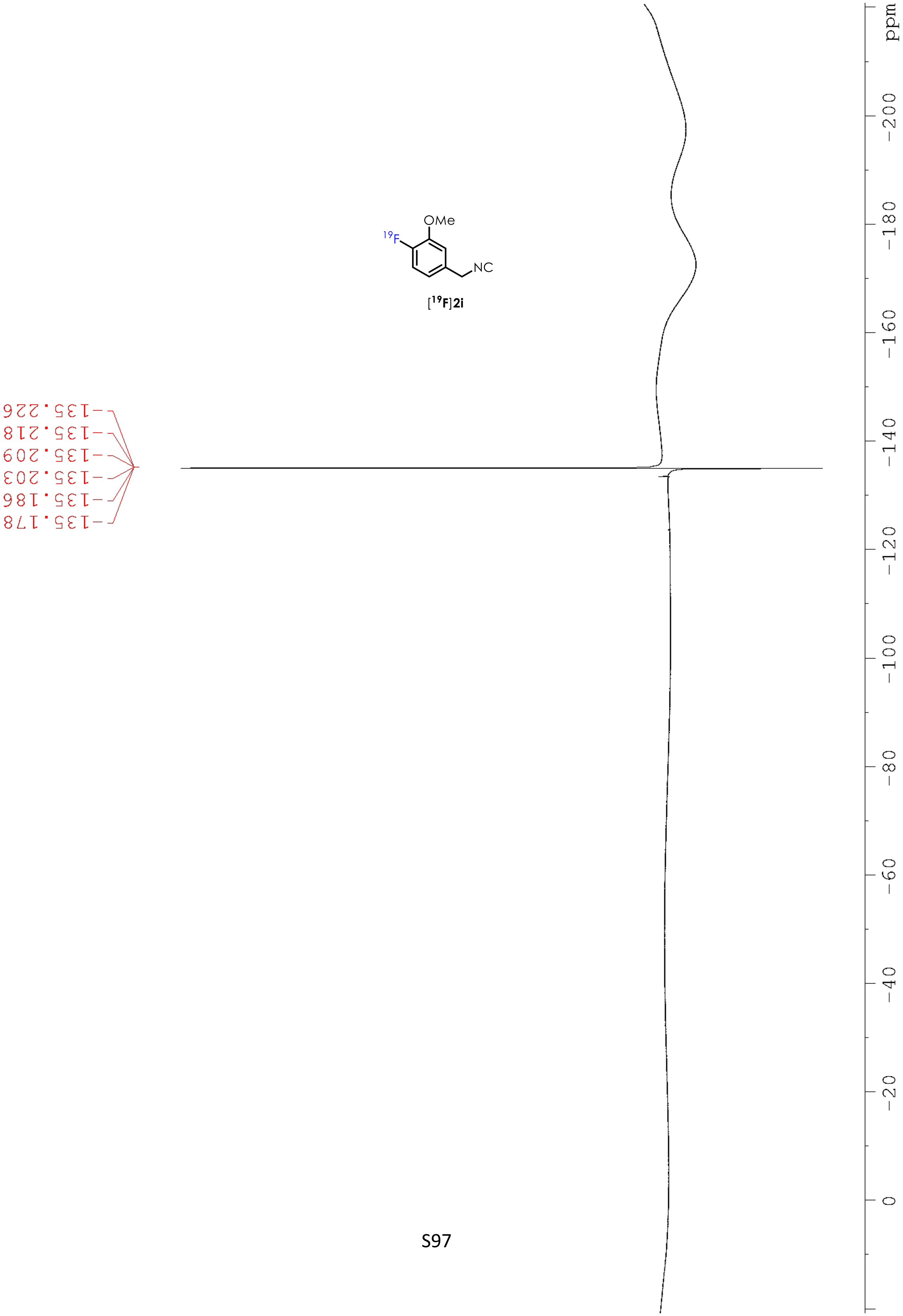

OMe
19F
NC
[19F]2i
-135.226
-135.218
-135.209
-135.203
-135.186
-135.178
ppm
-200
-180
-160
-140
-120
-100
-80
-60
-40
-20
0

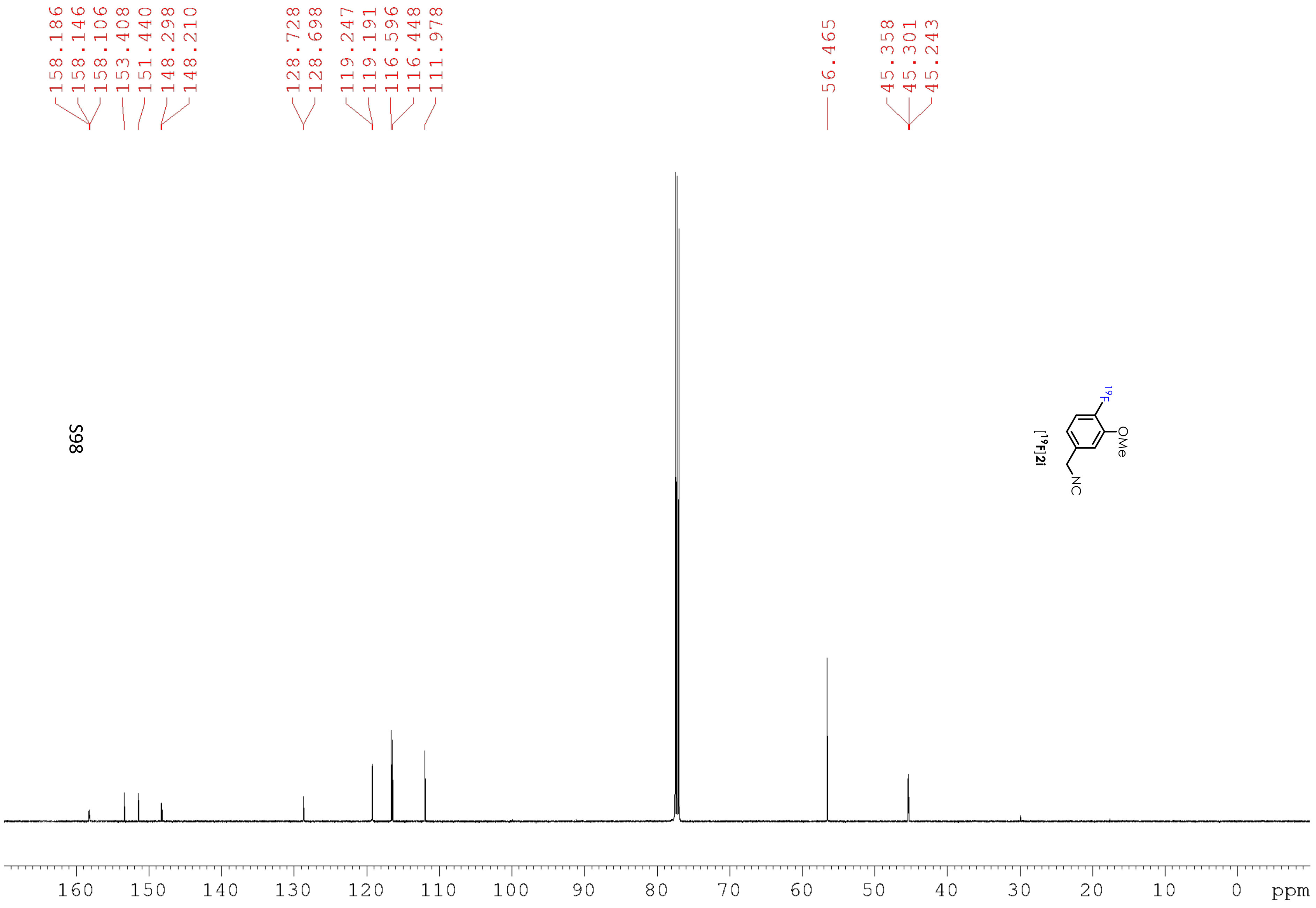

158.186
158.146
158.106
153.408
151.440
148.298
148.210
128.728
128.698
119.247
119.191
116.596
116.448
111.978
56.465
45.358
45.301
45.243
OMe
19F
NC
[19F]2i
160
150
140
130
120
110
100
90
80
70
60
50
40
30
20
10
0
ppm

References

(1) Chen, W.; Huang, Z.; Tay, N. E. S.; Giglio, B.; Wang, M.; Wang, H.; Wu, Z.; Nicewicz, D. A.; Li, Z. Direct arene C–H fluorination with ¹⁸F[−] via organic photoredox catalysis. *Science* **2019**, *364* (6446), 1170-1174. DOI: doi:10.1126/science.aav7019.

(2) Tay, N. E. S.; Chen, W.; Levens, A.; Pistritto, V. A.; Huang, Z.; Wu, Z.; Li, Z.; Nicewicz, D. A. 19F- and 18F-arene deoxyfluorination via organic photoredox-catalysed polarity-reversed nucleophilic aromatic substitution. *Nature Catalysis* **2020**, *3* (9), 734-742. DOI: 10.1038/s41929-020-0495-0.

(3) Coenen, H. H.; Gee, A. D.; Adam, M.; Antoni, G.; Cutler, C. S.; Fujibayashi, Y.; Jeong, J. M.; Mach, R. H.; Mindt, T. L.; Pike, V. W.; et al. Consensus nomenclature rules for radiopharmaceutical chemistry — Setting the record straight. *Nuclear Medicine and Biology* **2017**, *55*, v-xi. DOI: https://doi.org/10.1016/j.nucmedbio.2017.09.004.

(4) Herth, M. M.; Ametamey, S.; Antuganov, D.; Bauman, A.; Berndt, M.; Brooks, A. F.; Bormans, G.; Choe, Y. S.; Gillings, N.; Häfeli, U. O.; et al. On the consensus nomenclature rules for radiopharmaceutical chemistry – Reconsideration of radiochemical conversion. *Nuclear Medicine and Biology* **2021**, *93*, 19-21. DOI: https://doi.org/10.1016/j.nucmedbio.2020.11.003.

(5) Li, M.; Ma, X.; Molnar, C. J.; Wang, S.; Wu, Z.; Popik, V. V.; Li, Z. Modular PET Agent Construction Strategy through Strain-Promoted Double-Click Reagent with Efficient Photoclick Step. *Bioconjugate Chemistry* **2022**, *33* (11), 2088-2096. DOI: 10.1021/acs.bioconjchem.2c00427.
